# A multi-omics study of the grapevine-downy mildew (*Plasmopara viticola*) pathosystem unveils a complex protein coding- and noncoding-based arms race during infection

**DOI:** 10.1038/s41598-018-19158-8

**Published:** 2018-01-15

**Authors:** Matteo Brilli, Elisa Asquini, Mirko Moser, Pier Luigi Bianchedi, Michele Perazzolli, Azeddine Si-Ammour

**Affiliations:** 1 Department of Genomics and Biology of Fruit Crops, Research and Innovation Centre, Fondazione Edmund Mach, Via E. Mach 1, 38010 San Michele all’Adige (TN), Italy; 2 Present Address: Department of Agronomy, Food, Natural Resources, Animals and Environment (DAFNAE), University of Padova, Agripolis, V.le dell’Università, 16, 35020 Legnaro (PD), Italy

## Abstract

Fungicides are applied intensively to prevent downy mildew infections of grapevines (*Vitis vinifera*) with high impact on the environment. In order to develop alternative strategies we sequenced the genome of the oomycete pathogen *Plasmopara viticola* causing this disease. We show that it derives from a *Phytophthora*-like ancestor that switched to obligate biotrophy by losing genes involved in nitrogen metabolism and γ-Aminobutyric acid catabolism. By combining multiple omics approaches we characterized the pathosystem and identified a RxLR effector that trigger an immune response in the wild species *V*. *riparia*. This effector is an ideal marker to screen novel grape resistant varieties. Our study reveals an unprecedented bidirectional noncoding RNA-based mechanism that, in one direction might be fundamental for *P*. *viticola* to proficiently infect its host, and in the other might reduce the effects of the infection on the plant.

## Introduction

Grapevine (*Vitis vinifera* L.) is an important commodity and comprises varieties for wine production and table grape for human consumption^1^. Wine production is a very lucrative activity and the world wine trade is worth almost US $30 billion. France, Italy and Spain are the largest European wine producing countries representing altogether half of the world production (http://www.oiv.int/). Grapevines can be infected by a myriad of plant pathogens at all growth stages and in order to secure harvest large quantities of agrochemicals are used to control their spread^2^. The treatments against powdery and downy mildews, including the oomycete *Plasmopara viticola*, requires almost two thirds of all synthetic fungicides sprayed in the European Union with adverse effects on the environment^3^.

*Plasmopara viticola* (Berk. and Curt.) Berl. and de Toni belongs to the group of oomycetes (Order: *Peronosporales*, Family: *Peronosporaceae*) that comprises the most devastating plant pathogens such as *Phytophthora infestans* responsible for the Irish potato famine in the 19th century^4^. Unlike the hemibiotroph *Phytophthora* species, *P*. *viticola* is an obligate biotroph and therefore relies entirely on grape as a host to complete its life cycle. *P*. *viticola* is endemic in North America where the *Vitis* species such as *V*. *riparia* are naturally resistant, likely as a consequence of a long co-evolutionary process^5^. Conversely, *P*. *viticola* was introduced unintentionally in Europe in the 1870s and immediately spread on *V*. *vinifera* cultures causing pandemics all over Europe in the following decades^6^. Soon after, the extensive use of copper formulations known as the “Bordeaux mixture” restricted the disease spread and later on paved the path to excessive usage of synthetic agrochemicals. As a consequence *P*. *viticola* became recently resistant to many fungicides^7^. Therefore, research on grape resistance mechanisms and the development of alternative strategies to control *P*. *viticola* infections are urgently needed to control the environmental burden of grapevine cultures.

Molecular mechanisms occurring during the compatible interaction between *P*. *viticola* and *V*. *vinifera* are largely unknown. Most of our knowledge about oomycete pathogenicity factors derives from studies focused on *Phytophthora* species^8^. During infection oomycetes secrete cytoplasmic and apoplastic effector proteins that usually suppress plant immunity by triggering susceptibility (effector-triggered susceptibility, ETS)^9^. Certain oomycete effectors with a special protein motif RxLR are recognized by the plant resistance genes and this interaction triggers immunity (effector-triggered immunity, ETI) resulting in localized cell death or hypersensitive response (HR)^10^. RxLR effectors are found abundantly in all sequenced oomycete genomes and are rapidly evolving^11^. They even acquired novel functions such as suppressing plant RNA silencing mechanisms^12^. Their role during the grape downy mildew infection is unclear and their identification requires multi-omics approaches. To this end, we sequenced the DNA extracted from a *P*. *viticola* strain isolated in Northern Italy and present its assembled draft genome. By using comparative genomics we discovered the missing metabolic feature in the *P*. *viticola* genome that could explain its biotrophic mode of life. We complemented our genome sequencing efforts with genome-wide differential gene expression analyses during the infection process and we identified a protein effector of the RxLR type triggering ETI in the resistant grapevine *V*. *riparia*. Small RNA sequencing (sRNA-Seq) combined to a genome-wide degradome study^13^ revealed a comprehensive interaction network of small RNAs (sRNAs) that target genes during infection allowing us to uncover a potential bi-directional RNA silencing strategy between the pathogen and its host despite species barriers.

## Results

### Nuclear and mitochondrial genome assemblies, gene annotation and phylogenetic analysis

Assembling the draft genome for *P*. *viticola* was a prerequisite for gene and sRNA expression profiling studies during the infection process of *V*. *vinifera*. We therefore sequenced the genome of a downy mildew strain, named ‘PvitFEM01’, isolated from a local vineyard in the Trentino region in Italy. DNA extracted from asexual sporangia from *in vitro* infected plants was used to construct Illumina paired-end libraries (2 × 100 bp) and further sequenced. Although *P*. *viticola* genetic material was extracted under sterile conditions, potential contamination with DNA originating from other sources such as the host (grapevine), potential grape endophytic or epiphytic bacteria were addressed. 83 million reads remained for assembly, after filtering against *V*. *vinifera* and bacterial sequences (Supplementary Fig. S2 and Supplementary note). In total, 57,890 scaffolds were obtained corresponding to a N50 of 4,645 bp (Table 1, Supplementary Figs S1–S6 and Supplementary note). The total length of the assembly was 83.54 Mb corresponding to 74% of the genome size previously determined by Feulgen staining^14^. Contigs corresponding to the mitochondrial genome were also identified and provided the first *P*. *viticola* mitochondrial reconstruction estimated to be ca. 39.2 kb (Supplementary Fig. S7). *P*. *viticola* mitochondrial proteins identity ranged from 80 to 97% with respect to their *P*. *infestans* counterparts. A a single amino acid change from Glycine to Alanine at position 143 (G143A) in the mitochondrial apocytochrome b protein suggests that ‘PvitFEM01’ is resistant to Quinone outside Inhibitors (QoI) fungicides (Supplementary Fig. S8). Several polymorphic sites were identified on the *P*. *viticola* mitochondrial genome suggesting that our draft assembly might condense several haplotypes (Supplementary Fig. S9).

**Table 1:** Characteristics of the three available *P*. *viticola* genomes.

	PvitFEM01	INRA-PV221^a^	JL-7–2^b^
**Natural** ***Vitis*** **host**	*V*. *vinifera*	*V*. *vinifera*	*V*. *riparia*
**Geographical origin**	Italy	France	China
**Genome assembly**			
Sequencing platform	Illumina	Illumina	Illumina + PacBio
Number of scaffolds	57,890	1,883	2,165
N50^c^ (kb)	4.645	180.6	172.3
Assembly size (Mb)	**83**.**54**	**74**.**74**	**101**.**3**
**Gene annotation**			
Protein coding genes (predicted)	38,298	nd	17,014
Validated transcripts (RNA-Seq)	18,335	nd	11,670
Orthologous groups in Oomycetes^d^	6,552	nd	nd
**Genome completeness**			
Oomycete core genes present^e^	81%	na	na
CEGMA/BUSCO^f^	na/87.2%	95%/na	97%/90%

^a^Data retrieved from Dussert *et al*.^21^. nd: not determined in the study. na: not applicable.

^b^Data retrieved from Yin *et al*.^22^. nd: not determined in the study. na: not applicable.

^c^N50 is defined as the scaffold size such that 50% of the assembled nucleotides resides on contigs at least this length.

^d^Number of orthologous groups found in oomycetes with sequenced genomes and containing at least one protein from *P*. *viticola*.

^e^In total, 1,299 genes shared by all oomycetes were found, of which 1,054 were present in *P*. *viticola*.

^f^Indicated CEGMA and BUSCO numbers include the counting of partial matches.

**Figure 1:**
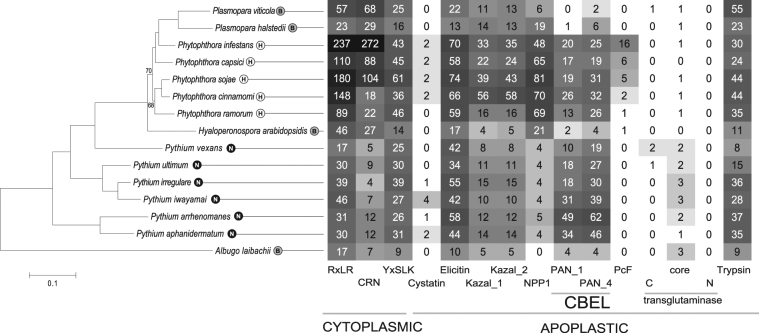
Phylogenetic relationship between *Plasmopara viticola* and other oomycetes and abundance of their effectors. The maximum-likelihood phylogenetic tree was built using 312 concatenated proteins selected from single copy genes belonging to the oomycete core genome. The abundance of each class of cytoplasmic and apoplastic effectors effector in biotroph (B), hemibiotroph (H) or necrotroph (N) oomycete species is indicated by a number and a color code. Out of the 87 YxSLK effectors identified in our study only 25 contained a signal peptide and reported in this figure. Darker colors indicate higher abundance.

In total, 38,298 genes were predicted *in silico* of which 33,982 were annotated using gene ontology terms (Supplementary Table S1). Sequencing of RNA transcripts (RNA-Seq) extracted from *P*. *viticola* sporangia and infected leaf material collected at five time points confirmed that 18,335 of these annotated genes were expressed (Table 1, Supplementary Tables S2 and S3, Supplementary Fig. S10 and Supplementary note). Additionally, 320 tRNA-encoding genes encoding representing all 20 isotype classes, as well as the 28 S and 5 S ribosomal genes (rRNAs) were identified in the *P*. *viticola* genome (Supplementary Tables S4 to S6 and Supplementary note). The 28 S rRNA is most similar to the one reported for *P*. *viticola* f. sp. *vinifera*^5^ indicating that ‘PvitFEM01’ isolate belongs to lineage C which is virulent on *V*. *vinifera* and some grape hybrids, but not on *V*. *riparia* (Supplementary Table S5). To assess the degree of completeness of our draft genome, we defined the oomycete core genome consisting of 1,299 genes of which 81% are also present in *P*. *viticola* (Table 1, Supplementary Tables S7 and S8, Supplementary Figs S11–S13 and Supplementary note). This number was corroborated by a BUSCO^15^ analysis that retrieved a completeness of 87.2% when 303 conserved eukaryotic proteins were considered (Supplementary Table S9 and Supplementary note). The phylogenetic relationship between *P*. *viticola* and sequenced oomycetes was established using 312 single-copy core genes. *P*. *viticola* is placed, together with *Plasmopara halstedii*, within the *Phytophthora* clade with 100% support (Fig. 1, Supplementary Figs S14 and S15 and Supplementary note). Branches in the *Plasmopara* lineage are longer than the average branch lengths of *Phytophthora* species. The phylogenetic analysis suggests that *P*. *viticola* derives from a necrotrophic *Phytophthora*-like ancestor.

### *P*. *viticola* secretes a RxLR effector triggering an immune response in *Vitis riparia* but not in *Vitis vinifera*

Oomycetes secrete various types of cytoplasmic effectors to infect their hosts. These effectors were mainly studied in *Phytophthora* species. We identified that *P*. *viticola* encodes 57 RxLR and 68 crinkler (CRN) proteins. In comparison, other oomycetes belonging to the Pythium genus have only five to twelve CRN proteins, suggesting an expansion of this effector type in the ancestor that gave rise to the *Hyaloperonospora*, *Phytophthora* and *Plasmopara* lineages (Fig. 1, Supplementary Tables S10–S14, Supplementary Figs S16–S21 and Supplementary note). The number of genes encoding the recently discovered YxSLK effectors^16^ is high in *P*. *viticola* and *P*. *sojae* with, respectively 87 (25 with a signal peptide) and 125 genes, suggesting that they have a special function in these two pathogens (Fig. 1, Supplementary Table S15, Supplementary Fig. S22 and Supplementary note). Most apoplastic effectors previously identified in oomycete genomes are encoded by the *P*. *viticola* genome with the largest family being the endo-β-1,3-glucanases inhibitors with a trypsin domain. This family includes 55 members and is the largest among all available oomycete genomes. In contrast to *Phytophthora* and *Pythium* species, *P*. *viticola* has only 22 elicitins. NPP1 proteins inducing necrosis likely underwent expansion in the *Phytophthora* lineage whereas only six and 19 members were found in *P*. *viticola* and *P*. *halstedii*, respectively, which may suggest extensive gene loss as an adaptation to biotrophy. CBEL (cellulose binding elicitor lectin) and PcF (*Phytophthora cactorum*-Fragaria toxin family) from *P*. *infestans* are known to trigger programmed cell death in its host^17,18^. Their gene families are either strongly reduced or completely absent in *Plasmopara* species (Fig. 1, Supplementary Table S16 and Supplementary note). In conclusion, except the glucanase inhibitor family (trypsin), all known apoplastic effectors underwent contraction in the *Plasmopara* lineage from a *Phytophthora*-like ancestor.

**Figure 2:**
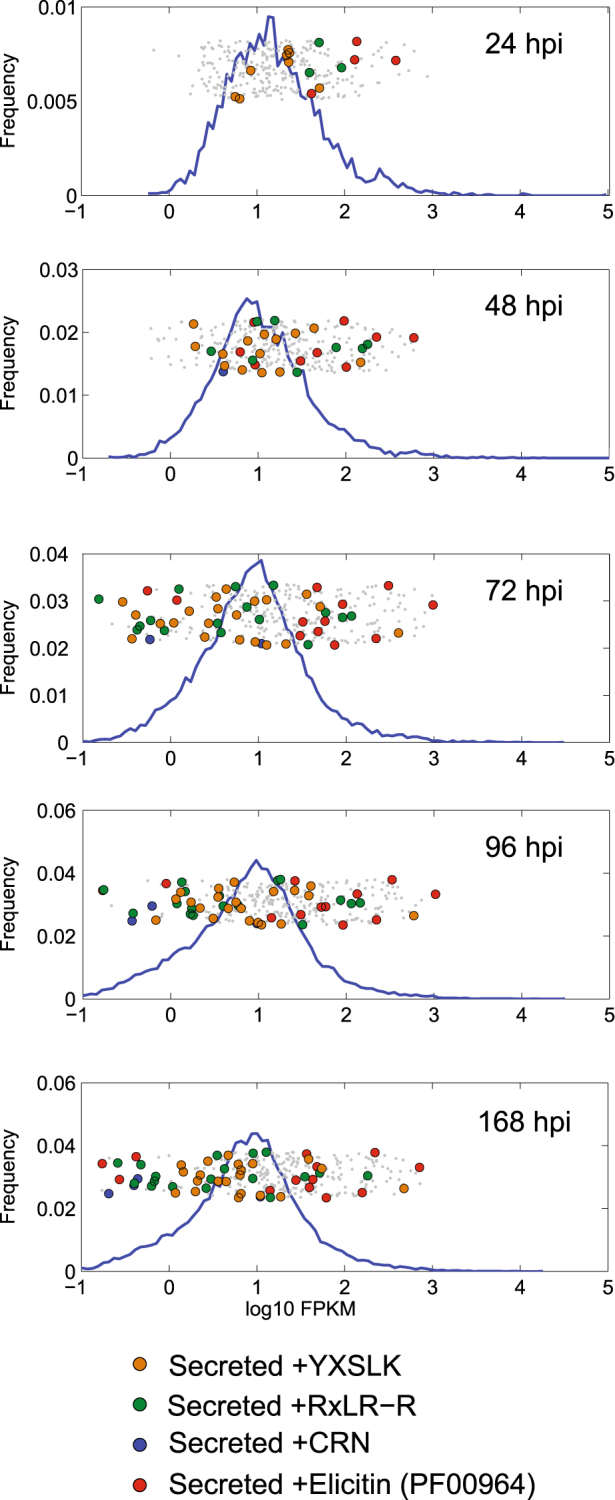
Distribution of genome-wide expression levels of *Plasmopara viticola* genes and effectors during the infection time course. Histograms represent the distribution of log10 FPKM values for all *P*. viticola genes at different hours post-infection (hpi). The values on the y-axis are counts. The gray dots represent all genes classified as cytoplasmic or apoplastic effectors. The colored dots indicate the different classes of effector proteins also possessing a signal peptide for secretion.

**Figure 3:**
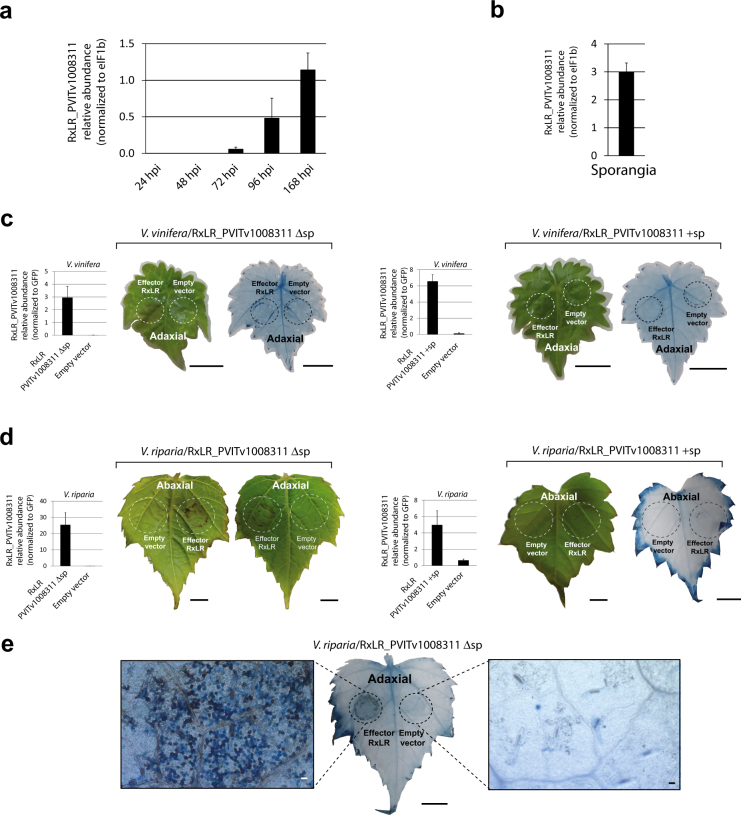
*Agrobacterium*-mediated infiltration assays of RxLR_PVITv1008311 in *Vitis vinifera* and *V*. *riparia*. The relative RxLR PVITv1008311 expression levels were measured during the infection of *V*. *vinifera* (**a**) at different hours post-infection (hpi), in sporangia (**b**) and one week after infiltration with the RxLR effector PVITv1008311 or the empty vector in *V*. *vinifera* (**c**) and *V*. *riparia* (**d**). The error bars of the relative abundance of the transcript normalized to the *P*. *viticola* elongation factor eIF1b in each panel represent the standard deviation of three independent plants or experiments. The necrosis visible on *V*. *riparia* leaf in panel (d) is due to dead cells stained in dark blue after trypan blue staining (**e**). The scale bar represents 5 mm in panels c, d, e and 20 μm in the microscopic pictures in (**e**).

Transcriptional profiling (RNA-Seq) of *P*. *viticola* genes during the compatible interaction with *V*. *vinifera* revealed differential expression of several annexins, glutathione S-transferases and one glutamic acid decarboxylase (GAD) involved in the production of the non-proteinogenic amino acid γ-Aminobutyric acid (GABA). Furthermore, proteins involved in the hydrolysis of plant material were the first set of apoplastic effectors expressed upon infection (Supplementary Tables S17 and S18). Elicitins, RxLR and YxSLK effectors were among the most highly expressed secreted proteins at later time points (Fig. 2, Supplementary Tables S19–S23, Supplementary Figs S23–S25 and Supplementary note). While the majority of RxLR, Crinkler and YxSLK effectors showed fluctuating levels during infection, the RxLR gene PVITv1008311 was expressed with FPKM values increasing to 9.8 and 17.8 at 96 and 168 hours post-infection (hpi), respectively (Supplementary Table S23). The expression of PVITv1008311 was also measured by qRT-PCR during infection and in sporangia corroborating the RNA-Seq data obtained in our study (Fig. 3a and b). To verify the role of RxLR_PVITv1008311 in pathogenicity, we expressed the coding sequence constructs both with (+sp) or without (Δsp) signal peptide *in planta* by infiltrating sterile grape leaves grown *in vitro*. There were no noticeable symptoms visible on *V*. *vinifera* leaves even two weeks after infiltration despite strong expression of the effector *in planta* (Fig. 3c). In contrast, when the same vector constructs were infiltrated in the resistant grape *V*. *riparia* a strong necrotic phenotype was observed around the site of infiltration only in leaves infiltrated with RxLR_PVITv1008311Δsp but not with RxLR_PVITv1008311 +sp (Fig. 3d). Trypan blue staining revealed that this halo corresponds to cells that underwent cell death typical of a hypersensitive response (Fig. 3e). Several additional elicitors with or without the signal peptide were tested by infiltrating *V*. *vinifera* and *V*. *riparia* leaves but the response remained asymptomatic (Supplementary Table S24). Interestingly, the sequence of RxLR_PVITv1008311 was found intact in the European isolate INRA-PV221 but completely fragmented in the Chinese isolate JL-7–2 originally isolated from *V riparia* (Supplementary Table S25). Collectively, the fact that RxLR_PVITv1008311Δsp elicits a hypersensitive response in *V*. *riparia* but not in *V*. *vinifera* indicates that grapevine cultivars grown in Europe lost or perhaps did not acquire yet the recognition of *P*. *viticola* effectors to initiate a proper immune response.

### Nitrogen metabolism and γ-Aminobutyric acid (GABA) catabolism are missing in *P*. *viticola*

**Figure 4:**
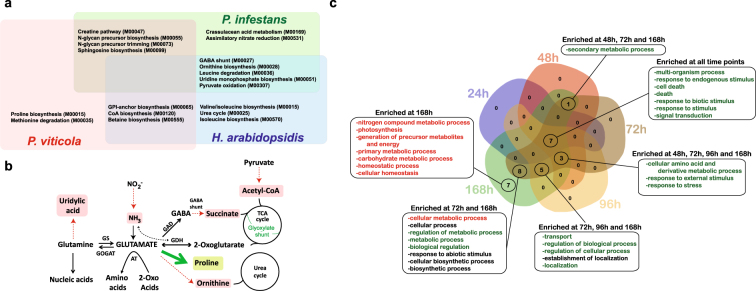
Metabolic pathways missing in *Plasmopara viticola* and those induced in grapevine during infection. The Venn diagram shows the metabolic pathways specific to *P*. *viticola* or shared with two other oomycetes, *Phytophthora infestans* and *Hyaloperonospora arabidopsidis*. The KEGG module number M is indicated in brackets (**a**). A summary of the pathways missing in *P*. *viticola* indicated in red. The proline biosynthesis pathway indicated in green is found only in *P*. *viticola* but not in the two other oomycetes *P*. *infestans* and *H*. *arabidopsidis*. (AT: Amino transferase, GAD: glutamic acid decarboxylase, GDH: glutamate dehydrogenase, GOGAT: glutamine oxoglutarate aminotransferase, GS: glutamine synthetase, TCA cycle: tricarboxylic acid cycle) (**b**). A Venn diagram representing the gene ontology terms of *V*. *vinifera* genes enriched at each time point during infection in grapevine. The metabolic pathways indicated in red indicate genes induced whereas those in green refers to genes repressed (**c**).

To better understand *P*. *viticola* biotrophic mode of nutrition we identified the metabolic modules that are either completely missing from its genome or differ significantly from the biotroph *H*. *arabidopsidis* and the hemibiotroph *P*. *infestans*. Similar to the other obligate biotroph *H*. *arabidopsidis*^19^, *P*. *viticola* lacks both nitrite and sulfite reductases (Supplementary Figs S26 and S27). However, unlike other obligate biotrophs, *P*. *viticola* lacks several enzymes involved in the conversion of L-glutamate to either succinate (KEGG module M00027) or to L-ornithine (M00028). The pathways leading to conversion of leucine to acetoacetate and Acetyl-CoA (M00036), of L-glutamine to uridylic acid (M00051), and of pyruvate to acetyl-CoA (M00307) are also incomplete in *P*. *viticola* (Supplementary Fig. S28 and Supplementary Table S26). However, in contrast to the two other oomycetes, the *P*. *viticola* genome encodes all genes necessary to convert L-glutamate to L-proline (M00015) and to degrade L-methionine to L-cystathionine (M00035) (Supplementary Fig. S29). Furthermore, the two biotrophs *H*. *arabidopsidis* and *P*. *viticola* seem to have lost some enzymes required for the biosynthesis of glycosylphosphatidylinositol (GPI)-anchor (M00065), Coenzyme A (M00120), and betaine (M00555). In contrast, the pathways leading to biosynthesis of creatine (M00047), sphingosine (M00099) and N-glycan precursors (M00055, M00073) are conserved in *P*. *viticola* and *P*. *infestans* but are lost in *H*. *arabidopsidis* (Fig. 4a and Supplementary Table S26). The nitrogen metabolism, sulphur assimilation, GABA shunt, ornithine biosynthesis and uridine monophosphate biosynthesis pathways seem also incomplete in the two other *P*. *viticola* isolates INRA-PV221 and JL-7–2 (Supplementary Table S27) suggesting that glutamate metabolism and its connection with citric acid (tricarboxylic acid; TCA) and urea cycles, as well as uridylic acid biosynthesis and GABA catabolism, might be impaired in *P*. *viticola* (Fig. 4b). Given the role of glutamate in amino acid metabolism and nitrogen utilization, *P*. *viticola* infection could have an important impact on grapevine metabolism during the compatible infection. To verify this hypothesis, we performed a differential gene expression analysis using RNA-Seq at multiple time points during infection of grapevine by ‘PvitFEM01’ and characterized the gene sets by functional enrichment analysis using GO annotations (Supplementary Table S28). Interestingly, grapevine genes involved in secondary metabolic processes, cellular amino acid metabolism and derivative metabolic processes were significantly repressed in infected tissues starting at 48 hpi. The genes involved in nitrogen compound metabolic processes and homeostasis start to be expressed later during the infection process at 168 hpi which might suggest that downy mildew stimulates the production of growth nutrients from its host while avoiding triggering cell death (Fig. 4c).

### A bidirectional cross-species sRNA-mediated gene regulation during the compatible interaction

Plants defense strategies against viral and fungal pathogens rely largely on RNA silencing and the action of sRNAs^20^. To explore this mechanism of defense during *P*. *viticola*-*V*. *vinifera* interaction, we sequenced sRNAs from both healthy and infected grapevine plants at 24, 48, 72, 96 and 168 hpi. The sRNA profile of *V*. *vinifera* showed enrichment in 21- and 24-nt sRNAs typical of plants, while *P*. *viticola* has an almost equal abundance of 21- and 25-nt sRNA classes that are also abundantly expressed in sporangia (Supplementary Fig. S30). In total, two dicer-like proteins (DCLs), two argonaute proteins (AGOs) and one RNA-dependent RNA polymerase (RDR), as well as enzymes known to regulate epigenetic mechanisms were identified confirming the existence a *bona fide* RNA silencing machinery in *P*. *viticola* that is active during its life cycle inside the grapevine host (Supplementary Table S29). Among these proteins two DCL-like enzymes, defined by the presence of a Dicer dimerization domain were found, suggesting that one of them could be dedicated to process the 25-nt sRNA class. *P*. *viticola* sRNAs of 21- to 22-nt length were generated from 592 transcripts coding mainly for transporters, transcription factors, methyltransferases, metabolic genes and elicitins. In contrast, the 25- to 26-nt sRNA class derives almost exclusively from genes related to transposition (Supplementary Fig. S31). The 21- to 22-nt sRNAs deriving form coding genes were mapping in sense and antisense orientation suggesting that a dsRNA intermediate is synthesized, most probably, by the unique RDR found in the *P*. *viticola* genome (Supplementary Fig. S32). Additionally, a total of 18 CRN (Supplementary Fig. S33), YxSLK (Supplementary Fig. S34) and RxLR (Supplementary Fig. S35a) effector genes produced a high amount of 21/22-nt short interfering RNA (siRNAs) duplexes suggesting a preponderant role in post-transcriptional regulation of these pathogenicity factor during the infection process (Supplementary Table S30). The highly structured PVITv1_T024389 RNA transcript encoded a protein with an unusual LFLAK/RxLR tandem motif and displayed a unique processing pattern among eukaryotes with 21/22-nt siRNA duplexes processed every 60- to 90-nt (Supplementary Figs S35b and S36).

**Figure 5:**
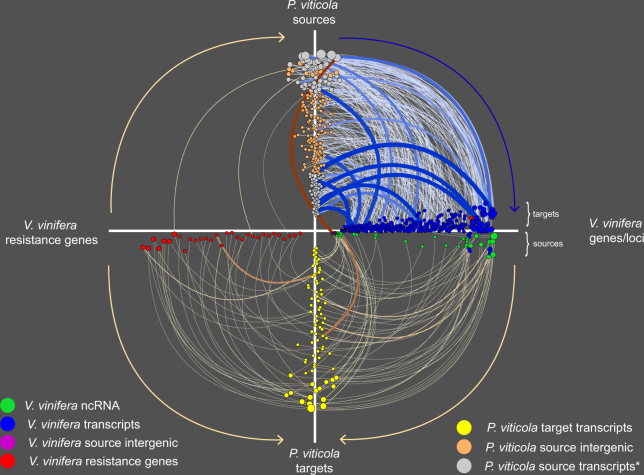
Bidirectional cross-species sRNA-mediated gene regulation during the compatible interaction. The hive plot indicates the interactions between *P*. *viticola* sRNAs originating from either intergenic (yellow dots) or protein coding genes (gray dots) and *V*. *vinifera* genes (blue dots). Reciprocally, *V*. *vinifera* sRNAs processed from either noncoding RNA (green dots), intergenic regions (purple dots) or resistance genes (red dots) target *P*. *viticola* transcripts (yellow dots). The thickness and color intensity of the yellow and blue lines representing the sRNA-target interactions are proportional to the log transformed p-value calculated for the number of reads from the degradome whose 5′ end corresponds (±1) to the expected sRNA-mediated cleavage site: the larger the edge, the more significant the interaction. The color code is different for regulation starting (from light to dark blue) or arriving at *P*. *viticola* (from yellow to red). The size of the dots corresponds to the number of regulations identified for a certain sRNA.

In order to address the potential regulatory role of both grapevine and *P*. *viticola* sRNAs during infection, we performed a genome-wide analysis of the RNA degradome or PARE (parallel analysis of RNA ends) of infected material and control plants. In order to support and validate our sRNA target prediction we used SeqTar with stringent parameters and retained only the highly probable sRNA-mRNA interactions based on a mismatch and binding score p-value ≤ 0.001 and a valid peak height of p-value ≤ 10^−10^. We confirmed that grapevine endogenous microRNAs (miRNAs) regulate genes important for plant growth and development in both infected and control plants (Supplementary Table S31). We also characterized the degradome of *P*. *viticola* and identified genes targeted by its endogenous sRNAs such as kinases and a vesicle-associated membrane protein VAC14 only in the degradome-Seq dataset generated from infected plants (Supplementary Table S32). Interestingly, we have identified a potential bidirectional interaction between, on one hand; the sRNAs produced by *P*. *viticola* triggering cleavage of grapevine genes and on the other hand, the sRNAs processed from grapevine transcripts and targeting the oomycete messenger RNAs (Fig. 5, Supplementary Tables S33 and S34). Small RNA duplexes processed from grape resistance genes in 21-nt increment and known as phased secondary siRNAs (phasiRNAs), were the most abundant class of grapevine sRNAs and targeted for cleavage *P*. *viticola* genes with diverse functions (Fig. 5 and Supplementary Table S34). Other sRNAs processed from grape noncoding RNAs such as the miRNA primary transcripts pri-*MIR169*, pri-*MIR171a*, pri-*MIR394c*, pri-*MIR482-like* and pri-*MIR396a* as well as the trans-acting siRNA precursor *TAS3* also trigger cleavage of various *P*. *viticola* transcripts. Reciprocally, *P*. *viticola* sRNAs, including those deriving from the CRN gene PVITv1_T024389, target *V*. *vinifera* genes for cleavage at multiple sites (Fig. 5 and Supplementary Table S33). Our results suggest that similarly to mechanisms described for protein-coding gene effectors, noncoding small RNAs potentitally mediate interference between the pathogen and its host in a bi-directional manner in a way not previously known.

## Discussion

The primary goal of this study was the identification of *P*. *viticola* pathogenicity factors involved in the infection process of grapevine. To enable the use of transcriptomic approaches we first sequenced the DNA isolated from infected plants and assembled the *P*. *viticola* genome. The genome assembly of the Italian *P*. *viticola* ‘PvitFEM01’ reached 83.54 Mb, a genome size between the Chinese isolate ‘JL-7–2’ and the French one INRA-PV221 with 101.3 Mb and 74.74 Mb, respectively^21,22^. The genome of the Chinese isolate reached a higher size of contigs due to the assembly of long reads obtained using the PacBio single-molecule sequencing technology whereas this work relied solely on paired-end Illumina sequencing technology. Nevertheless, the genome obtained in our study reached a completeness that was sufficient enough to identify and annotate 18,335 genes with homology to oomycete proteins for which expression was experimentally verified by RNA-Seq data.

We did not find a massive gene loss that could explain obligate biotrophy in *P*. *viticola* as reported for *H*. *arabidopsidis*^19^. On the contrary, our data suggest that *P*. *viticola* contain more RxLR, CRN and YxSLK effector genes compared to other biotrophs. The phylogenetic analysis showed that the two downy mildew species *P*. *viticola* and *P*. *halstedii* share the same clade and are evolutionary close to the *Phytophtora* species^23^. In contrast, *H*. *arabidopsidis* was placed in a sister group therefore confirming recent findings that downy mildews are not monophyletic^24^. Our work contribute to the revision of downy mildews phylogenesis and confirms that *Plasmopara* species derived from a *Phytophthora*-like ancestor that switched to obligate biotrophy, lost its necrotrophic abilities and became specialized for a defined host species. We provide additional genome sequences and gene annotation that will enable a thorough comparative genomic approach and will help identifying the molecular mechanisms that dictate obligate biotrophy.

The sequenced *P*. *viticola* isolate ‘PvitFEM01’ is virulent on *V*. *vinifera* but not on the wild North American species *V*. *riparia*. Our results also show that this isolate is resistant to Quinone outside Inhibitors (QoI) fungicides since a single amino acid change from Glycine to Alanine at position 143 (G143A) was found in the mitochondrial apocytochrome b protein similarly to other European downy mildew isolates highly resistant to QoI^7^. Sequencing of the 28 S rRNA showed that *P*. *viticola* ‘PvitFEM01’ belongs to the cryptic lineage C and therefore evolved on *V*. *vinifera* after introduction of the pathogen in Europe^5^. Taking this into account, we expected that *P*. *viticola* would express effector genes triggering no defense response in *V*. *vinifera*. *Agrobacterium*-mediated infiltration of effectors in *V*. *vinifera* leaves resulted in no visible phenotype confirming that the isolate ‘PvitFEM01’ evolved a stealth infection strategy similarly to other obligate biotrophs^19^. Remarkably, expression of RxLR_PVITv1008311 in leaves of the resistant cultivar *V*. *riparia* triggered a hypersensitive response, indicating that this RxLR effector is one of the key evolutionary players in the perpetual arm race between *P*. *viticola* and its grapevine host. To successfully infect *V*. *vinifera*, *P*. *viticola* encodes RxLR effectors with different properties according to the *Vitis* species with which they have co-evolved. RxLR effectors can suppress plant immunity when *P*. *viticola* infects wild *Vitis* species such as *V*. *amurensis*^25^ or, as in the case of RxLR_PVITv1008311, they have most likely lost the potential to trigger cell death when infecting the domesticated *V*. *vinifera*. However, the effector is still recognized by the immune system of *V*. *riparia* most probably for the presence in the host genome of the resistance gene recognizing RxLR_PVITv1008311. Our finding opens therefore a new route in grapevine breeding programs since RxLR_PVITv1008311 can be used efficiently in effector-based high-throughput *in planta* expression assays^26^. This will help to accelerate the identification of new *V*. *vinifera* hybrids or varieties resistant to *P*. *viticola* hence reducing the use and the release in the environment of toxic fungicides and chemicals.

Several studies attempted to explain obligate biotrophy by a loss of certain metabolic pathways^27^. Similarly to other obligate biotrophs *P*. *viticola* lost the nitrate and nitrite reductase enzymes suggesting a total dependence on the grape host for acquiring nitrogen in its reduced form^19,23^. Additionally, our study reveals that not only the Italian *P*. *viticola* PvitFEM01 but also the French isolate INRA-PV221^21^ and the Chinese one JL-7-2^22,28^ lost the genes encoding many enzymes necessary for the conversion of glutamine to uridylic acid and of glutamate to succinate and ornithine. The two latter molecules bridge glutamate metabolism to the TCA and urea cycles. The reconstruction of metabolic pathways by gene annotation provides the evidence for a functional glutamate metabolism in *P*. *viticola*, however, relying on the use of ammonia from the host grapevine. This seems a general feature of obligate biotroph pathogens. However, our study also reveals that enzymes involved in amino acid metabolism from glutamate are conserved in *P*. *viticola* suggesting that this biotroph does not rely on his host for synthesizing this fundamental amino acid. On the contrary, the GABA shunt pathway was impaired suggesting that succinate is most likely synthesized through the TCA/glyoxylate shunt, but not from GABA in *P*. *viticola*. This non-proteinogenic amino acid is synthesized from glutamate by the action of the glutamic acid decarboxylase (GAD) enzyme that is strongly expressed in *P*. *viticola* during the course of infection. Taken together our data suggest that enzymatic reactions leading to the production of GABA are strongly activated in *P*. *viticola* during infection, however, those associated with GABA catabolism are impaired. This implies an important increase of GABA levels during infection by *P*. *viticola* that will not be degraded. Interestingly, GABA reduces H_2_O_2_ levels by up-regulating the expression of the catalase *VvCAT2* in grapevine^29^. Whether *P*. *viticola* evolved a strategy to increase GABA levels during infection in order to suppress oxidative stress in its host remains to be verified.

Besides the potential role of *P*. *viticola* protein-coding genes in the regulation of the infection process, our study unveiled a potential bidirectional gene regulation mediated by noncoding RNAs between *P*. *viticola* and its host. Necrotrophic fungi evolved a sRNA-mediated silencing of their host genes to rapidly suppress immunity and successfully infect and destroy the plant tissues^30^. This post-transcriptional regulation mechanism occurs most probably by a direct uptake of the sRNAs into the cells in the vicinity of the infection site^31^. In contrast, biotrophic oomycetes must keep their host cells alive until sporulation occurs and exchange of biological material is intensive during the compatible interaction. Taking into account the computational output obtained using SeqTar with stringent parameters, the large number of sRNA-mediated cleavages occurring only in infected tissue but not in control plants are highly probable. This mechanism would implicate an important shuffling of low molecular weight RNA between *P*. *Viticola* and its host. This bidirectional exchange could occur either via the haustorium or through simple diffusion between cells in contact. However, additional experimental work is needed to confirm and verify if the pairing of the sRNAs to their cognate target genes occurs randomly in the cytoplasm or if it is evolving towards a type of gene regulation involving specialized protein complexes. It is not excluded that this sRNA-mediated gene regulation is still evolving given that the *P*. *viticola*-*V*. *vinifera* pathosystem appeared only about 135 years ago^32^. The winner of this evolving arms race between *P*. *viticola* and *V*. *vinifera* is difficult to predict.

In conclusion, our work provides new insights on the molecular mechanisms governing pathogenicity of grapevine downy mildew and lays the foundation for future work aiming to develop alternatives to the heavy use of chemical treatments. Based on the results of our work, we propose the development of RNAi-based techniques such as host induced gene silencing (HIGS)^33^ or spray-induced gene silencing (SIGS)^34^ to knockdown *P*. *viticola* pathogenicity genes as an environmental-friendly alternative of crop protection.

## Methods

Methods and any associated references are available in the online version of the paper.

### Accession codes

The raw data corresponding to the genome, RNA-Seq, sRNA-Seq and degradome-Seq sequences used in this study has been deposited at GenBank under the project accession PRJNA380033. The mitochondrial genome described in this study has been deposited to GenBank under the accession number KY885002.

## Online Methods

### Plant material, growth conditions, inoculation and infiltration

*V*. *Vinifera* susceptible cultivars Pinot Noir cv. ENTAV115, the near-homozygous Pinot Noir 40024 and Sultanina as well as the resistant *V*. *riparia* used in this study were cultivated *in vitro* in glass tubes on half-strength Murashige Skoog (MS) medium containing 0.6 mg/l thiamine, 100 mg/l myo-inositol, 30 g/l sucrose and 6 g/l agar. The plants were grown at 24 °C under 16 h of light/8 h of dark with an illumination of 70 μmol m^−2^s^−1^ light. The isolation from the field of *P*. *viticola* “PvitFEM01”, the inoculum preparation and the sterile infection of *in vitro* grapevine plants were performed as described in Lenzi *et al*.^35^. The RxLR and CRN effectors were cloned using the primers described in supplementary Table S35. The *Agrobacterium* infiltration assays and the gene expression studies by qRT-PCR were performed as described in the supplementary note.

### Genome sequencing and gene annotation

*P*. *viticola* is a biotroph and therefore cannot be cultivated and grown on synthetic culture medium. The starting DNA for library preparation was isolated from a mix of sporangia, sporangiophores and mycelia emerging from infected grapevine cv. Pinot Noir ENTAV115 grown *in vitro*. *Plasmopara viticola* genomic DNA was extracted using the method described in Si-Ammour *et al*.^36^. The MicroPlex Library Preparation Kit (Diagenode, www.diagenode.com) was used to build the Illumina library using 60 ng of *P*. *viticola* genomic DNA and following the manufacturer’s recommendations. The library was sequenced using a HiSeq. 2500 Illumina platform (Illumina, www.illumina.com) at Fasteris (www.fasteris.ch). DNA fragments were sequenced from both ends to generate 2 × 100 bp paired-end reads. All reads mapping on the grapevine genome cv. PN40024^37^ and contaminating bacterial sequences were filtered and eliminated to produce a preliminary assembly using Abyss^38^. Several k-mer lengths were tested and the best N50 (11kbp) value was obtained for a k-mer of 60 nucleotides. To remove sequences not belonging to the *P*. *viticola* genome, we followed the flowchart indicated in Supplementary Fig. S2 and explained in details in the supplementary note. The final assembly was then selected among the outputs of Ray^39^ and Abyss^38^ ran with different k-mer lengths as described in details in the supplementary note. The *P*. *viticola* mitochondrial scaffolds/genes from the Ray assembly were identified on the basis of similarities with *P*. *infestans* mitochondrial sequences^40^. All expected mitochondrial ORFs were found in scaffolds that were subsequently manually assembled. Gene finding and gene training was performed using Augustus^41^, GlimmerHMM^42^ and GeneID^43^. Gene predictions were supported by RNA-Seq data and the genes named as described in the supplementary note. Transfer RNA genes were identified using tRNA-scanSE^44^ and ribosomal genes annotated using RNAmmer^45^. The degree of completeness of the assembly was estimated by comparing our final assembly with available data such as the genome size determined by Feulgen staining^14^, the genome size of *P*. *halstedii*, sequences of *P*. *viticola* available in different databases, a BUSCO^15^ analysis and our comparative genomics study, as described in the supplementary note.

### Comparative genomics and phylogenetic analyses

The ortholog groups from the 15 oomycete species including *P*. *viticola* were used to identify the oomycete core genome. Pairs of genomes were compared using Inparanoid^46^ and the outputs were integrated using QuickParanoid (http://pl.postech.ac.kr/QuickParanoid/). Phylogenetic analyses of the oomycete dataset were performed using a concatenation of 312 core ortholog proteins containing a single copy per genome and aligned using MAFFT^47^. The alignment was further filtered with Gblocks^48^. Phylogenetic trees were built using phyml^49^ and raxml^50^. The RxLR, RxLR-like, CRN and YxSLK effectors were identified as described in the supplementary note. Apoplastic effectors were identified by scanning protein sequences with the corresponding HMM models from Pfam^51^ (http://pfam.xfam.org/).

### RNA-Seq, sRNA-Seq and degradome-Seq

Infections of *V*. *vinifera* cv. ENTAV115 *in vitro* plants with *P*. *viticola* (isolate ‘PvitFEM01’) were performed as described in Lenzi *et al*.^35^. Both infected and non-infected plants were harvested at five time points (0, 24, 48, 72, 96 and 168 hours post-infection, hpi) in duplicates with 20–25 plants in each replicate. The replicates are from independent experiments. Sporangia of *P*. *viticola* were collected from infected material at late time points (96 and 168 hpi). Total RNA was extracted using the Spectrum plant total RNA (www.sigmaaldrich.com) and the small RNA fraction recovered from the flowthrough following the manufacturer’s instructions. The RNA-Seq and sRNA-Seq libraries were built using the TruSeq RNA and TruSeq Small RNA Library Prep kits (www.illumina.com), respectively, following the manufacturer’s protocol. The degradome-Seq libraries were constructed from RNA extracted from pooled material of infected and non-infected plants using the parallel analysis of RNA ends (PARE) protocol as described by German *et al*.^13^ by Vertis Biotechnologie AG (www.vertis-biotech.com). The RNA-seq and degradome-Seq libraries were sequenced on a HiSeq. 2500 platform (www.illumina.com) at the LaBSSAH facility (www.labssah.eu) and Vertis Biotechnologie AG (www.vertis-biotech.com), respectively. The RNA-seq libraries were processed as described in the supplementary note. Differential gene expression analysis of the *P*. *viticola* and *V*. *vinifera* transcriptomes were performed by using the Cufflinks pipeline as described in the supplementary note^52,53^. Targets cleaved by sRNAs were predicted using SeqTar^54^ and by combining different sets of sRNAs and transcriptomes as described in Šurbanovski *et al*.^55^. A set of sRNAs of 21nt that mapped perfectly on *P*. *viticola* genome and a set of *V*. *vinifera* sRNAs of 21 and 22nt were used to search for mRNA targets in the *P*. *viticola* transcriptome from this study and to search for targets in grapevine gene sequences retrieved from Genoscope (www.genoscope.cns.fr) and CRIBI (http://genomes.cribi.unipd.it/). All SeqTar analyses were filtered using a mismatch and binding score p-value ≤ 0.001 and a valid peak height p-value ≤ 10^−10^.

## Electronic supplementary material

Supplementary information

Supplementary Table S1

Supplementary Table S18

Supplementary Table S23

Supplementary Table S26

Supplementary Table S28

Supplementary Table S31

Supplementary Table S32

Supplementary Table S33

Supplementary Table S34
