# Supplementary material for: A multi-omics study of the grapevine-downy mildew (Plasmopara viticola) pathosystem unveils a complex protein coding- and noncoding-based arms race during infection
Source: Sci Rep. 2018 Jan 15;8:757. doi: 10.1038/s41598-018-19158-8 (PMC5768699; doi:10.1038/s41598-018-19158-8)
Supplement: Supplementary file 1 — Supplementary information [file 41598_2018_19158_MOESM1_ESM.pdf]

A multi-omics study of the grapevine-downy mildew (*Plasmopara viticola*) pathosystem unveils a complex protein coding- and noncoding-based arms race during infection.

Matteo Brilli[1,2,], Elisa Asquini[1], Mirko Moser[1], Pierluigi Bianchedi[1], Michele Perazzolli[1], Azeddine Si-Ammour[1, *]

[1] Fondazione Edmund Mach, Via Mach 1, 38010, San Michele all'Adige (TN), Italy.

[2] Present address: Department of Agronomy, Food, Natural Resources, Animals and Environment (DAFNAE), University of Padova, Agripolis, V.le dell'Università, 16, 35020, Legnaro (PD), Italy.

[*]Corresponding author: azeddine.siammour@fmach.it

	Pages
- Supplementary note	1 to14
- Supplementary figures (S1 to S36)	15 to 50
- Supplementary tables (S1 to S35)	51 to 70
- Supplementary references	71

Supplementary Note

Preliminary assembly and sequence filtering

We obtained 164,574,481 100 nt long read pairs with average insert size of 234 bp as estimated post-assembly using samtools[1]. All reads mapping on the grape genome with a maximum of three mismatches were eliminated. We then removed 0.5% of reads and the remaining were used to build a preliminary *Plasmopara viticola* assembly using ABySS[2]. Several k-mer lengths were tested and the k=60 that, produced the best N50 (11kbp), was selected. This preliminary assembly (198Mbp) still likely contained sequences not coming from *P. viticola*, as suggested by the GC content distribution (**Supplementary Fig. S1**), which is clearly bimodal and the length of the assembly, since the estimated genome size is around 100Mb. To remove sequences not belonging to the *P. viticola* genome, we focused on scaffolds longer than 1,000 nucleotides and followed the flowchart indicated in **Supplementary Fig. S2** and consisting in:

1) Genes predictions were obtained using Augustus[3] after iterative training on *bona fide* and well predicted *Plasmopara* sequences, obtaining over 60,000 gene predictions.
2) Protein sequences were searched using BLAST against the non-redundant (nr) NCBI database and taxonomy information about the hits were recorded.
3) Each scaffold was assigned to one of several taxonomic categories depending on hits obtained by blastp. For instance, a scaffold with all hits belonging to Viridiplantae was assigned to the plant class, while one with hits towards Stramenopiles only was assigned to the putative *Plasmopara* class. Chimeric scaffolds were also found and those containing regions with homology to Stramenopiles were split by hand on the basis of the coverage profile over their length and the blastp alignments (e.g. **Supplementary Fig. S3)**. A scaffold was considered chimeric when at least two non-overlapping and significant hits to different taxonomic groups were found.
4) Scaffolds with no homologies in the blastp analysis and scaffolds for which no gene predictions were retrieved using Augustus were analyzed using the same strategy as above but using blastn of the entire scaffold sequence against the nt database. The scaffolds with no homology to database sequences were tentatively assigned to the *P. viticola* assembly because they might represent sequences specific of this organism.
5) After this pipeline, we obtained a preliminary assembly containing 36,533 scaffolds larger than 1,000 bp and a total length of 110 Mb. The GC content distribution changed to unimodal, as expected for a single genome (**Supplementary Fig. S1**).

The original reads were then mapped on this preliminary assembly, likely containing most of the reads coming from *P. viticola*. Mapping reads were retained and used for a second assembly.

Final Assembly

For the final assembly, we used Ray[4] and ABySS[2] with several k-mer lengths for a total of 17 runs. We hypothesized that our *P. viticola* inoculum could be composed of a pool of different haplotypes. In such case, reads coming from variable genomic regions can easily be considered as different genomic regions by the assembler, resulting in a high artificial

redundancy of the output assembly. The magnitude of the problem depends on how the assembler will consider slightly different sequences. We studied this issue by running an analysis whereby gene finding is performed on each of the 17 assemblies with the same parameters. Gene sequences from the same assembly were then clustered by sequence homology such that clusters contained sequences sharing at least 98% identity and no more than 5% length difference. The number of clusters is then compared to the original number of predicted genes to obtain a measure of the degree of redundancy in the assembly. We observed a very different behavior of the two genome assemblers. In particular, ABySS tends to build scaffolds that are much more redundant than Ray (**Supplementary Fig. S4**) probably because of the sensitivity to small differences in the reads.

Considering that we likely have a pool of haplotypes, we focused on Ray assemblies, which are less affected by the redundancy problem. It might be that ABySS is able to reconstruct more precise sequences, but in doing so it also assembles multiple times the same genomic region from different strains, inflating the genome size and the number of genes. Among the Ray assemblies, we selected R69 based on assembly statistics. In this way we were able to assemble 57,890 scaffolds. The level of fragmentation of the assembly may be caused by the short insert size coupled with potentially long repeat regions present in the genome (**Supplementary Fig. S5**). The coverage distribution (**Supplementary Fig. S6**) turned out to be multimodal, suggesting the presence of multiple haplotypes in the starting material.

Gene finding and annotation

The *P. viticola* genome assembly was obtained in different steps since filtering of the input reads was necessary given the heterogeneous nature of the starting material. We trained parameters for the gene predictor Augustus[3] on the preliminary ABySS assembly (k=60) in the following way:

1) Unique core proteins from three *Phytophthora* species were selected following the analysis reported by Haas *et al.*[5] for a total of ~7,000 proteins.
2) These proteins were aligned to our assembly using Scipio[6].
3) Scaffold regions corresponding to the most significant alignments (N=1,572) were extracted, translated in a suitable format and used for training the Augustus gene prediction model.
4) Predicted proteins were extracted and blasted against the *P. infestans* genome. Those with highly similar hits (>80% identity) and less than 10% difference in length (N=1,546) were retained for a second round of training.
5) The previous protein set was partitioned into train (N=1,046, 585 multiexonic) and test sets. The test set was further partitioned into mono- and multi-exonic (222 and 278 sequences, respectively) to test Augustus performances separately for the two classes of genes.
6) Augustus parameters were estimated from the training set and the performances were evaluated on the test set (**Supplementary Table S2**).
7) Gene prediction was run with the estimated parameters on the entire assembly giving ~60,900 genes.

Once the final assembly was obtained, Augustus was re-trained to improve the parameters over a set of *bona fide* sequences and taking advantage of i) homologies with sequences from other oomycetes and ii) RNA-Seq data of *P. viticola* sporangia and *P. viticola* infected plants. Intron and coding sequence (CDS) hints for Augustus were generated from

proteins using Exonerate software with default parameters[7]. RNA-Seq was used to produce intron hints running the *bam2hints* script available in Augustus, after aligning RNA-Seq reads using bowtie2 with no mismatches allowed in the seed region[8].

The procedure undertaken was the following:

1) Starting from the parameters obtained on the preliminary assembly, we ran Augustus over the new assembly including intron and CDS hints from protein and RNA-Seq alignments. Gene predictions were extracted and their protein sequences were blasted against a database of 12 oomycetes (*Pythium*, *Hyaloperonospora* and *Phytophthora* species). The blast was filtered by e-value (maximum 0.0001) and on the basis of i) alignment coverage of the query by the first blast alignment (minimum 90%) and ii) a maximum 5% difference in length between the predicted protein and the first blast hit. We obtained 2,853 gene predictions for training Augustus.
2) The sequences obtained as described above were partitioned into a training (2,091 gene predictions of which 1,172 are multi-exonic) and a test (762 gene predictions of which 402 multi-exonic) set.
3) Parameters of the Augustus gene model were estimated using the above training set (performances on the test set are indicated in **Supplementary Table S2**).
4) Parameters were re-trained using the whole (train+test) dataset.
5) Augustus was run on the final assembly using hints from RNA-Seq and protein sequences as in the first step to obtain the final set of Augustus gene predictions.

GlimmerHMM[9] was trained using trainGlimmerHMM on the entire set of predictions (2,853 sequences) used for Augustus, and returned 24,641 predictions.

GeneID[10] was run exploiting available parameters for *P. infestans* (http://genome.crg.es/software/geneid/). This program returned a large fraction of very small gene predictions with respect to the others (**Supplementary Fig. S10**).

As different gene finders employ different algorithms and often rely on different gene models, we integrated three of them to improve the genome annotation (Augustus[3], GlimmerHMM[9] and geneID[10]). The final gene predictions were obtained by combining information coming from available oomycete proteins and from our own RNA-seq data.

After filtering out proteins shorter than 30 amino acids, we obtained 38,284 gene predictions corresponding to 38,298 proteins for the presence of some alternative transcripts, and that represents our draft *P. viticola* proteome. Of these, 14,792 are supported by both homology with other Oomycete proteins (20,354 supported gene predictions) and reads from RNA-seq (18,335 supported gene predictions), therefore we estimate the size of the proteome to be in line with *P. halstedii*, around 20,000 proteins. KAAS[11] and Argot2[12,13] were used together with extensive blast analysis against available databases to provide functional classification of the predicted proteins. Ortholog classification of the predicted *P. viticola* proteins was made using Inparanoid[14] followed by QuickParanoid (http://pl.postech.ac.kr/QuickParanoid/). Using this strategy we obtained the *pan-genome* phylogenetic profile matrix for 15 Oomycetes.

Merging protein coding gene predictions

The three gene prediction programs that we used are all based on different algorithms and therefore generate different predictions. Nevertheless, the overlapping between the outputs is generally high and in this situation a selection of the predicted sequences that

overlap significantly was necessary. Gene prediction mergers are available, but tend to re-estimate the parameters, while we need to select one sequence per locus, and selection has to be made on the basis of some objective quantity, preferably related to the quality of the gene prediction. For that task we implemented a strategy that takes into account (i) evolutionary information in the form of alignments with available oomycete proteins, and (ii) organism specific information in the form of alignments *de novo* transcripts built from RNA-Seq data produced in this work. The strategy used was the following and it is available as a java program upon request:

1) All predicted proteins were searched using BLAST against:
    a) a database of oomycete proteins. These alignments were used to assign to each protein an "evolutionary" score, calculated as: $S_{Evo} = \frac{(L_{ali}-g)\times L_{ali}}{L_q \times L_{fbh}}$, where L stands for length and the subscripts *ali, q* and *fbh* indicate the alignment, the query and the first BLAST hit, respectively. *g* indicates the number of gaps in the BLAST alignment. Alignments used for calculating the score are only those with e-value smaller than e^{-5}. The evolutionary score therefore approaches 0 in case of short alignment length or small coverage of the first BLAST hit, while it is 1 when the alignment of the two sequences cover their entire length. The score can be seen as a measure of how close in terms of length (and therefore also gene structure, i.e. intron/exons) is the predicted protein with respect to the most similar protein from other oomycetes.
    b) the set of *de novo* transcripts was built using Cufflinks[15]. The alignments with transcripts were used to calculate an "expression" score S_{Exp} with the same formula used for the evolutionary score.
2) The evolutionary and expression scores were integrated as $S = \frac{(1+S_{Evo})\times(1+S_{Exp})-1}{3}$, such that *S* ranges from 0 to 1. This score will be used as the basis for selecting the "best" prediction when multiple of them overlap.
3) Predictions coordinates were used to build a graph where each prediction was a node, and edges connect predictions that were on the same strand and that overlap for more than 25% of their length (calculated on the shortest prediction). Connected components in this overlap graph correspond to (partially) overlapping gene predictions and we wanted to extract the best one on the basis of the above score.
4) Each connected component in the overlap graph was processed using an iterative procedure as it follows:
    a) To get the node with highest degree in a cluster (i.e. the gene prediction that is overlapped with the largest number of gene predictions), let's call it *A*;
    b) To check the score of its first neighbors (nodes connected to *A*): if *A*'s score is smaller of at least one of the neighbors scores, remove A from the graph; if A's score is the maximum among its neighbors, remove all of them;
    c) If the cluster still exists or it is split into two or more smaller clusters, repeat from (a) for the remaining clusters. Else if only one node remains from the cluster or if all the nodes previously belonging to the cluster are now isolated, move to the next cluster.

At the end of this procedure, we obtained a list of gene predictions that were considered the best within an overlapping cluster, on the basis of the integrated score taking into account evolutionary and expression information. In case of alternative and overlapping transcripts, we ended up with the transcript better conforming to other proteins in the oomycetes and/or to the transcript that has been assembled from RNA-Seq data. Non

overlapping alternative transcripts were treated as if they were independent gene predictions but remains associated to their original parent gene and therefore it can happen that more than one transcript for the same gene was present in the final selected predictions.

Naming conventions

All gene predictions have been named by adding the suffix *PVITvX* to a progressive number. *PVITvX_T* followed by the parent gene progressive number indicates the gene transcript. If multiple transcripts for the same gene have been selected, the additional transcripts have .1 (.2 and so on) appended to the transcript ID. CDSs have been named accordingly as *PVITvX_CDS* plus the progressive number of the parent transcript for a final set of 38,298 genes (**Supplementary Table 3**).

Ribosomal and transfer RNA genes

Ribosomal genes (rRNA) were annotated using RNAmmer[16], using the eukaryotic model and all remaining parameters as default. We were able to find a gene for the 5S rRNA on scaffold-25542 and a gene coding for the 28S rRNA on scaffold-7390 (**Supplementary Table S4**). Both sequences retrieved as first blast hit a gene from the oomycetes with more than 95% identity (**Supplementary Table S5**). Transfer RNA genes (tRNA) genes were identified with tRNA-scanSE[17] with default settings (**Supplementary Table S6**). We found at least one tRNA for every amino acid, in addition to one selenocystein and two suppressor tRNAs. We also found 64 pseudo-tRNA genes.

Estimation of the degree of completeness of the assembly

To measure the degree of completeness of our genome assembly, we considered four different tests:

1. The total size of the assembly is ~83 Mb and estimations of *P. viticola* genome size using Feulgen staining[18] indicate a size of ~110 Mb; therefore, our genome assembly should account for about 75% of the total genome size.

2. After ortholog classification, we estimated the oomycete core genome excluding *P. viticola* to be composed of 1,299 genes among the sequenced oomycete species of which 1,054 also contain *P. viticola* sequences; therefore, the “coding” assembly should be ~81% complete. A similar conclusion was reached when testing the degree of reduction of the size of the core genome when adding the biotrophs in our dataset (**Supplementary Fig. S11**). *P. halstedii*, representing the most deeply sequenced genome among the biotrophs, misses more or less 15% of the genes that are in the *core* genome of the non-biotrophs. This can be considered as a first approximation as the reduction caused by the biotrophic life style. Since adding *P. viticola* determines a reduction of 30-35% of the core genome, the assembly should be estimated at ~80%.

3. We downloaded all *P. viticola* sequences available in the NCBI nucleotide database (N=726) and clustered them by homology to get a non-redundant dataset (N=288) and blasted them against our genome assembly to detect their presence. At 95% identity level cut-off, we obtained a hit for 228 sequences, corresponding to ~84% of the total.

4. We performed a BUSCO[19] analysis to assess the completeness (**Supplementary Table S9**). We obtained 73% complete BUSCO orthologs while this percentage is much higher (over 93%) for *P. halstedii* and *P. infestans*. Most of them are single copy, and the duplicated percentage of our assembly is well in line with the *P. halstedii*'s one, while the *P. infestans* BUSCO orthologs are duplicated in almost 7% of the cases. When summing up the complete and fragmented BUSCO orthologs our assembly covers 87.3% of the 303 sequences.

All the tests performed seem to indicate that our genome assembly is about 75% to 87.2% complete taking into account the genome size.

We also performed an estimation of the true protein number in *P. viticola* (**Supplementary Fig. S12**). We studied the relationship among the number of proteins in the proteome and the number of proteins that we assigned to an ortholog group, and found it to be almost linear. Therefore, we built a regression model excluding *P. viticola* from the analysis and we then used the model parameters to predict the total number of proteins in this organism. By approximating the degree of completeness of our genome to 80%, we obtained a 95% probable estimate in the range 12,952-19,330 proteins, which is in agreement with the 18,335 sequences for which we detect gene expression in our RNA-Seq libraries.

Ortholog classification and oomycete core genome

We identified ortholog groups from 15 oomycete species including *P. viticola* (**Supplementary Table S7**). The comparisons of pairs of genomes were performed using Inparanoid[20], and the pairwise outputs were integrated with QuickParanoid (http://pl.postech.ac.kr/QuickParanoid/). We obtained 16,517 ortholog clusters and among them 6,124 that are present in at least 10 species. *P. viticola* proteins fall in 6,552 clusters, for a total of 10,133 proteins. As a comparison, *Plasmopara halstedii* proteins are found in 7,003 ortholog groups, for a total of 9,233 proteins. The oomycete core genome is formed by genes shared by the 15 oomycete species included in the analysis and is partitioned into 1,054 groups for a total of 50,018 proteins (3,238 from *P. viticola*). Of these, 312 contained exactly one protein per species and were used for phylogenetic analysis. The size of the core genome is quite small because the organisms used to obtain it are phylogenetically very diverse; they all belong to the oomycetes, but they are spread over two orders, Pythiales (*Pythium spp.*) and Peronosporales. The Peronosporales in our dataset are moreover from different families: Peronosporaceae (*P. viticola* and *H. arabidopsidis*) and the *Phytophthora* genus (which is not assigned to any Family). The core genome calculated with *Pythophthora* genomes only is consistently larger, comprising 4,530 ortholog groups. The KEGG annotation of *P. viticola* was used to get the most represented categories in the core genome (**Supplementary Table S8**). The distribution of the size of each ortholog cluster suggests the existence of two large families of protein clusters, the first comprising proteins belonging to clusters with approximately less than ten proteins (i.e. the *accessory* genome), and the second one represented by groups with 15 proteins in average, therefore containing the *core* genes, in addition to most of the remaining housekeeping genes and expanded gene families (**Supplementary Fig. S13**). The presence/absence profile analysis provides support for the topology of the phylogenetic tree obtained using ML methods over an alignment produced by concatenating the 312 proteins found to be present in single copy in all analyzed genomes.

Phylogenetic analyses

Phylogenetic analyses of the oomycete dataset were performed taking advantage of 312 core ortholog groups containing a single protein per genome. We selected these proteins because core proteins that are present in a single copy are less prone to errors in ortholog assignments that might negatively affect the phylogenetic reconstruction. Once the 312 proteins were concatenated, they were aligned using MAFFT[21] using parameters optimized for very long sequences. We obtained an alignment of more than 202,000 positions. This alignment was filtered with Gblocks[22], since it has been shown that removing divergent and ambiguously aligned regions from alignments considerably improve phylogenetic reconstructions[23]. The filtered alignment still had 78,868 aligned positions 48% of which are perfectly conserved in all of the species under analysis and the remaining are partitioned into 27,340 patterns. Phylogenetic trees were built using PhyML[24] and RAxML[25]. PhyML and RAxML returned identical topologies with slightly different bootstrap values and branch lengths (**Supplementary Fig. S14)**. The topology obtained was in agreement with the one reported in Sharma *et al.*[26] and McCarthy *et al.*[27] suggesting that taxonomical refinement of the oomycetes might be necessary because the molecular information obtained during the last studies strongly suggests that *Plasmopara* should be placed within the *Phytophthora* clade. This is also supported by full-proteome comparisons, indicating that the distributions of identity percentage of the first blast hit are not very different for *Phytophthora-Phytophthora*, *Plasmopara-Plasmopara* or *Plasmopara-Phytophthora* comparisons (**Supplementary Fig. S15)**.

Comparative genomics of effector proteins

We grouped the RxLR effectors into RxLR and RxLR-like families. Proteins that possess the distinctive RxLR motif (defined as a match to the motif R[A-Z]LR) within 60 amino acids from a signal peptide cleavage site (SP) and those missing the SP but having the RxLR occurrence and the additional and characteristic motif EER (defined by the regular expression [ED][ED][RK]) within 150 amino acids are grouped in the RxLR family (**Supplementary Fig. S16**) .

We opted for this rule because gene predictions can be partial and could therefore miss the N-terminal where the SP is located (**Supplementary Fig. S17**). To further reduce the false positives, we calculated the probability of each RxLR occurrence by shuffling the protein sequence for 2,000 times and counting the number of times that an RxLR was found. Only motifs with a probability smaller than 0.05 in the shuffled sequences were further considered (**Supplementary Table S10**). Code for performing this analysis is available upon request.

However, we might expect that the RxLR motif is slightly different in different oomycete groups (**Supplementary Fig. S18**). *Pseudoperonospora cubensis* for instance has effectors with homology to *P. infestans* RxLR effectors that carry a QxLR motif. These proteins resulted to be localized in the host plant nucleus, strongly pointing towards a role in pathogenesis[28]. Similarly, *Albugo candida* has experimentally verified effectors with a RXL[KQ] motif, while in *Pythium* species the RxLR motif is considered to be absent[29]. For this reason, we also considered and included alternative RxLR motifs in our study.

We classified as RxLR-like the effectors that share homology to RxLR proteins identified through regular expression matching. As *baits* for retrieving homologs, we used the proteins with secretion signal + RxLR motif within 60 residues (+EER motif within 150), together with those with secretion signal and RxLR or RxLR and EER within 150 residues, for a total of 1,100 proteins, coming from all oomycetes considered in this work. These

were blasted against the oomycete database using blastp. The blast output was filtered and only alignments with an e-value ≤ e^{-5} and id*qcovs ≥ 5,000 were retained. The latter threshold allows using both the information coming from the difference in length among query and subject, and the homology. All the 15,876 oomycetes proteins retrieved in such way were then blasted all-against-all. The output was filtered and parsed to prepare it as an input to the MCL algorithm to detect RxLR communities using as edge weights –log10(e-value). The e-value was replaced by e^{-200} when equal to 0 to avoid taking the logarithm of zero. After filtering at e-value ≤ 0.01, the blast graph still contained 15,646 proteins. The MCL algorithm was run multiple times, using increasing inflation parameter values. Since this parameter controls the granularity of the resulting clustering, scanning a range of values allows exploring the cluster structure of the homology graph more in detail. When running the MCL with an inflation parameter of 2, 2.5, 3 and 4, we obtained 555, 630, 696 and 764 RxLR clusters respectively, indicating a wide diversification of this gene family in oomycetes.

We analyzed the MCL output in terms of the presence of i) signal peptide and ii) canonical and alternative RxLR motifs using an extended regular expression ([QRK]xL[KR]) together with relaxed rules for the distance from the signal peptide. Since all "rules" for RxLR identification are based on the features of a few *Phytophthora* genomes, the identification of these effectors in other genera/families might require relaxed constraints. On the opposite, it is known that RxLR motifs are combined to a wide range of protein domains that are found in non-effector proteins; therefore, we had to be cautious with the retrieval of homologous proteins that likely contains false positive effectors simply sharing a domain with effectors. For this reason, we only focused on the RxLR-like characterized by the presence of canonical or alternative RxLR sequence motifs. The association of the RxLR motif (or variants thereof) with known protein domains, was explored by building a co-occurrence graph of Pfam domains with the RxLR signature (**Supplementary Fig. S19**). We selected *P. viticola* RxLR-like effectors as those proteins still in a multi-protein cluster at the most stringent clustering (I=4), for a total of 802 proteins (gene set called *RxLR-like All*). Of these, 40 also have a predicted signal peptide for secretion together with one (22 sequences) or more occurrences of the RxLR (variant) together with an EER occurrence within 150 residues (gene set called *RxLR-like Motifs*). We also found that 193 proteins in the *RxLR-like All* set are positive to signal peptide prediction, corresponding to a 5.7 fold enrichment with respect to the total proteome (p-value=0.0).

Crinkler (CRN) proteins have been defined on the basis of the presence of characteristic motifs, whose function is, however, still unknown. The well-known LFLAK signature as identified by meme on 430 CRN sequences from Haas *et al.*[5] is considered to be a hallmark of this family of effectors. Presence of the LFLAK motif (from residue 50 in **Supplementary Fig. S20a**) has been previously used for the identification of Crinklers in *Phytophthora* species[5]. However, this motif is conserved when considering *Phytophthora* species but not when including more sequences and other organisms[5]. Certain variability exists also within the LFLAK motif of previously annotated *Phytophthora* Crinklers. Therefore, when additional species are considered, we can expect an increased variability of the signature also in this case. In addition to the LFLAK, other sequence motifs can be present in Crinkler proteins, such as the so-called VVP motif (**Supplementary Fig. S20b**), which is usually localized downstream of the former. In Haas *et al.* [5] CRN proteins were classified by building a PSI-blast profile and an HMM from only 16 previously identified CRNs. However, the small number of proteins used for training might give a model over fitted on those sequences. This would be a problem if the constrains governing the evolution of a distinctive motif might change depending on the species considered; as in the case of RxLR effectors, this can be expected given the phylogenetic distance among

some of the organisms considered in this work. Since our dataset comprised several non-*Phythophthora* species, we tried to improve the CRN identification strategy: we tried to align the 430 CRN sequences identified in Haas *et al.*[5] to build a more general HMM model, but the alignments were of very low quality, making the building of a meaningful HMM impossible using the full sequences. We decided therefore to use the information contained in shared sequence motifs and their combination to implement an alternative classification strategy not requiring the global alignment of all the sequences. Since we have seen that the LFLAK motif, probably the most conserved one, has itself some inherent variability, we speculated that maybe a correct classification of these proteins might come from integrating profiles of presence and absence of several sequence motifs instead of focusing on only one or two of them by assuming they are universal. Variability of the motifs has moreover to be modeled in a more satisfactory way than with regular expressions, where the alternatives at some position all have the same weight. We decided to let MEME discover conserved motifs in a dataset of *bona fide* CRN proteins and then we used them to identify novel CRNs. MEME was run on the whole dataset of *P. infestans* CRN proteins asking for 10 motifs of 30 residues (-mod zoops, allowing for zero or one motif per sequence). MEME was able to find 9 motifs. The motifs are encoded as log probability matrices, where each ij cell indicates the probability of finding residue j at position i of the sequence motif. We used these 9 motif models to scan all oomycetes proteomes using a sliding window approach where we consider each position of a window as evolving independently from the remaining such that the score of each window is the sum of the log probabilities at each position in the model. The score of a sequence is then the maximum score over all the windows. We calculated two matrices of motif probabilities, one for *bona fide* CRN proteins, and the other for 17,700 *P. infestans* proteins to be used as negative cases (the Crinklers still present in this dataset are a small fraction of the total and should not affect the training procedure). Once the score matrices are ready, we train a Support Vector Machine in Matlab to provide the classification. SVM models require training on a positive dataset. We therefore selected the 200 best scoring CRN sequences. The best performances were obtained when the input matrices were re-scaled to the range [0 1] and then log10 transformed. Cross-validation with an equal number of randomly sampled negative cases allowed to estimate the error rate of the model. For 100 times we built a different negative dataset by randomly sampling the 17,700 negative sequences, and we trained the SVM model, testing its performances by cross-validation. The error was estimated each time using 100 repetitions where 70% of the data (35% from the positive and 35% from the negative dataset) was used for training and the remaining 30% for validation. At each run of the procedure, the data was sampled randomly from the positive and negative dataset. The SVM models achieved a cross-validation average error of 0.0095, indicating that wrong classifications occurs in less than 1 case in 100 (**Supplementary Fig. S21**). The model predicted 13 additional CRN proteins from the negative dataset, 8 of which are indeed annotated as Crinklers but were not included in the CRN dataset from Haas *et al.*[5]. The same procedure was run with probability matrices corresponding to different MEME runs to identify the best motif length for classification. We tested motif widths of 5, 10, 15 and 30. The SVM classifier using the latter data showed the best performances and was selected for further analysis on all oomycetes that were considered in this work. Since Crinklers are secreted, the presence of the signal peptide can be used *a posteriori* to further increase the stringency of the classification or to calculate an enrichment of predicted CRNs in secreted proteins that can give an external validation.

The strength of the SVM-based classification scheme is that it does not depend on the presence of one or a few well defined motif, but on the combined presence/absence of several motifs, and therefore it should allow making better inferences with respect to a

rigid regular expression. For instance, *A. euteiches* possesses [FL]xLYLALK and *P. ultimum* has LxLYLARK motifs that are considered LFLAK variants and should therefore be considered *bona fide* CRN motifs. These instances would be missed using simple regular expressions based on *Phytophthora* Crinkler proteins, and a regular expression encoding all the variability would become extremely unspecific. On the contrary, the probability matrix approach allows a better representation of the variability of the motifs in a position specific way and therefore should improve the identification of marginal similarities. Some divergence from the most probable motif is tolerated and variants are scored on the basis of their probability. Calculation of the probability clearly poses a problem concerning the threshold to consider a motif to be present. With our approach we train a model with *bona fide* Crinkler proteins and we let it find out CRNs among new proteins, without the need for specifying thresholds. Partially conserved motifs are moreover combined with others increasing the probability that the model will correctly classify more proteins.

To provide a more classical result, we also searched for CRN using the widely used regular expression approach. The regular expressions used were L[A-Z]L[FY]LAK and [IVM]HVL[VI]VVP for the LFLAK and VVP motifs, respectively.

Genome analysis of *Pythium ultimum* revealed that the YxSLK motif was enriched in the secreted proteins with respect to the full genome. 32 out of 44 members identified in *P. ultimum* genome were induced from 2- to 40-fold during *Arabidopsis* infection[29]. The motif is constrained between residues 61-80. As previously, we define a YxSLK group containing proteins with an occurrence of the regular expression Y[A-Z][ST][LV][KR] (when indicated, within the N-terminal 100 residues of the protein and together with a signal peptide). This extended regular expression derives from a reanalysis of the motif found by MEME when the input sequences are all the proteins annotated as belonging to "*Family 3*" in Lévesque *et al.*[29] (**Supplementary Fig. S22**). Additional non default parameters were "-protein -w 5 -nmotifs 20 -mod zoops -minsites 10". MEME[30] analysis returned a motif corresponding to the YxSLK pattern ranking 9th over 20 requested motifs of length 5. This YxSLK pattern appears to be present in 36 out of the 51 "*Family 3*" sequences, while some of the motifs ranking higher are present in almost all of the sequences. *P. viticola* has the largest number of members of this class among the biotrophs, comparable to the *Pythium* species, but much lower than *Phytophthora* species (**Supplementary Table S14**).

Randomization of the sequences positive for one of the regular expressions r for m times allowed to count the number n of occurrences of the pattern r given a random distribution of residues and therefore to discern the occurrences observed by chance (e.g. for compositional biases in the protein sequence). For all motif searches using regular expressions, we calculated the significance of the occurrences found and we considered further only those significant at 0.05. The empirical p-value was calculated as: $p = \frac{n+1}{m}$.

Apoplastic effectors were identified by exploiting available HMM models from Pfam[31] (http://pfam.xfam.org/), as indicated in **Supplementary Table S15**. The scanning was made using HMMER[32] with a threshold of e^{-6} on the "E-value seq" field.

Gene expression profiling

We performed an infection of *Vitis vinifera* cv. ENTAV115 with *P. viticola* (isolate 'PvitFEM01') and harvested non-infected and infected plants at five different time points (0, 24, 48, 72, 96 and 168 hours post-infection, hpi) with 20-25 plants in two replicates each. Each replicate corresponded to an independent infection experiment. We also collected sporangia of *P. viticola* from infected material at late time points (96 and 168 hpi). The non-infected plants, infected ones and the sporangia were pooled to obtain three samples

called C, I and S, respectively. After RNA extraction using the Spectrum plant total RNA kit (http://www.sigmaaldrich.com/) we built the RNA-Seq libraries using the TruSeq RNA library prep kit (www.illumina.com) following the manufacturer's instructions. We performed a paired-end RNA-Seq (PE 2x100) from libraries C, S, I and duplicate libraries of non-infected and infected tissues collected at 0, 24, 48, 72, 96 and 168 hpi. In total, 25 libraries were sequenced on a HiSeq 2500 platform (www.illumina.com). The number of reads obtained for each library ranged from 20.5 to 97 million reads whereas the absolute number of reads mapping to *P. viticola* and *V. vinifera* genomes reached 68.5 million in the non-infected 72 hpi library (**Supplementary Table S17)**. Interestingly, only 49 reads from the library C mapped concordantly on the *P. viticola* genome draft assembly, indicating that if a contamination from grape DNA still exists in our draft assembly, it is not significant. Sequences obtained from the pooled libraries were used for the validation of the gene prediction and supported the *P. viticola* transcript annotation. Reads of the pooled libraries aligned on *P. viticola* draft assembly using TopHat with no mismatches detected 14,011 loci in library I (corresponding to a total of 15,253 predicted genes), 17,024 loci in library S (18,293 genes). In total, if all genes predicted were combined we validated the expression of 17,314 loci (18,335 genes).

Differential expression analysis of *P. viticola* and *V. vinifera* transcriptomes was performed using TopHat followed by cufflinks, cuffmerge and then cuffdiff[15,33]. TopHat was run with option –N 0 (number of mismatches) using the reads from the different libraries independently. Cufflinks was run using the reads accepted by TopHat, to estimate gene expression abundances of the transcripts annotated in the gff file provided as input. Options were as default except that we applied corrections for multiple mapping and composition bias of fragments (options -u -b). The transcripts.gtf files produced for each library by cufflinks were then merged for *V. vinifera* and *P. viticola* separately using cuffmerge. The merged.gtf transcripts were used as an input to cuffdiff to evaluate differential expression. The transcripts returned by cufflink programs are a combination of annotated ones, present in the annotation file provided during the run, and a certain number of newly discovered transcripts that span genomic regions not covered by any annotated transcript in the provided gtf file. Here we focus on the former.

Genes significantly changing their expression level during infection in *Vitis vinifera* were identified using cuffdiff with the option –time-series, such that every time-point is compared to the previous one. We detected 740 differentially expressed genes (DEG) (at FDR ≤ 0.001). The list showed no overlapping at all with the DEGs identified with respect to the control libraries (whereby all gene expression levels are tested against the t0 time point). No NBARC domain containing genes were detected as differentially expressed with the time series option while 116 of them come out as DEGs when comparing the infected and the control libraries. The enrichment analysis revealed that genes responding to stimulus are the most differentially expressed (**Supplementary Table S28**).

Transcriptional profiling of *P. viticola* during infection

We detected the expression of 18,335 *P. viticola* genes at all time points comprising 1,061 transcripts coding for proteins with a predicted signal peptide. We detected only few reads mapping on the *P. viticola* genome at 24 and 48 hpi (**Supplementary Table S19**). Given the small number of reads from *P. viticola* in the two first time points, we analyzed the differential expression of genes starting at 72 hpi. Although it is not possible to perform a differential gene expression at early time points we, nevertheless, identified the most induced *P. viticola* genes at those time points. At 24 hpi we detected gene expression for 3,680 *P. viticola* genes. However, using cufflinks we found only 192 genes with expression

levels classified as significantly different from 0, which is clearly an effect of the low number of *P. viticola* reads in this sample. Using our GO annotation of the *P. viticola* proteome (**Supplementary Table S1**), we detected enrichment of several GO categories at FDR ≤0.05 (**Supplementary Table S19**), indicating an active metabolic activity of *P. viticola* during the early phase of infection coupled with stress response activities that are likely a consequence of the first defense barriers put in place by the plant. We also detected a significant enrichment in proteins with predicted signal peptide both considering the transcripts significantly different from zero (19/192 vs 1061/18335, p-value=1.5 e^{-2}) and the entire set of transcripts expressed at 24 hpi (270/3680, p-value= 1.6 e^{-5})(**Supplementary Table S19**). A similar situation concerning induced metabolic processes and enrichment of proteins containing a signal peptide was observed at 48 hpi, indicating that metabolic activity is high at these stages of infection as well as protein secretion (**Supplementary Table S19**).

At 24 hpi, we detected the expression of 1 out 68 CRN, 10 out of 57 RxLR-regexp (4 with signal peptide), 259 out of 802 RxLR-like (56 with a predicted secretion signal) and 56 out of 308 YxSLK (8/87 with signal peptide) effectors. Our data suggest that CRN are rather deployed at later time points during the infection and subsequently kept expressed at high level (**Supplementary Fig. S23**). With 32% of the transcripts detected at 24 hpi, the RxLR-like group is the most represented effector group at this time point, followed by the YxSLK effectors. We also detected significant expression of many transcripts corresponding to proteins with a predicted signal peptide, but not classified as effectors, that were grouped together according to the expression profiles (**Supplementary Fig. S23**). Our analysis indicates that the RxLR expressed at 24 hpi are enriched for the presence of the Pfam model Pkinase and Pkinase-Tyr (protein phosphorylation) and to a lesser extent EF-hand (calmodulin), zf-C3HC4 and zf-RING (ubiquitination), LRR. The latter is particularly interesting as it is a domain known for its presence in plant resistance genes. We find this domain in a total of 111 *P. viticola* proteins of which four are RxLR homologs that do not possess canonical or alternative RxLR occurrences (**Supplementary Fig. S24**). Besides their identification in several oomycetes, no information about their putative role is available. Interestingly 15 of these genes are expressed at 24 hpi and some of them have their maximum expression level at this time, representing promising effector candidates for further studies (**Supplementary Fig. S25**). The YxSLK effectors expressed at 24 hpi are enriched in Reprolysin (a zinc metalloprotease) and other peptidases, as the apoplastic effectors. Genes coding for proteins not classified as effectors but expressed at 24 hpi and with a predicted signal peptide are enriched in specific domains such as glyco-hydrolase, Cu-oxidase, thioredoxin, pkinase and jacalin.

The libraries obtained for the 24 hpi and 48 hpi contained too few reads from *P. viticola.* Nonetheless, we were able to detect 1,284 DEGs from the remaining contrasts; 538 of these genes were also expressed at 24 hpi, indicating that some of the most early expressed genes also change their expression level at later time points. We exploited the GO functional annotation of the *P. viticola* genome obtained using Argot2[12], to assess functional enrichment (**Supplementary Table S21**) and identified several processes (FDR ≤ 0.01). We observed an enrichment of processes that are related to development of anatomical structures and likely to germination, biosynthesis of the germ tube, hyphae and the haustorium. We also detected an enrichment of catabolic activities, such as glycolysis, fatty acid beta-oxidation using acyl-CoA dehydrogenase, tryptophan catabolism, proteolysis, indicating an active metabolism of *P. viticola* during the infection.

Agrobacterium infiltration assays and qRT-PCR

The coding regions, including the start codon (ATG), of *P. viticola* effectors PVITv1003209, PVITv1005727, PVITv1018092, PVITv1020941, PVITv1016922, PVITv1021061, PVITv1008294, PVITv1008311 were amplified from cDNA of sporangia (prepared with SuperScript VILO cDNA synthesis kit from ThermoFisher Scientific) using the primers indicated in **Supplementary Table S35**. The PCR was performed using the Phusion high-fidelity DNA polymerase (ThermoFisher Scientific) following the manufacturer's instructions and cloned in the pENTR/D-TOPO vector (ThermoFisher Scientific). After verification of the sequences by Sanger sequencing the open reading frame without the signal peptide was amplified by PCR and restored by adding an ATG in frame. Both cDNA version with (+sp) or without (Δsp) signal peptide where then recombined using the Gateway technology in the pK7WG2D binary vector. As a control for the infiltration assays, the MCS of the vector pBluescrit SK(+) was amplified and also inserted in the pK7WG2D binary vector and called "empty vector". After verification of the correct cloning by Sanger sequencing the vectors were introduced in *Agrobacterium tumefaciens* GV3101. The infiltration assays were carried out in sterile conditions as described in Zottini *et al.*[34] on *Vitis vinifera* cv. Sultanina and *Vitis riparia in vitro* grown plants. The phenotype of plants was visualized and photographed one week after infiltration. After taking a picture the leaf was then stained using Trypan blue as described in Roetschi *et al.*[35]. The experiment was repeated ten times with 20-25 plants at each experiment. Infiltrated leaves were also flash frozen in liquid nitrogen and the RNA extracted using the Spectrum plant total RNA kit (http://www.sigmaaldrich.com/). After reverse transcription of one μg of RNA using the SuperScript VILO cDNA synthesis kit (ThermoFisher Scientific), the real-time PCR on the effector and GFP was performed using the 2x SYBR green qPCR master mix (www.bimake.com) on a ViiA7 real time PCR system (ThermoFisher Scientific). The fold induction of RxLR_ PVITv1008311 normalized to the house keeping gene F_PveIF1b was calculated using the comparative Ct method[36]. The same real-time PCR conditions were used to measure RxLR_ PVITv1008311 expression levels *in planta* during the infection time course on Pinot Noir ENTAV115 as well as in sporangia in Figure 3a and 3b.

Metabolism

We studied the *P. viticola* metabolic abilities by assigning KEGG orthologs groups to proteins using KAAS[11]. This analysis was in addition performed for *H. arabidopsidis* (the closest biotroph) and *P. infestans* (the closest hemibiotroph), to characterize commonalities and differences. Maple[37] was used to compare the metabolic networks of the three organisms at once (**Supplementary Table S26**).

sRNAome and degradome

To identify sRNAs from *P. viticola* we first filtered the sRNA reads by mapping them on the grapevine genome and then on an ensemble of bacterial genomes to remove most contaminants. The 19-30 nt sRNAs that passed this filtering step were then mapped on the *P. viticola* genome. The reads mapping uniquely on our draft *P. viticola* assembly with 100% identity over the entire length of the read were further considered. sRNA target predictions were performed using SeqTar[38]. We first explored regulation of *P. viticola* transcripts by endogenous sRNAs. To detect sRNA targets, we ran SeqTar using the 21-nt long sRNAs perfectly mapping on *the P. viticola* draft assembly, the *P. viticola* transcripts, and PARE reads obtained from a pool of the time points at which we performed sRNA. All parameters were default, except a 100 shuffling of the sRNAs to calculate the significance

level of the number of mismatches of an sRNA on a given transcript. The SeqTar output was filtered at mismatch p-value≤0.001, valid peak height p-value≤10^{-10} and binding score p-value≤0.001. After applying the above filters, we obtain 68 putative regulations, implemented by 65 different sRNAs on 39 transcripts (**Supplementary Table S32).**

To detect *P. viticola-V. vinifera* cross-regulations, we ran SeqTar[38] using 21-nt long sRNAs perfectly mapping on the *P. viticola* draft assembly, grapevine transcripts as targets, and a degradome library obtained from a pool of all of the infected time points at which we performed sRNA sequencing. All parameters were default, except that a 100 shuffling of the sRNAs was used to calculate the significance level of the number of mismatches of an sRNA on a given transcript. The SeqTar output was filtered at mismatch p-value≤0.001, valid peak height p-value ≤10^{-10} and binding score p-value≤0.001. After applying the above thresholds, we obtained 344 putative regulations of grapevine transcripts, implemented by 318 different *P. viticola* sRNA sequences on 296 different *Vitis* transcripts (**Supplementary Table S33 and S34**).

We also explored the miRNA-mRNA regulatory network in *V. vinifera*. The analysis was performed as for *P. viticola*; the output was filtered to retain only predictions with p-value mismatch≤0.001, p-value binding score≤0.001 and p-value of the number of valid reads≤10^{-10}. After filtering we retained 1,662 putative regulations for 1,327 sRNAs that regulate in total 889 *Vitis* genes. Among the putative regulators, we detected nine known miRNAs (**Supplementary Table S31**).

Supplementary figures

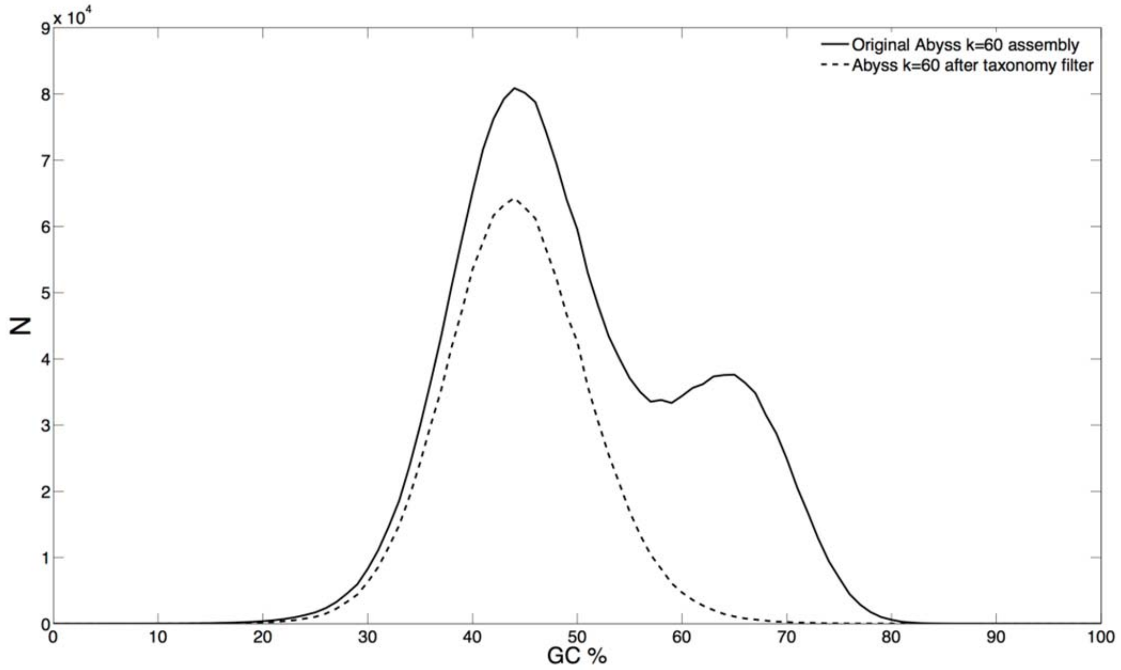

Supplementary Figure S1. GC content distribution for the original and the taxonomy-filtered assemblies.

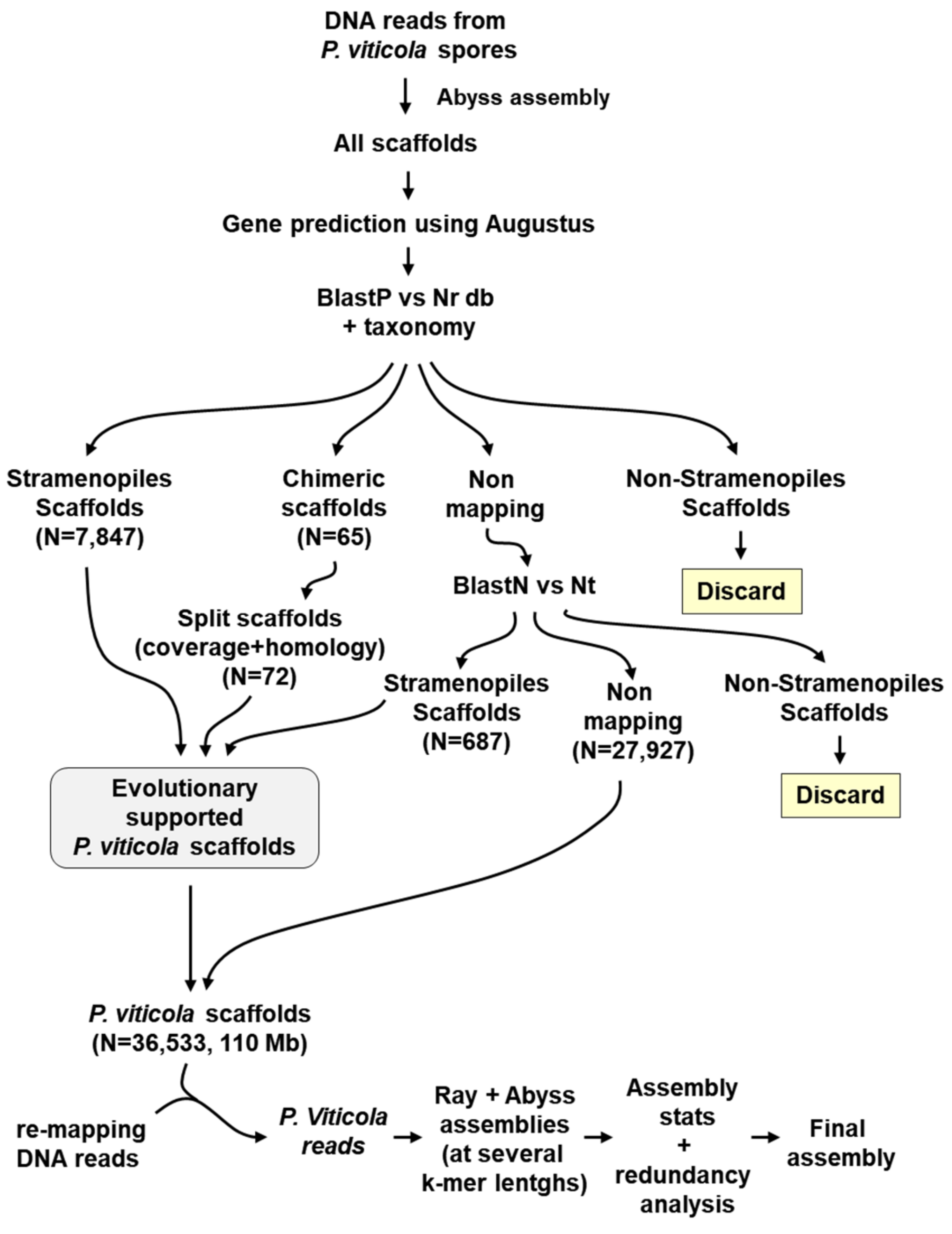

Supplementary Figure S2. Strategy implemented to filter out sequences not coming from *P. viticola*.

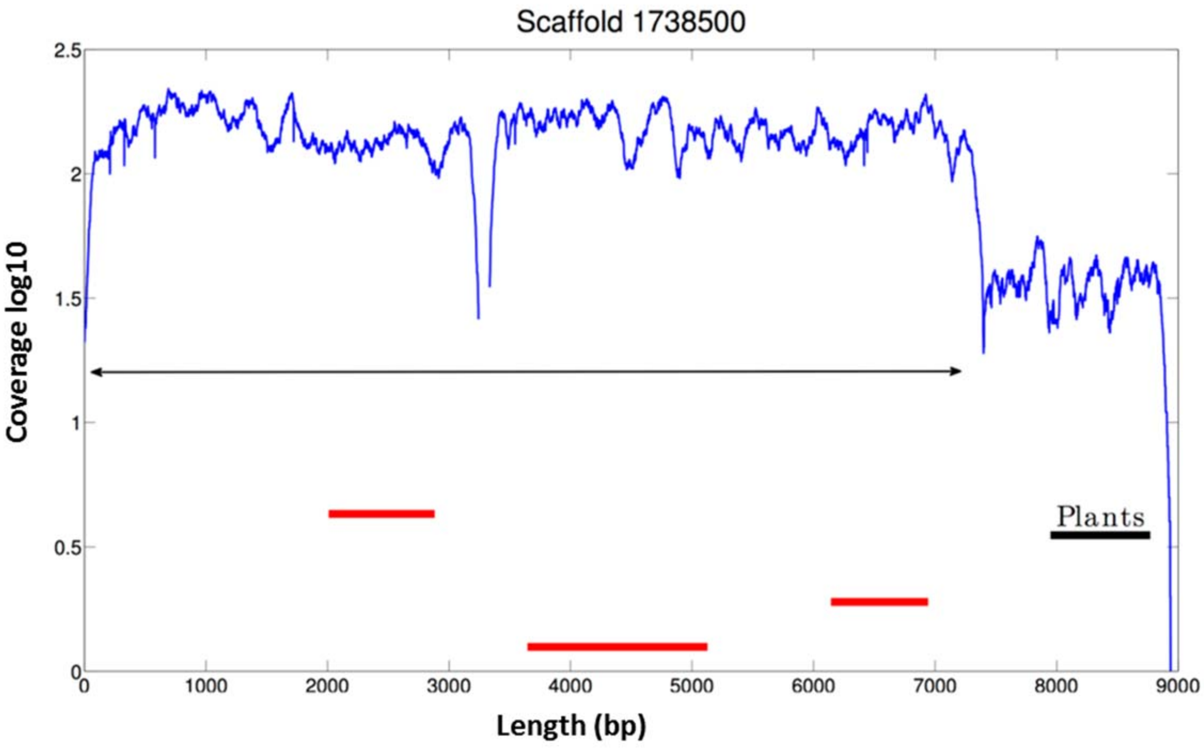

Supplementary Figure S3. Example of a chimeric scaffold.

The first part of the scaffold codes for three proteins with homology to Stramenopiles and it is therefore likely part of the *P. viticola* genome. The average coverage of this region is well above 100 (y axis is in log10 scale). The remaining 1,500 nucleotides have a much lower coverage and homology to plant sequences. Therefore, the scaffold was included in the *P. viticola* assembly after deleting the low coverage region.

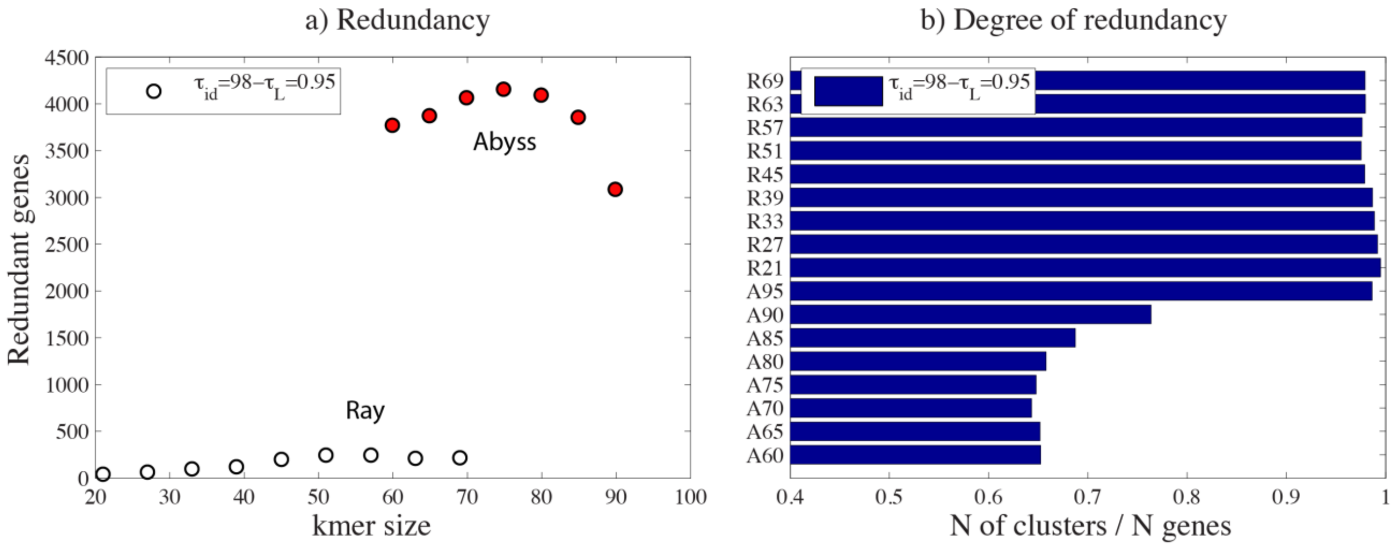

Supplementary Figure S4. Clustering sequences that share more than 98% identity and have lengths not differing by more than 5% identified groups of unique sequences.

The number of genes considered as redundant (**a**). The ratio of the number of unique sequences and the number of predicted genes indicates that the coding genome is highly redundant in ABySS assemblies (from A60 to A95), as this ratio is only 0.6-0.7, while for Ray assemblies (From R21 to R69) this ratio is close to 1 (**b**).

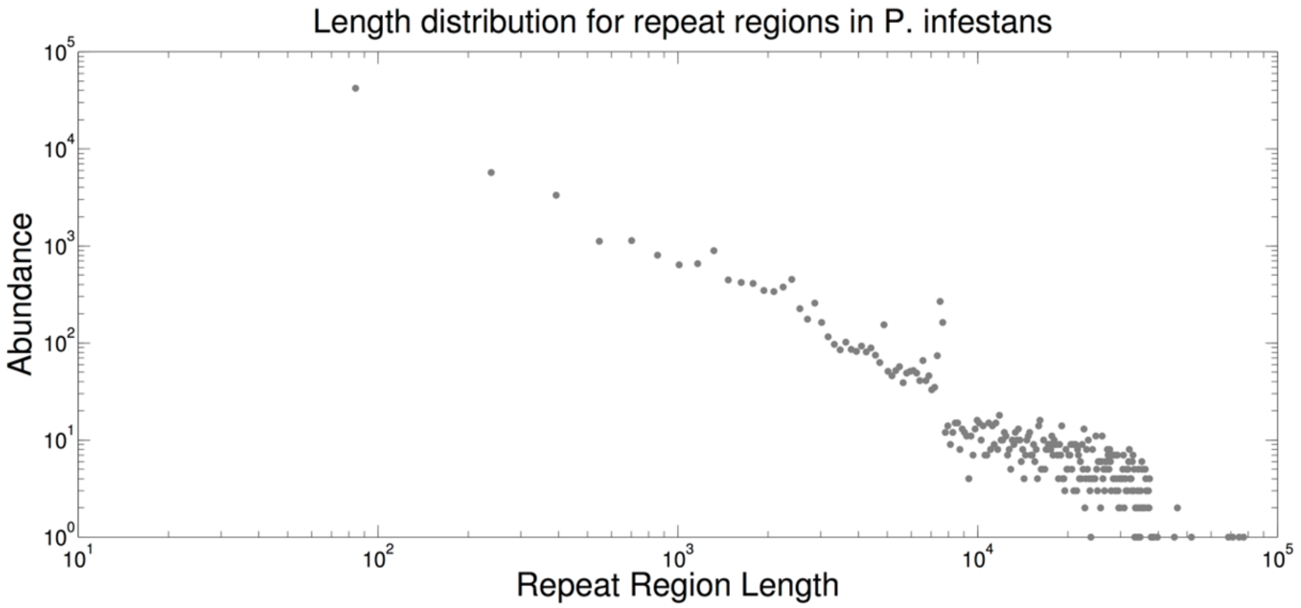

Supplementary Figure S5. Length distribution of *P. infestans* repeat regions

Most repeats (80%) are below 500 nucleotides, but still there are over 12,000 repeats longer than that. The data was obtained from Haas *et al.*[5].

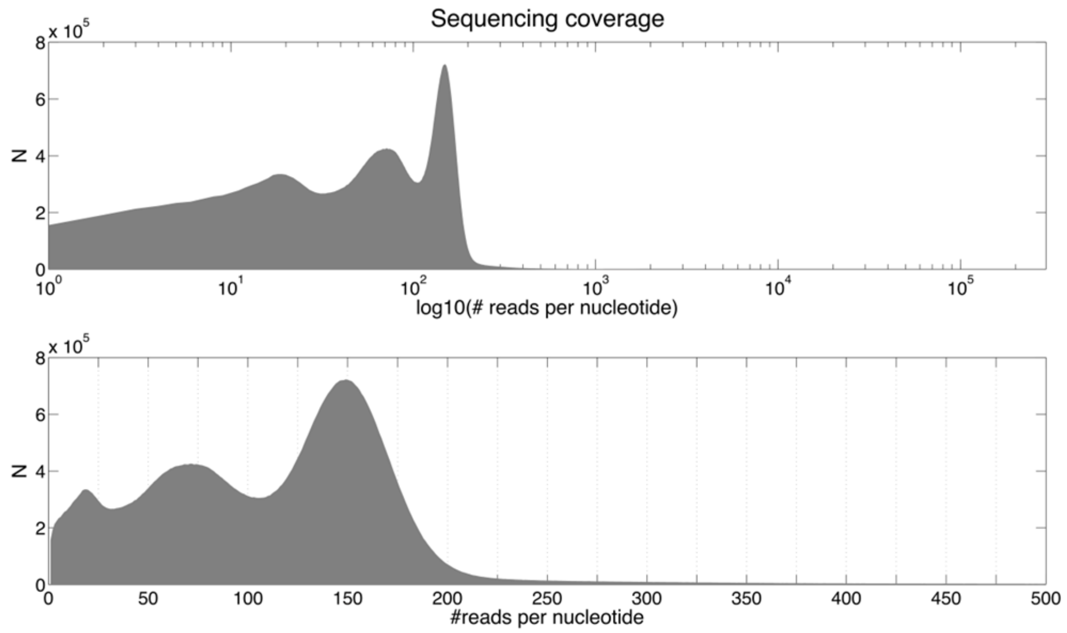

Supplementary Figure S6. Coverage per nucleotide of the final assembly.

The shape of the distribution is likely the result of different haplotypes of *P. viticola* contained in the inoculum used for infection (see supplemental note for statistics about the final assembly).

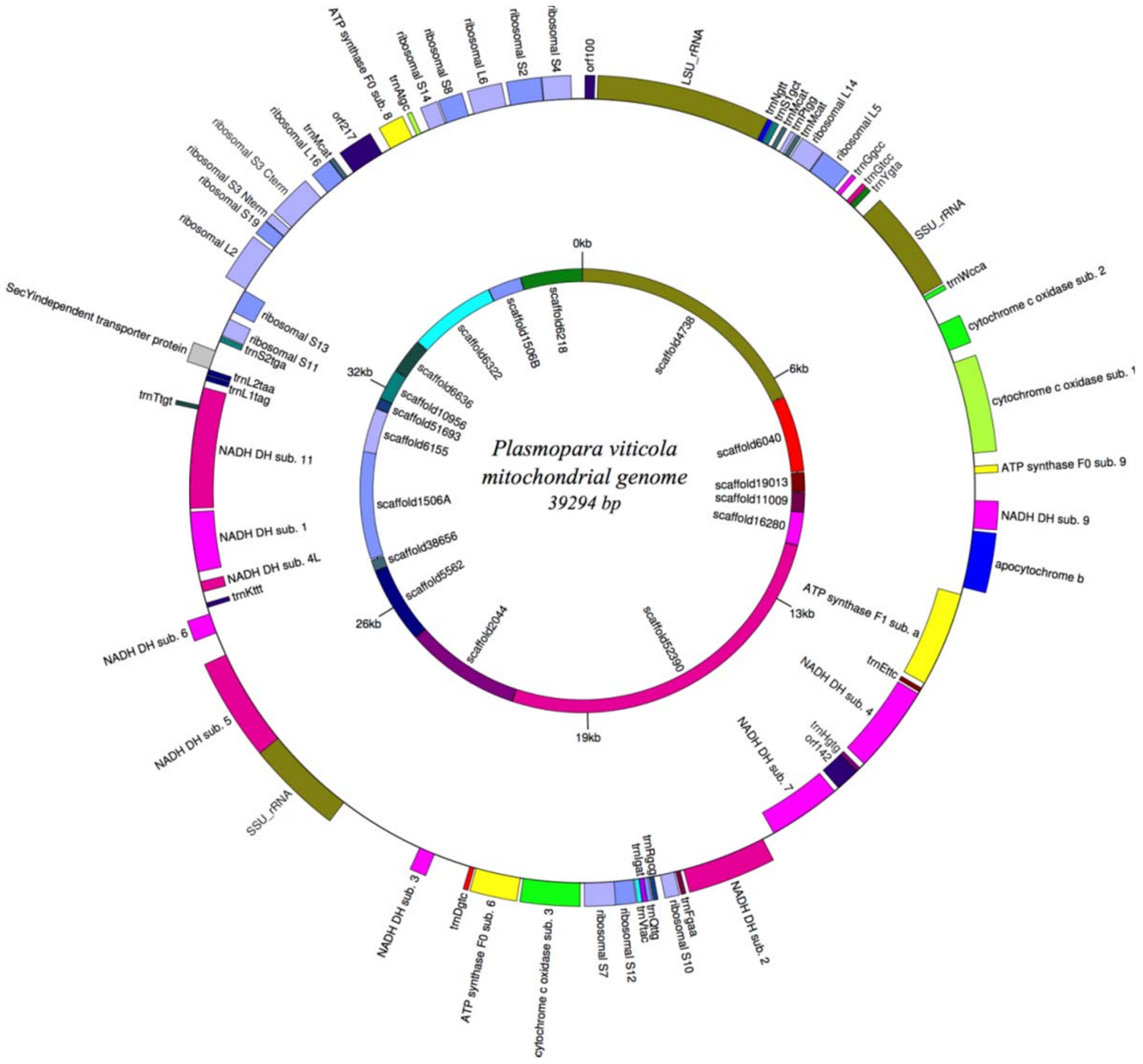

Supplementary Figure S7. Schematic representation of the assembled *P. viticola* mitochondrial genome.

The outer layer corresponds to the predicted genes, while the inner one indicates the scaffolds used for assembly. The tRNAs are indicated by the abbreviation “trn”, followed by the letter corresponding to the amino acid bound and the recognized codon.

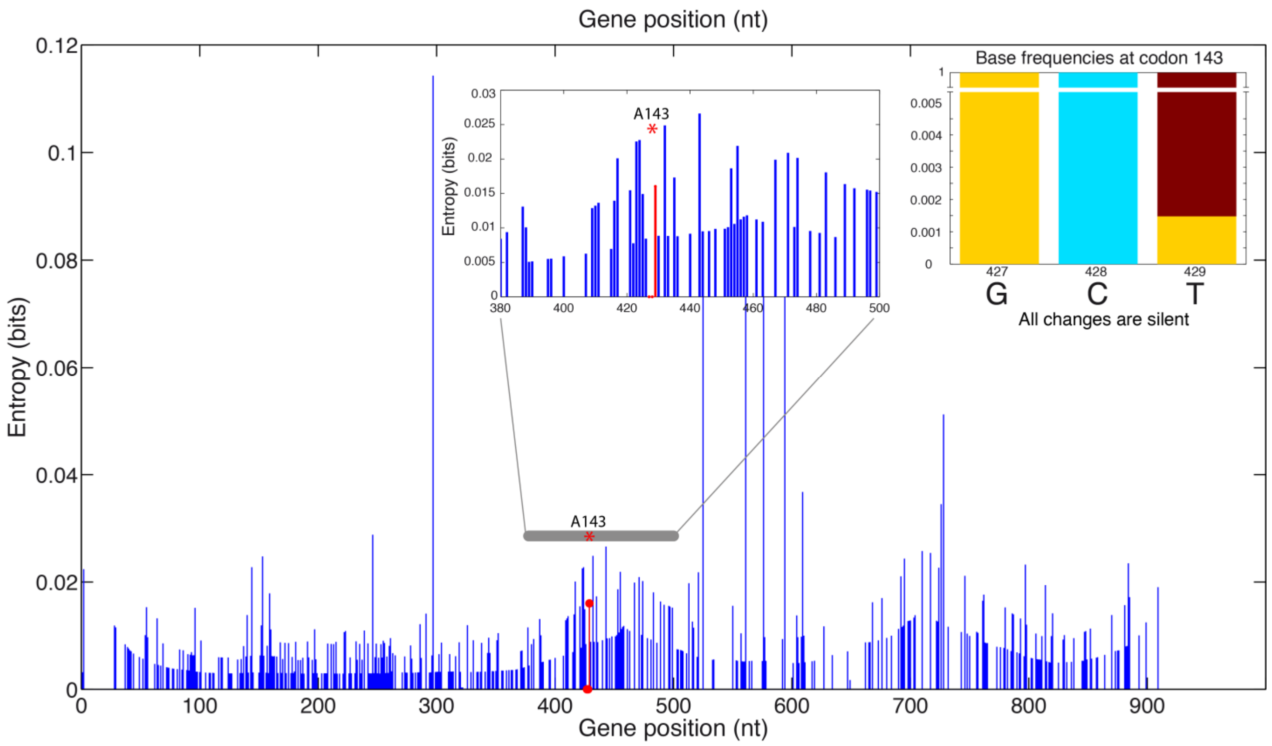

Supplementary Figure S8: Variability in the mitochondrial apocytochrome b gene sequence.

The variability was obtained by mapping DNA reads and retrieving SNPs using *mpileup* in samtools. Entropy of each position of the gene was calculated by extracting all possible nucleotides found in the original reads and plotted to show the existence of multiple “haplotypes” or polymorphisms. A single amino acid change, G143A, in the apocytochrome b protein is known to confer resistance to fungicides acting as Quinone outside Inhibitors (QoI). The mitochondrial assembly of ‘PvitFEM01’ revealed an Alanine at amino acid position 143. The variability at that position is moderate and always corresponds to silent changes. This indicates that the haplotypes that were sequenced are all resistant to QoI fungicides.

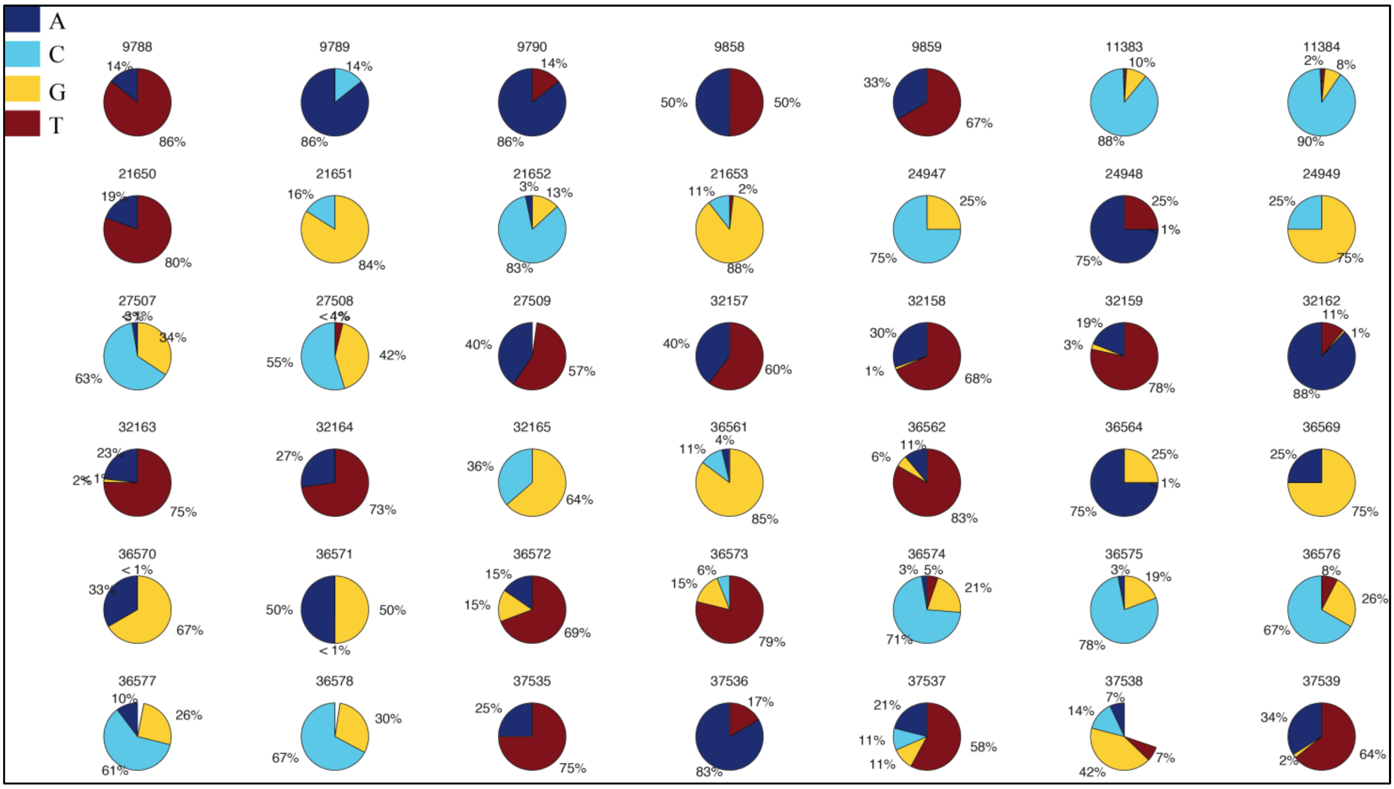

Supplementary Figure S9: Base composition at 42 mitochondrial positions with an entropy above 0.5 bits.

All of them are outside annotated genes (protein coding, tRNAs or RNAs). Empty slices stand for Ns.

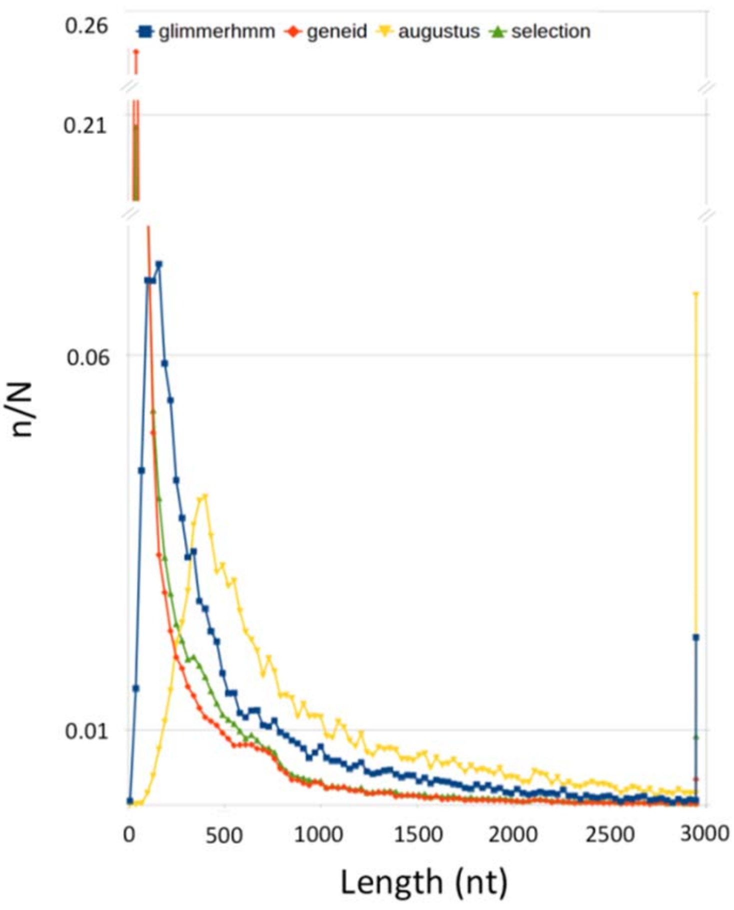

Supplementary Figure S10. Length distribution of the transcripts predicted by three gene finders.

Note that the Y axis has been broken two times to include the maximum peaks of the selection and GeneID distributions. GeneID outputs gene predictions that are on average shorter than those provided by GlimmerHMM and Augustus, with over 28,000 predictions shorter than 70 nucleotides (38% of all predictions Vs <1% and <0.1‰ respectively for GlimmerHMM and Augustus).

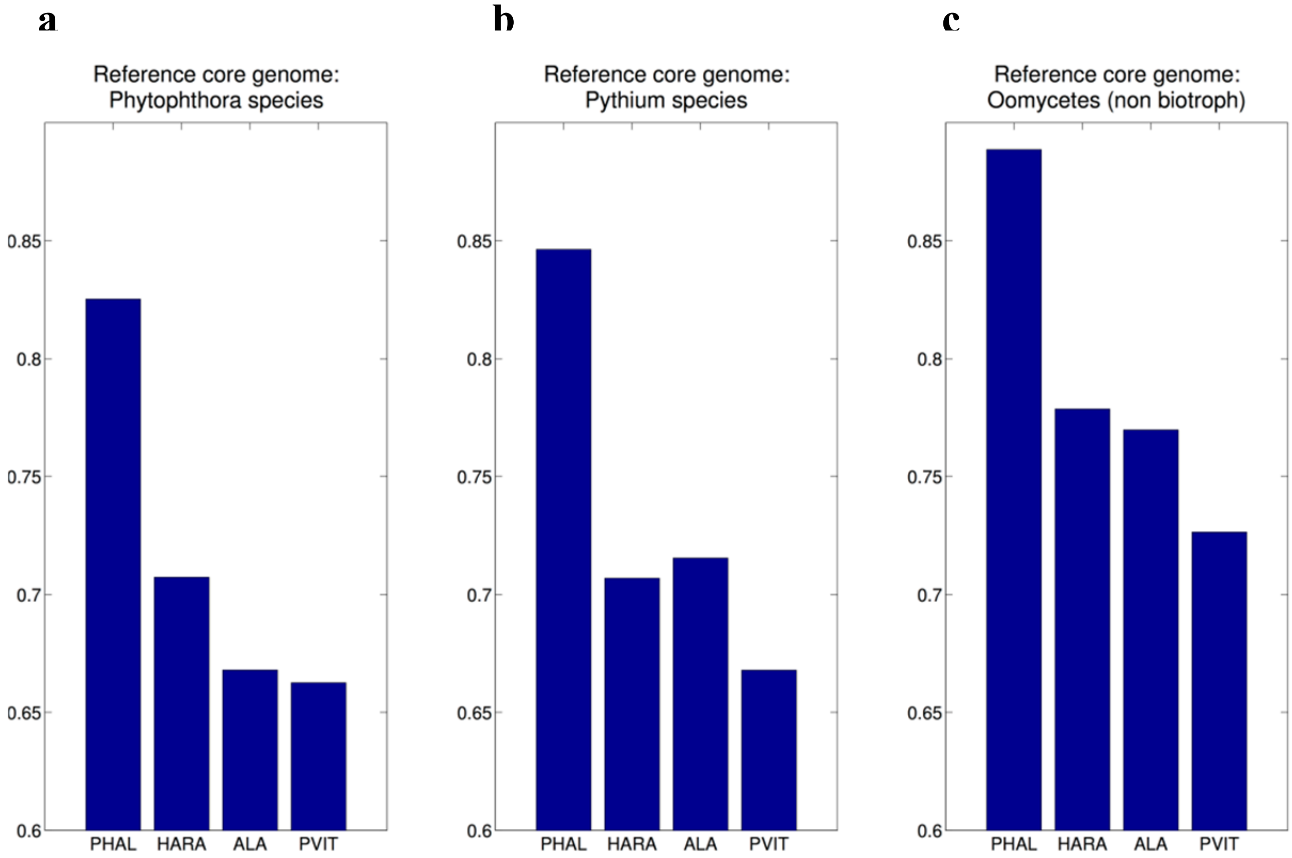

Supplementary Figure S11. The degree of shrinkage of the core genome when one of the biotroph species is added to the genome dataset can give information about the estimated completeness of the *P. viticola* genome.

Y-axis is the size of the core genome when the indicated biotroph is added to the dataset, with respect to the size when it is absent. By definition, the core genome can only decrease when adding more genomes. Since there are four biotrophs in the dataset, we compared the effect of adding one of them with what happens when we add *P. viticola* (PVIT). On the left (**a**) the core genome is calculated for all *Phytophthora* species considered in this work. The middle panel (**b**) shows the same but the reference core genome size is the one from *Pythium* species only. On the right (**c**) the core genome for all the oomycetes (without biotrophs) is used. The addition of the proteome of *H. arabidopsidis* (HARA) or *A. laibachii* (ALA) causes the core proteome size to shrink of about 30-35% with respect to *Phytophthora* and of 30% with respect to *Pythium*. *P. viticola* has a larger effect on the core proteome size. The most complete biotroph genome is the *P. halstedii* (PHAL) one, which seems to indicate that the biotrophs still have 80-85% of the genes present in *Phytophthora* hemi-biotrophic species. This suggests that our draft genome and the ones of *A. laibachii* and *H. arabidopsidis* might miss an additional 20% of the genes.

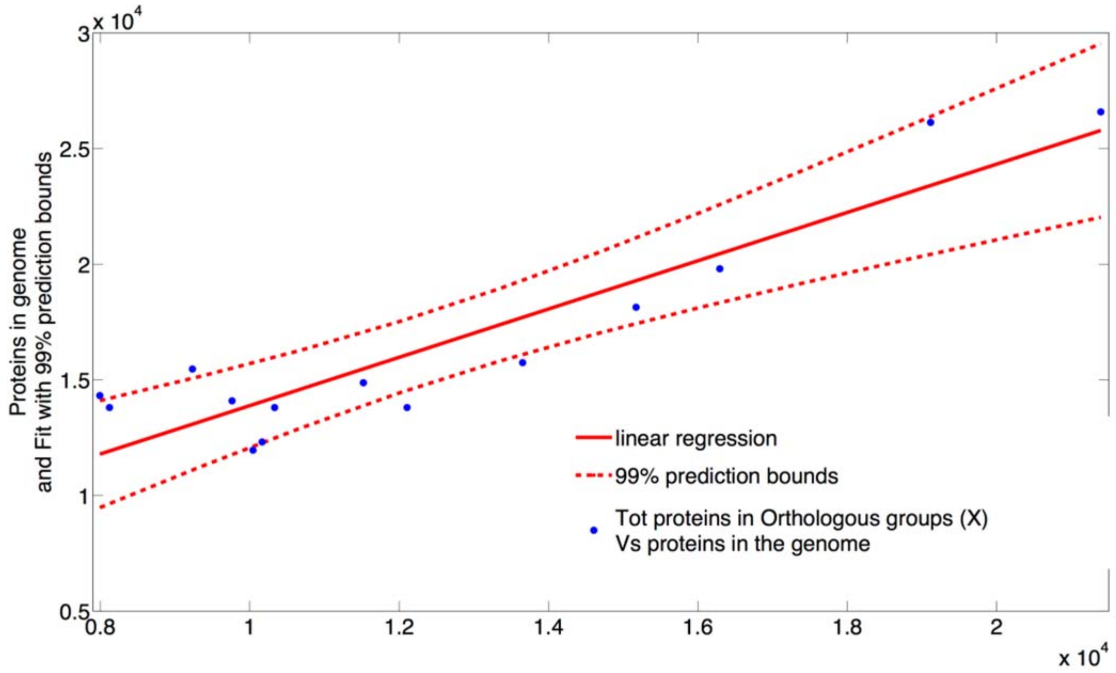

Supplementary Figure S12. An estimate of the "true" protein number in *P. viticola*.

Linear regression of the number of proteins classified into one of the orthologous groups through the Inparanoid analysis, and the size of the full proteome for all oomycetes. The adjusted squared correlation coefficient for the fit is 0.79 when the intercept parameter is set to zero and 0.83 when the intercept is included in the model. *P. viticola* was removed from the analysis and we use the parameters for estimating the likely number of proteins in the *P. viticola* genome.

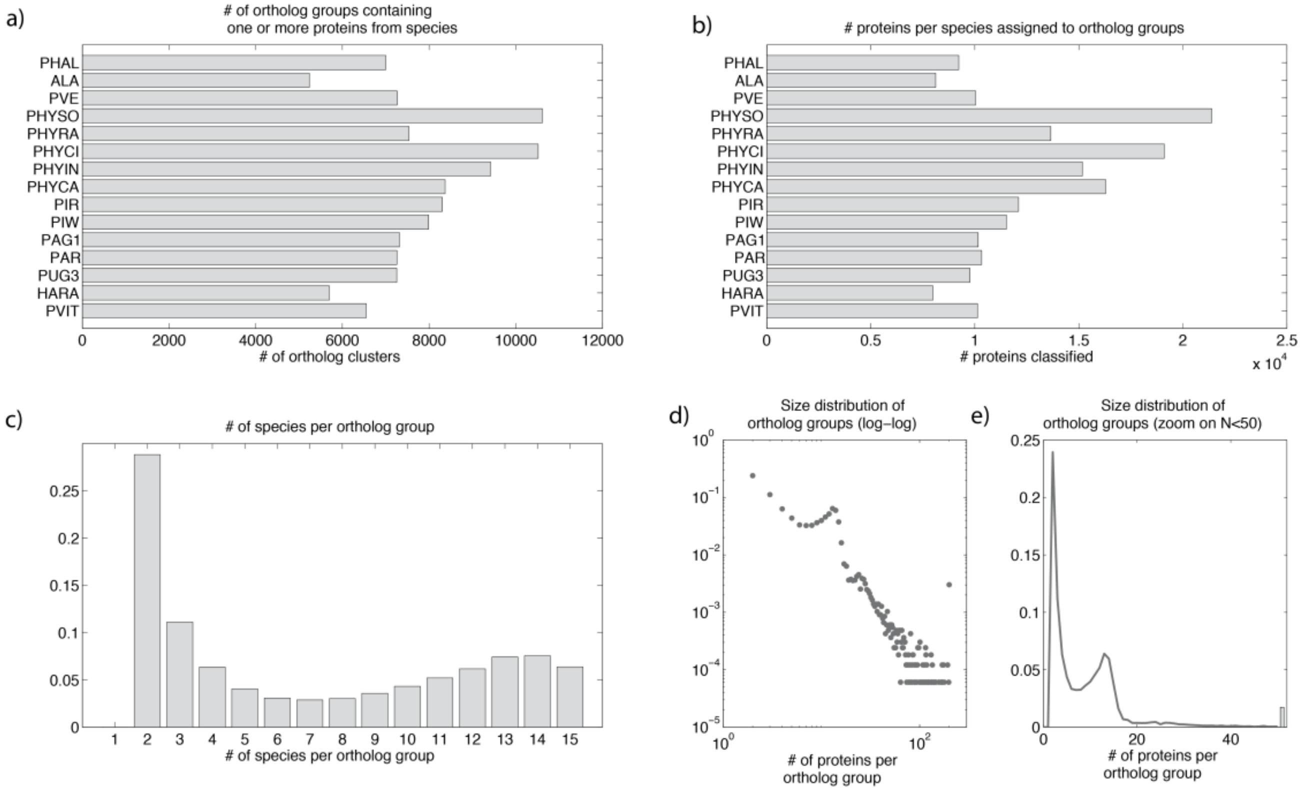

Supplementary Figure S13: Summary of the orthology analysis.

(**a**) Number of orthologous groups (OG) containing the indicated species (PHAL: *Plasmopara halstedii*, ALA: *Albugo laibachii*, PVE: *Pythium vexans*, PHYSO: *Phytophthora sojae*, PHYRA: *Phytophthora ramorum,* PHYCI: *Phytophthora cinnamomi*, PHYIN: *Phytophthora infestans*, PHYCA: *Phytophthora capsici*, PIR: *Pythium irregulare,* PIW: *Pythium iwayamai,* PAG1: *Pythium aphanidermatum,* PAR: *Pythium arrhenomanes,* PUG3: *Pythium ultimum*, HARA: *Hyaloperonospora arabidopsidis,* PVIT: *Plasmopara viticola*). (**b**) Number of proteins of one species assigned to an OG. (**c**) Number of species per OG (for instance category 15 contains the sensu strictu “core” proteins of the Oomycetes). (**d**) Distribution of the number of proteins per OG in log-log scale. (**e**) Detail of the distribution of the number of proteins per OG in the range 2 to 50 proteins. There are 635 groups with more than 30 proteins and 14 with more than 500. The largest OG has 2,632 proteins.

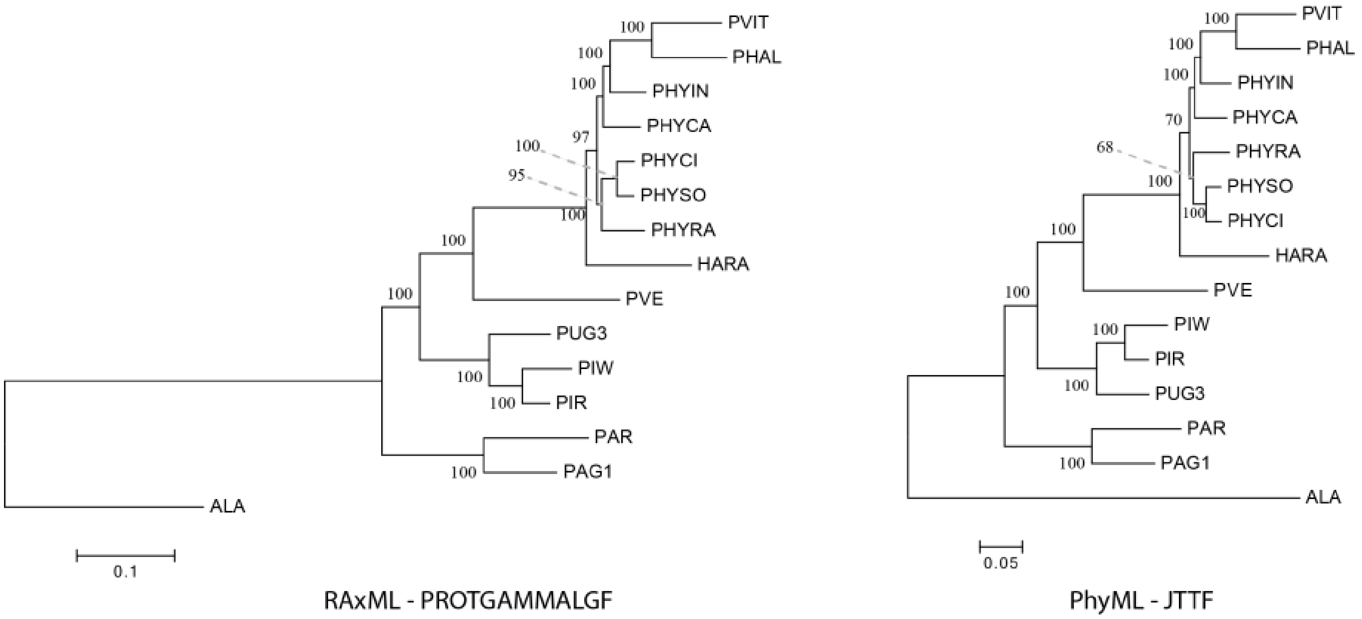

Supplementary Figure S14. Maximum likelihood phylogenetic trees built with RAxML and PhyML.

Evolutionary model is indicated. Equilibrium amino acid frequencies for the model were the empirical ones and rate heterogeneities are accounted for with a gamma distribution whose shape parameter was calculated during the phylogenetic reconstruction process. (PHAL: *Plasmopara halstedii*, ALA: *Albugo laibachii*, PVE: *Pythium vexans*, PHYSO: *Phytophthora sojae*, PHYRA: *Phytophthora ramorum,* PHYCI: *Phytophthora cinnamomi*, PHYIN: *Phytophthora infestans*, PHYCA: *Phytophthora capsici*, PIR: *Pythium irregulare,* PIW: *Pythium iwayamai,* PAG1: *Pythium aphanidermatum,* PAR: *Pythium arrhenomanes,* PUG3*:* *Pythium ultimum*, HARA: *Hyaloperonospora arabidopsidis,* PVIT: *Plasmopara viticola*).

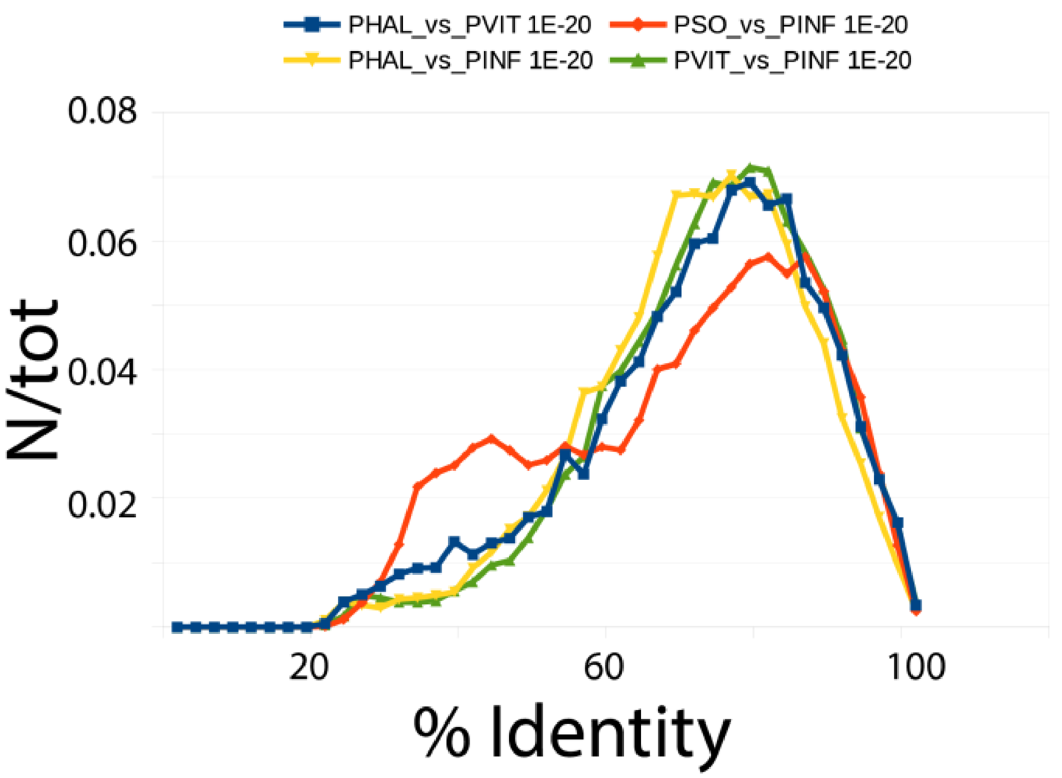

Supplementary Figure S15. Full proteome comparisons.

The identity percentage of the first blast hit for whole proteome comparisons performed using blastp (--max_target_seqs=1) is plotted. The blast output was filtered at e-value≤ e^{-20} and a histogram was built with the threshold passing alignments. To better appreciate the similarity among the two *Plasmopara* species (PHAL, PVIT) and *Phytophthora infestans* (PINF), we also performed the analysis for the latter vs *Phytophthora sojae* (PSO). The comparison of the two *Plasmopara* is not different from the comparisons *Plasmopara-Phytophthora infestans*.

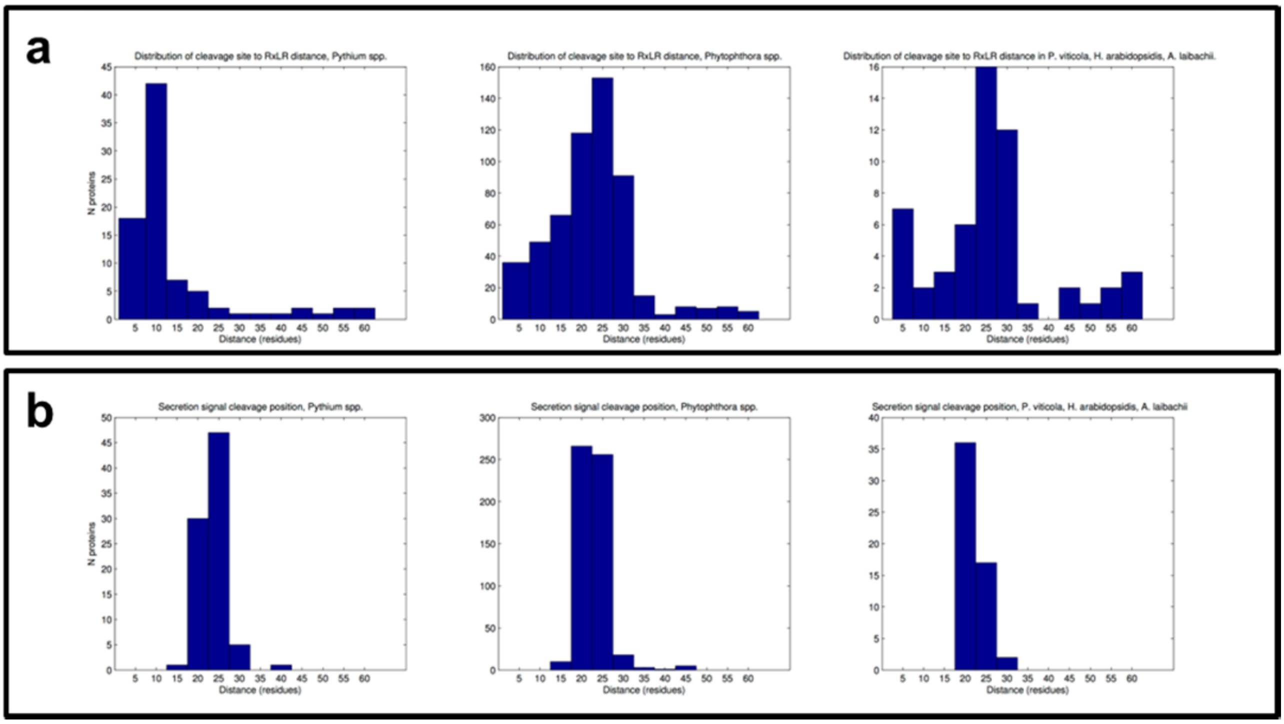

Supplementary Figure S16. Distribution of the distance and position of the signal peptide cleavage site in oomycete species.

(a) Distribution of the distance dividing the signal peptide cleavage site, as predicted by Signal-P[39] and the RxLR occurrence identified through match to the regular expression. (**b**) Distribution of the position of the secretion signal peptide cleavage site for proteins in (**a**) Data for *Pythium, Phytophthora* spp. and the biotrophs *P. viticola*, *H. arabidopsidis* and *A. laibachii* are indicated in the left, middle and right columns, respectively. A shorter distance between the cleavage site and the RxLR motif is specific to *Pythium* species suggesting differences in the machinery or the secretion system.

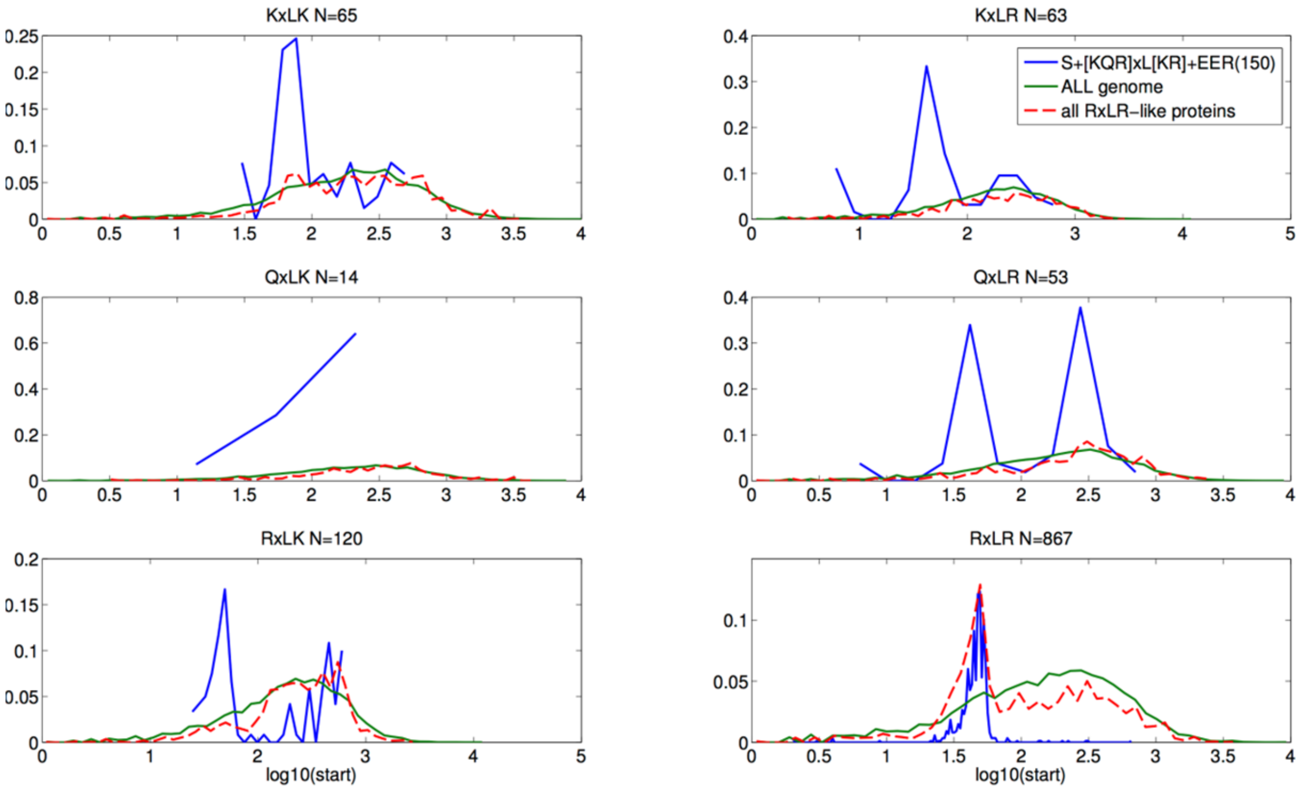

Supplementary Figure S17. As expected, some of the RxLR variants also have a biased localization towards the N-terminal of the proteins.

In the title of each plot we indicate the motif and the number of occurrences over all the RxLR-like. When plotting the start position of motifs found over the whole RxLR-like dataset we recover the background distribution (compare dashed red with green), in agreement with the presence of a lot of false positive RxLR through the homology search only. When adding constrains for selecting effector proteins we consistently reduce the number of candidates and we select proteins with a biased localization of the motifs, indicating a functional role for the motif. No motif has however a bias as strong as the canonical RxLR motif. Abundances were divided by the total in each data series.

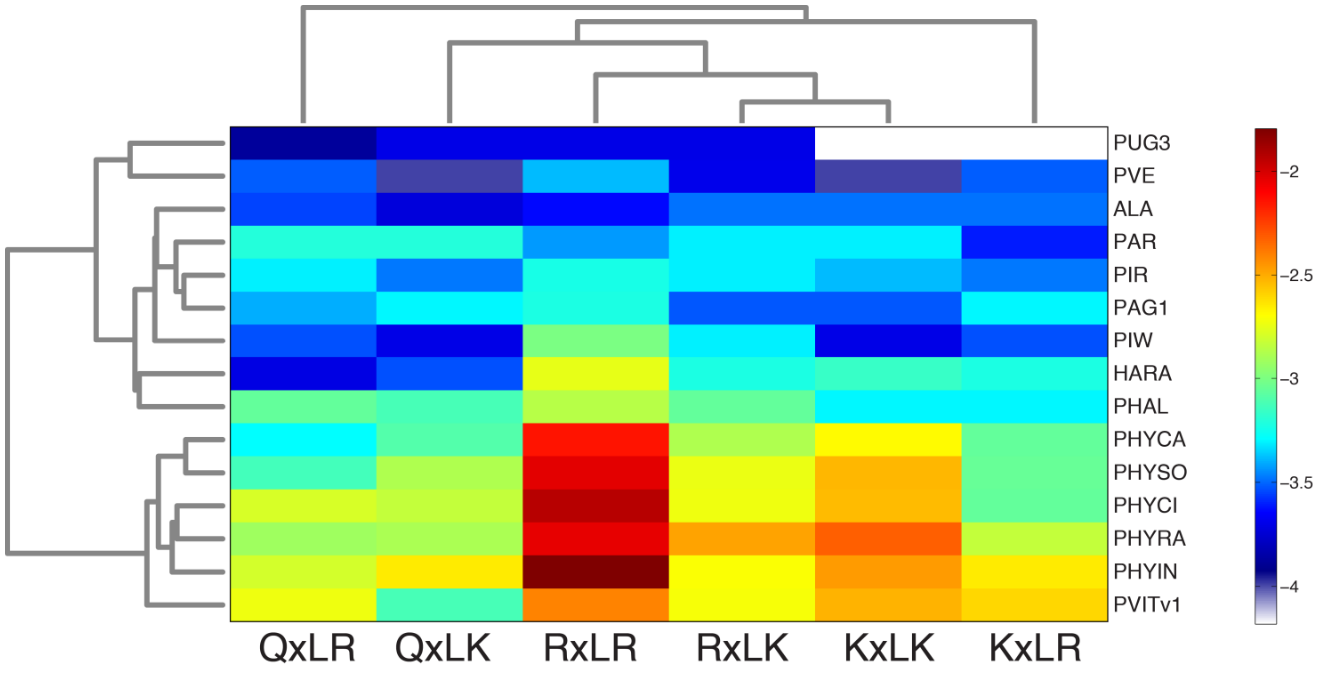

Supplementary Figure S18. Clustering of the motif abundances for RxLR-like where the occurrence of the motif is associated to a signal peptide prediction (with no constrains on their relative position or the presence of additional motifs).

We normalized the counts for an organism by the total number N of proteins assigned to orthologous groups by Inparanoid for that organism. We applied this normalization because it is much more robust to differences in the gene prediction methods than the raw number of proteins in a draft genome that can be partial or comprise many putative proteins, as in the *P. viticola* case (**see Supplementary Fig. S12**). Plotted values are log10 of the ratio among the motif counts and N, standardized by row. The clustering of the rows defines two groups: RxLR-rich and RxLR-poor organisms, respectively. In more or less all organisms the preferred motif is the RxLR, but other motifs can be abundant as well, especially in organisms where this is not expected, as the *Phytophthora*, where the focus has been mainly on RxLR proteins and nothing is known about the putative effectors with variant motifs. The two *Plasmopara* species behave quite differently. *P. viticola* is placed in the RxLR-rich group; *P. halstedii* is instead placed in the effector-poor group, mainly for the presence of a small number of canonical RxLR. Distance used for the clustering was the Euclidean, single-linkage clustering method. (PHAL: *Plasmopara halstedii*, ALA: *Albugo laibachii*, PVE: *Pythium vexans*, PHYSO: *Phytophthora sojae*, PHYRA: *Phytophthora ramorum,* PHYCI: *Phytophthora cinnamomi*, PHYIN: *Phytophthora infestans*, PHYCA: *Phytophthora capsici*, PIR: *Pythium irregulare,* PIW: *Pythium iwayamai,* PAG1: *Pythium aphanidermatum,* PAR: *Pythium arrhenomanes,* PUG3*: Pythium ultimum*, HARA: *Hyaloperonospora arabidopsidis,* PVIT: *Plasmopara viticola*).

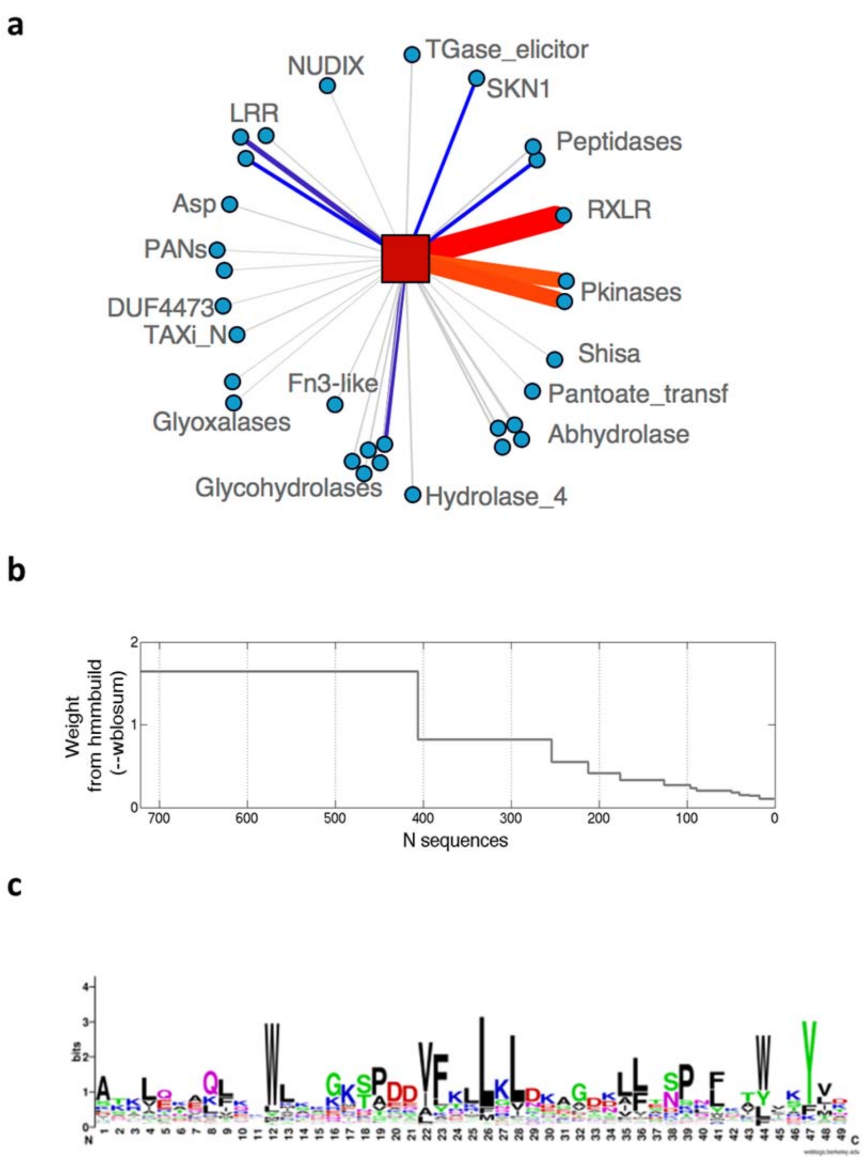

Supplementary Figure S19. Graphical representations of the occurrences of Pfam domains in RxLR-like proteins.

A total of 1,182 RxLR-like proteins possessing a signal peptide and the variants associated to an EER were considered (**Supplementary Table S10**) and indicated in (**a**) with a red square. Only 387 proteins have a hit in the Pfam database at the threshold used (e-value≤0.0001), confirming that most of the putative RxLR proteins have no known domains. Pfam does not contain a model for the well-known WY domain of RxLR proteins. We built it from 721 WY domains annotated in Boutemy *et al.*[40] by using hmmbuild and weighting sequences using the Henikoff simple filter (--wblosum)(**b**). The corresponding sequence logo is shown in (**c**). We found that the WY domain is present in 368 sequences out of 721 (e-value≤0.0001). The RXLR_pfam (PF16810) domain is able to recover only 70 of these proteins. The input regexp-defined RxLR from *Phytophthora* is much higher, therefore the RxLR HMM is not able to detect a significant number of RxLR proteins, even in the genome of species used to build the model. This confirms that the RxLR are rapidly evolving, both in terms of sequence divergence and of domain arrangements.

a

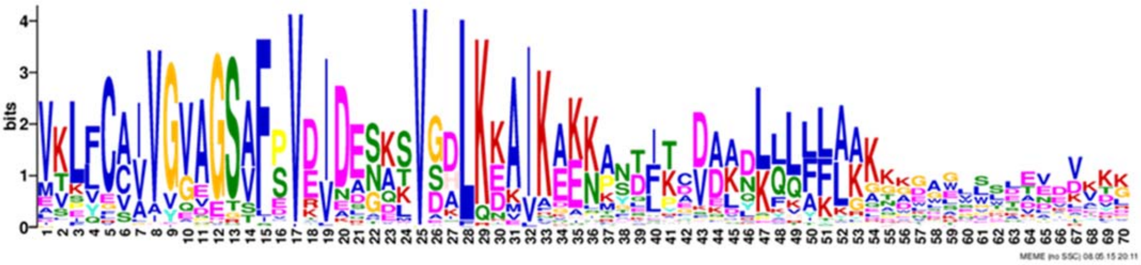

b

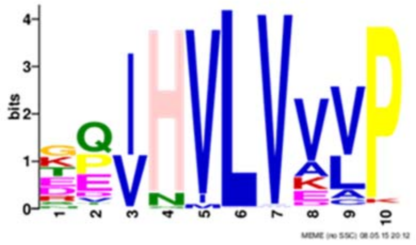

Supplementary Figure S20. Sequence logo of the LFLAK domain and VVP motif of CRN proteins

(**a**) MEME was run on a set of 430 CRN proteins as annotated by Haas *et al.* [5]. Conservation of the LFLAK motif appears lower than what shown in Haas *et al.* [5] because the present analysis included 430 unaligned *bona fide* Crinkler proteins while only 16 were considered in previous studies. (**b**) Sequence logo of the VVP motif in Crinkler sequences.

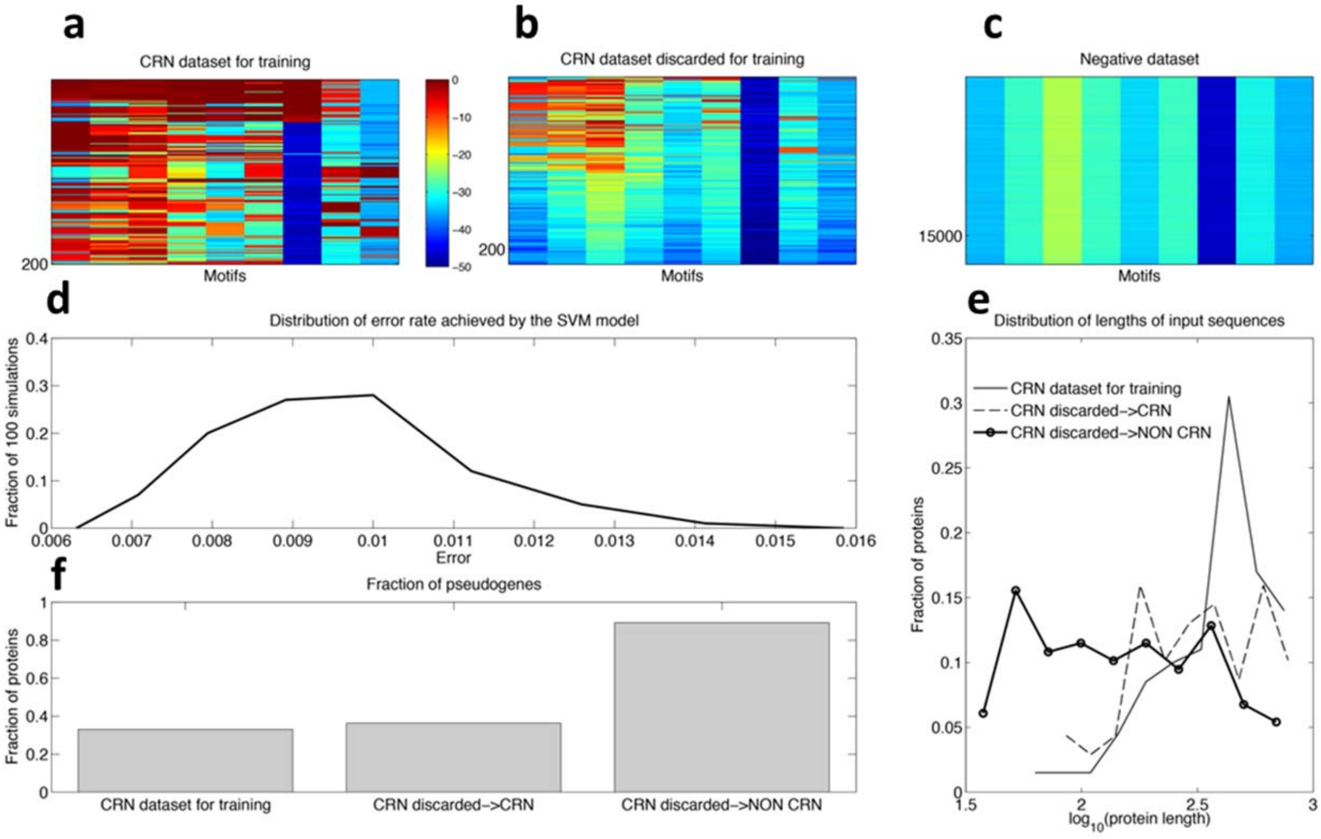

Supplementary Figure S21. Crinkler classification using SVM.

a, **b** and **c** are the input log10 probability matrices for motifs. Each column corresponds to a motif of length 30 found by MEME. Each row corresponds to a *P. infestans* protein sequence. **d**) The error distribution was estimated using 100 cross validations for each of 100 different training sets, as explained in the supplementary note. The SVM model returned a wrong classification for most of the CRN not used for training. We explored this issue in more detail and we concluded that their classification is very difficult because they are often pseudogenes missing portions of the sequence containing the motifs used for the classification. **e**) length distributions of the proteins from the starting dataset of Crinkler proteins, partitioned into three classes: the 200 proteins used for training the models, the 69 Crinkler proteins not used for training and correctly classified by the SVM model and the 148 Crinkler proteins missed by the algorithm. Proteins with wrong classification by the SVM tend to be shorter than the remaining and they likely miss motifs/domains that might be important for classification. **f**) The fraction of proteins annotated as pseudogenes in the three groups defined above, showing that over 80% of the proteins of the non-identified CRNs are indeed annotated as pseudogenes.

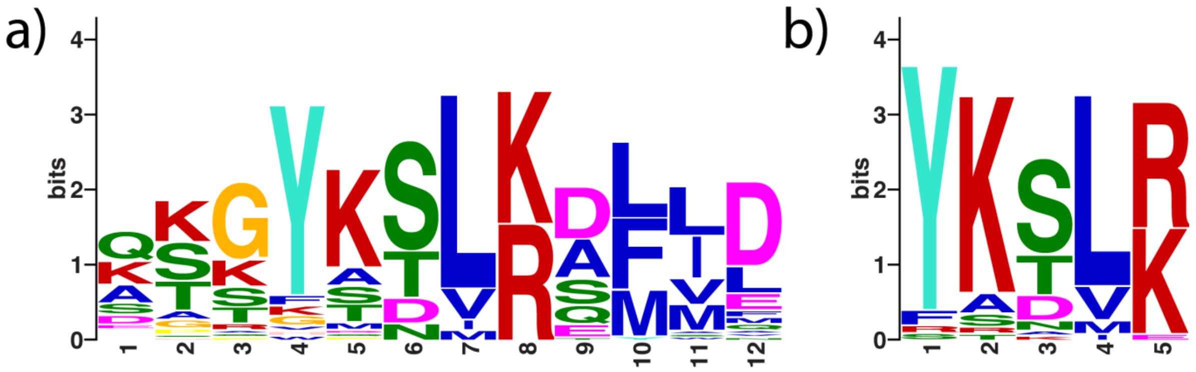

Supplementary Figure S22. Sequence logo of the YxSLK motif found by MEME in all *Pythium ultimum* "*Family 3*" proteins.

The surrounding region is also shown. **a**) When asking MEME for motifs ranging from 4 to 12 residues (ranking 6th). **b**) when asking MEME for motifs of width exactly 5 residues (ranking 9th). Sequence logos were produced by MEME [30].

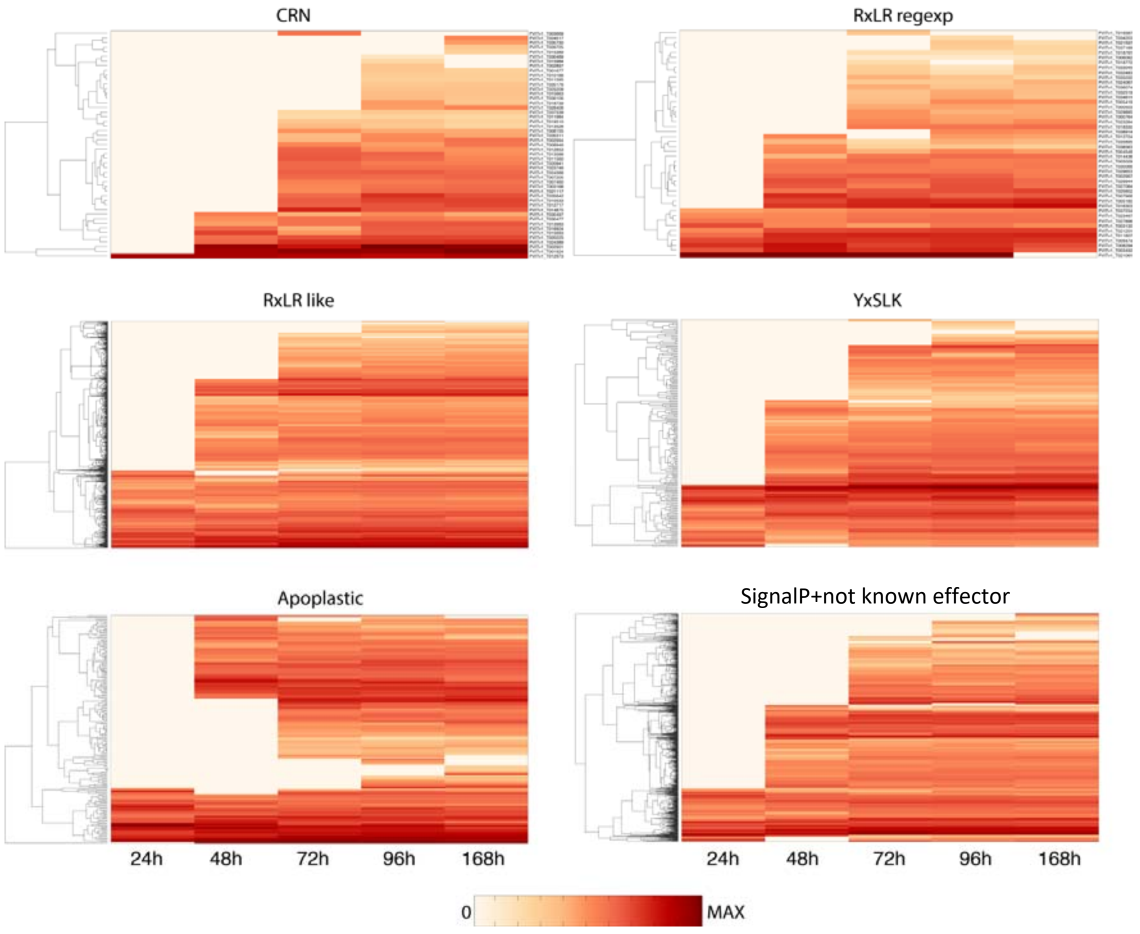

Supplementary Figure S23: Hierarchical clustering of effector gene expression profiles

Only transcripts for which we detected expression in at least one time point were plotted. Expression values in FPKM were log10 transformed.

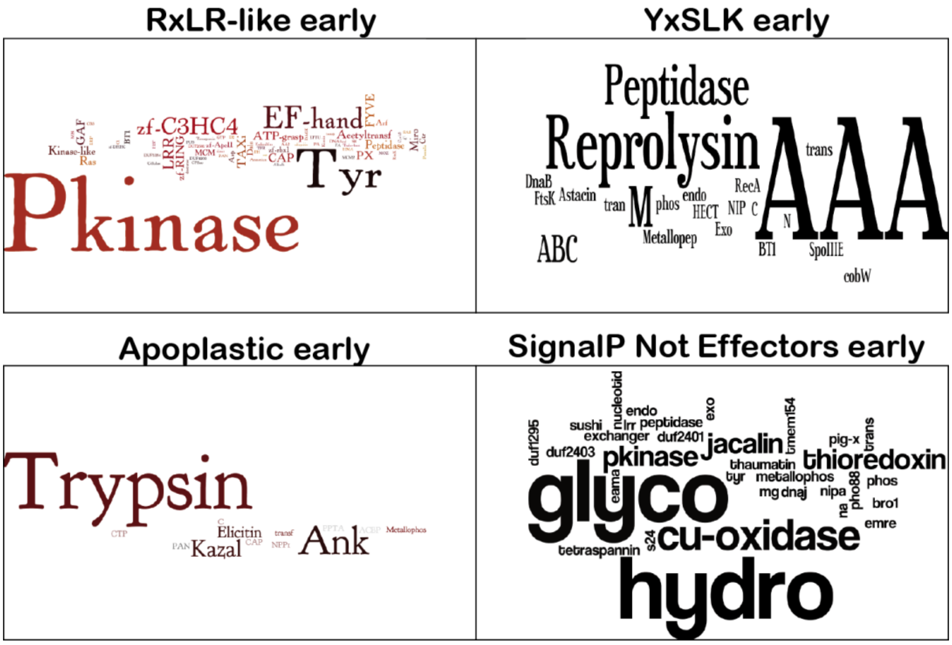

Supplementary Figure S24*.* Word cloud highlighting recurrent terms in protein domains found in *Plasmopara viticola* apoplastic, YxSLK and secreted proteins.

P. viticola genes having their maximum expression level at 24 hpi were searched against the pfamA database using hmmscan (using default parameters). The Pfam models with a significant hit (at domain e-value≤0.0001) were used to build a word cloud to highlight the recurrent terms. For similar Pfam accessions, we remove the numeric code. Hydrolase and peptidase (trypsin, peptidase, reprolysin, Cu-oxidase, astacin…) functions are over-represented in the apoplastic, YxSLK and secreted proteins. All of these evidences indicate that *P. viticola* makes heavy deployment of degradative enzymes at the very beginning of the infection. The RxLR-like group is instead enriched in Protein kinase activity therefore suggesting that *P. viticola* also interfere with phosphorylation (cascades) in the plants.

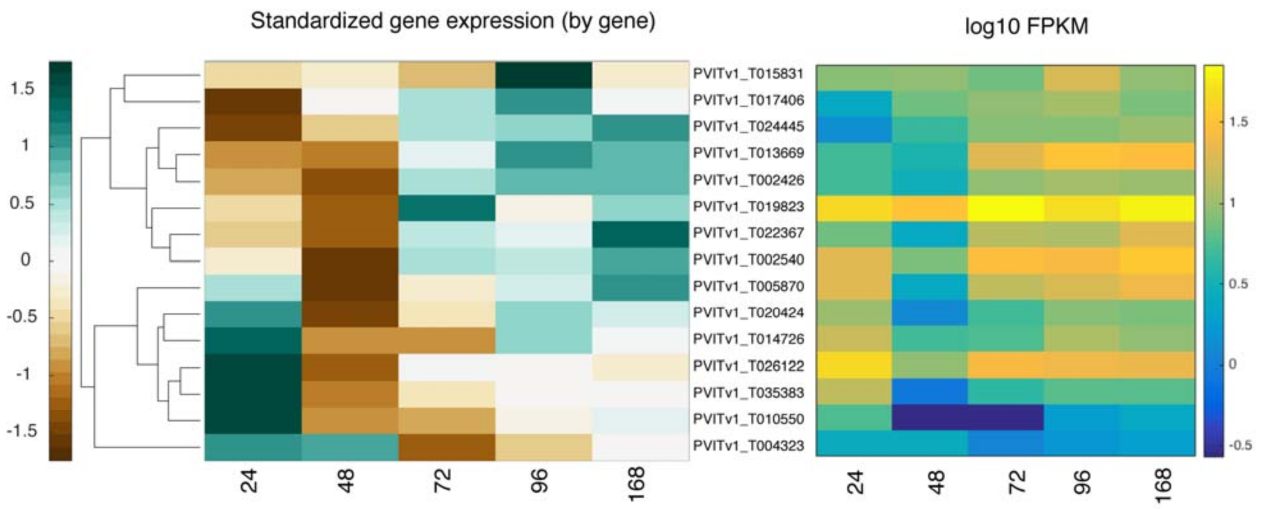

Supplementary Figure S25. Differential expression of 15 genes with a significant hit for the LRR Pfam model. On the left the row standardized matrix used for building the dendrogram, and on the right the actual FPKM values.

15 of the 111 genes with a significant hit for the LRR Pfam model in *P. viticola* are expressed at 24 hpi after infection and some of them have their maximum expression level at this stage. Two groups of sequences expressed at 24 hpi emerges: one with expression peak at 24 hpi that then are down regulated, and one with delayed expression that increases after 48 hpi.

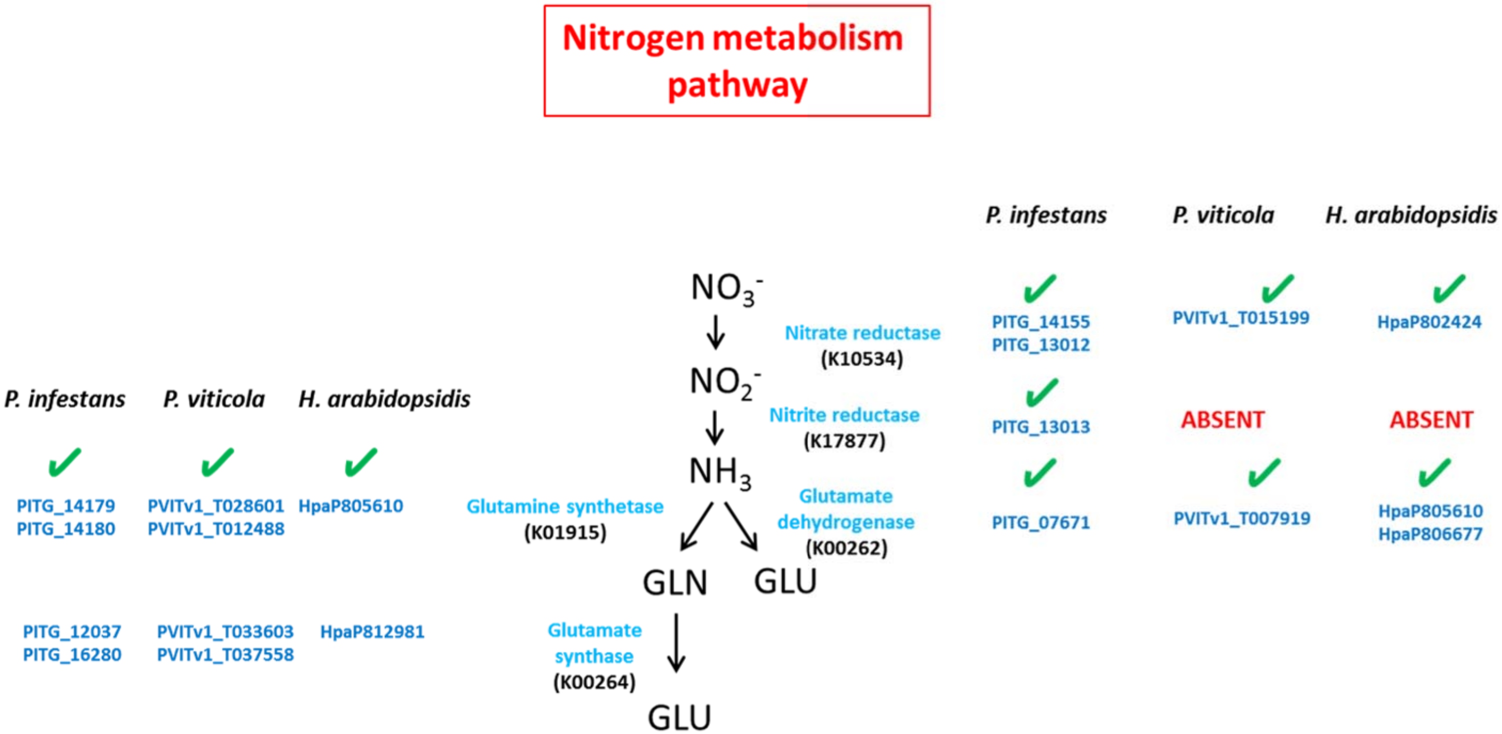

Supplementary Figure S26. Nitrogen metabolism pathway in *P. viticola.*

The gene ID number for the enzymes in this pathway in *P. viticola, P. infestans* (PITG), and *H. arabidopsidis* (Hpa) genomes are indicated. They are considered absent if not found in the respective genome assemblies.

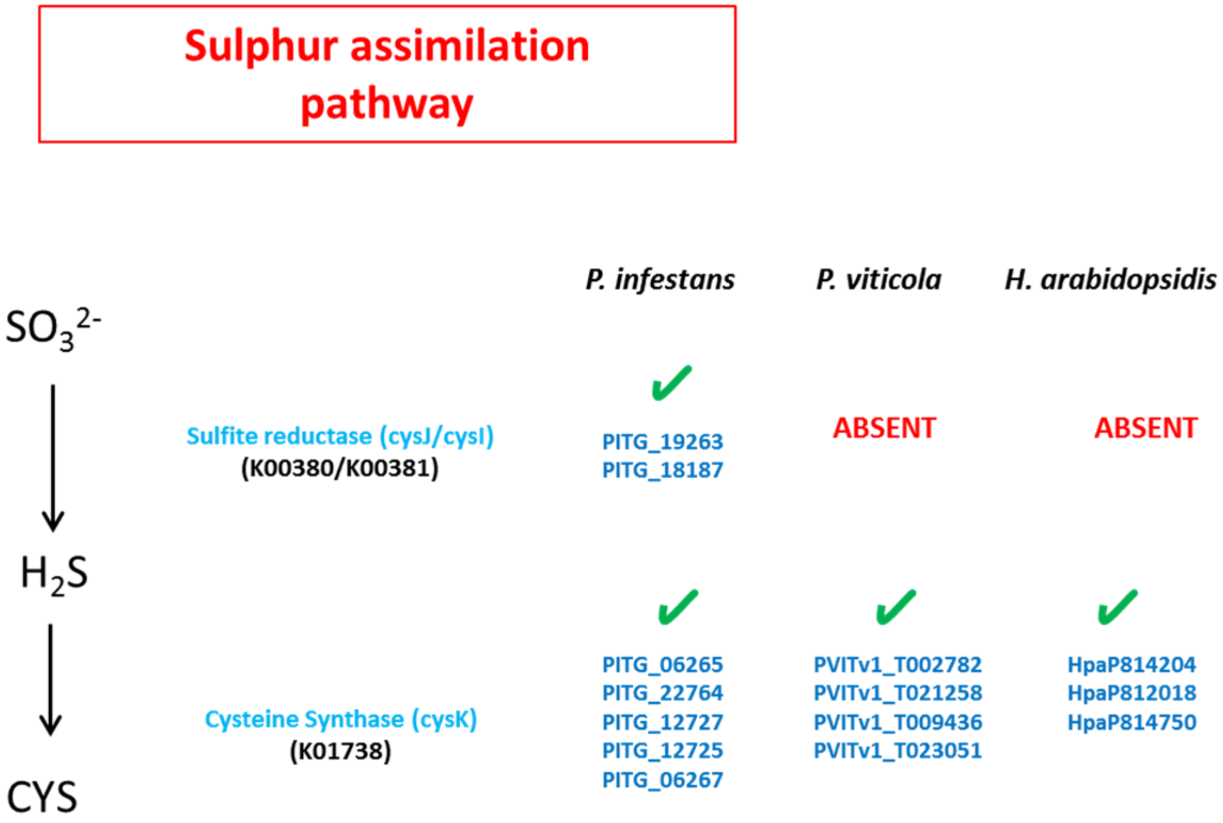

Supplementary Figure S27. Sulphur assimilation pathway in *P. viticola.*

The gene ID number for the enzymes in this pathway in *P. viticola, P. infestans* (PITG), and *H. arabidopsidis* (Hpa) genomes are indicated. They are considered absent if not found in the respective genome assemblies.

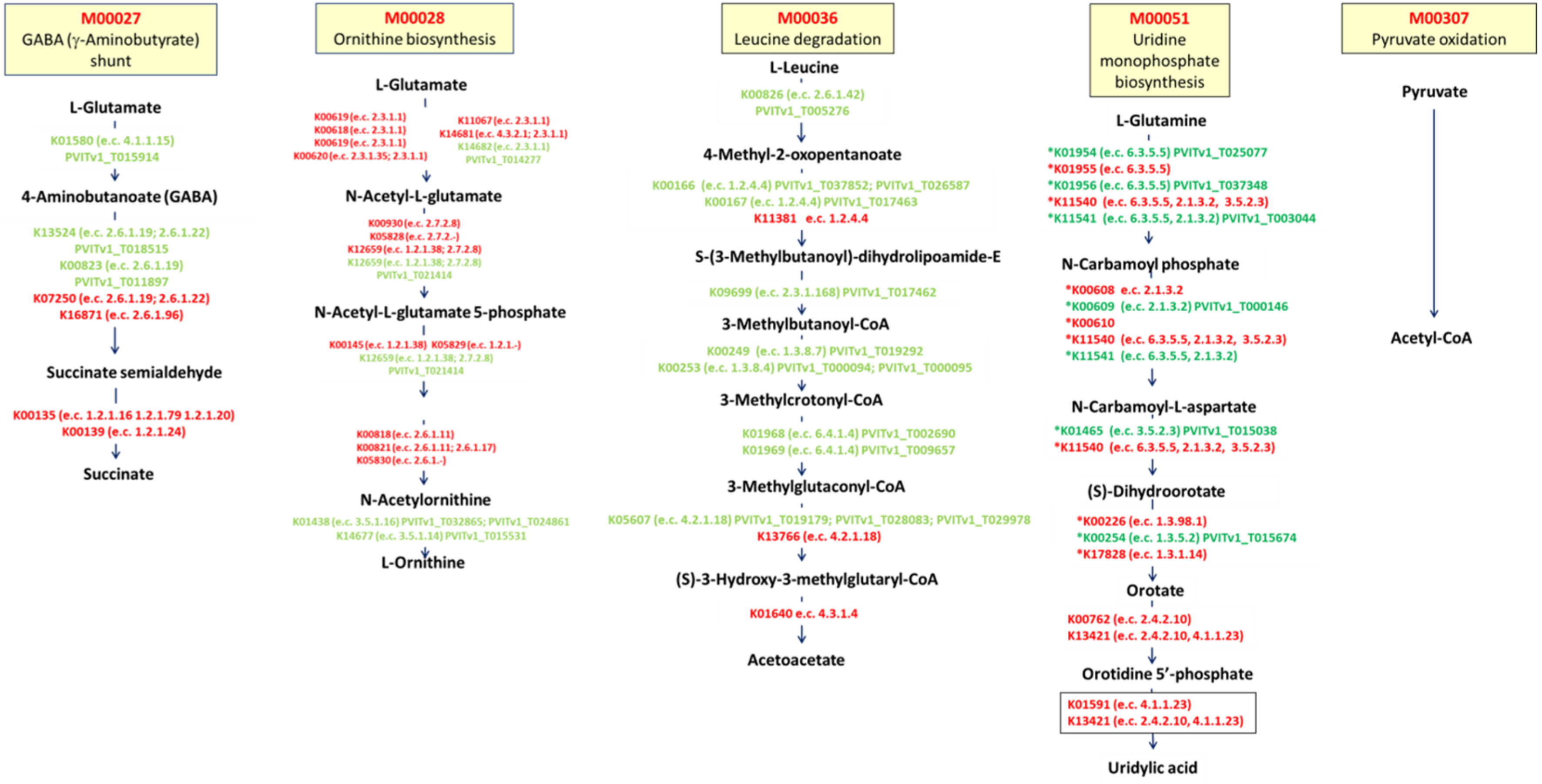

Supplementary Figure S28. Incomplete metabolic modules in *P. viticola.*

The enzymes present in *P. viticola* genome and the corresponding gene numbers are indicated in green. The enzymes missing are indicated in red.

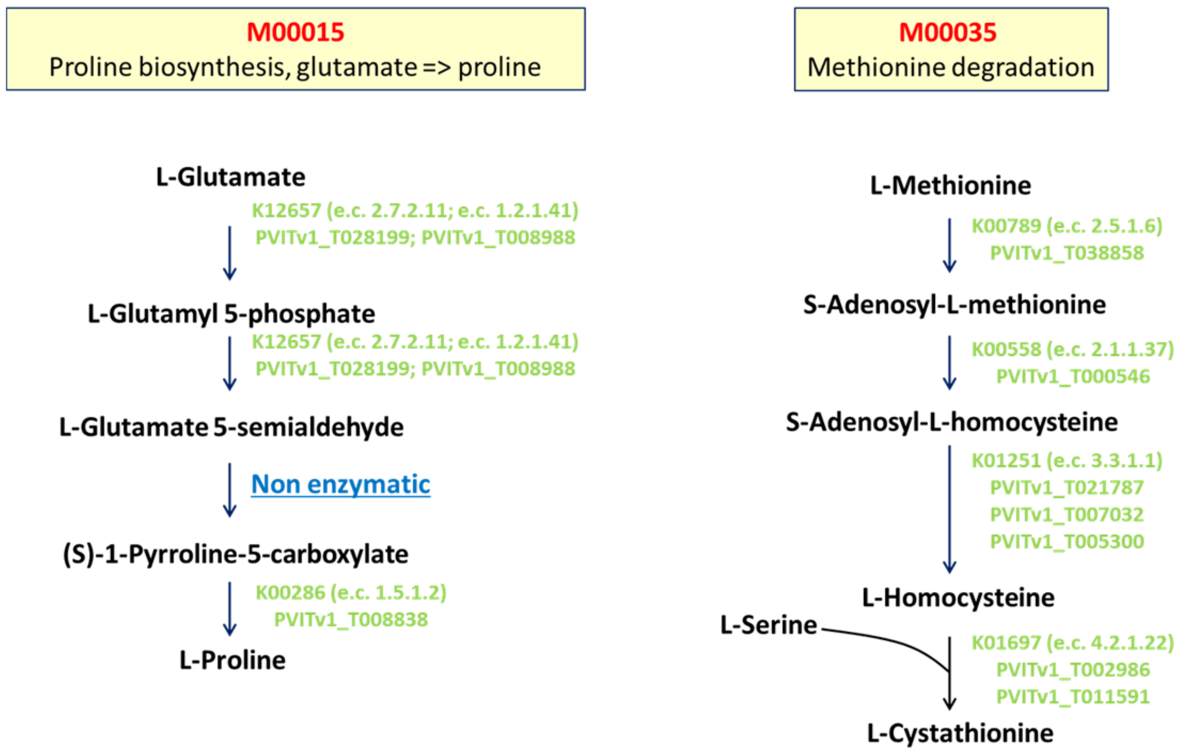

Supplementary Figure S29. Metabolic modules found in *P. viticola* but missing in *H. arabidopsidis* and *P. infestans.*

The enzymes present in *P. viticola* genome and the corresponding gene numbers are indicated in green.

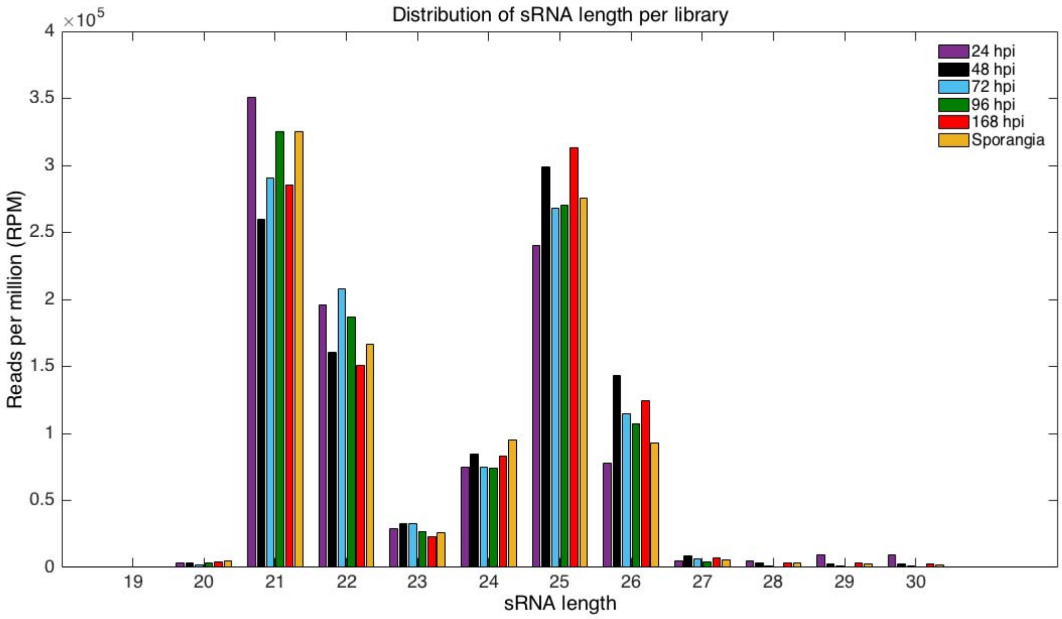

Supplementary Figure S30. Normalized (RPM) *P. viticola* small RNA read counts per library.

The normalized values in RPM were calculated from raw counts by multiplying raw counts by $10^6/N$ were N is the total number of reads mapped on the *P. viticola* genome from the same library.

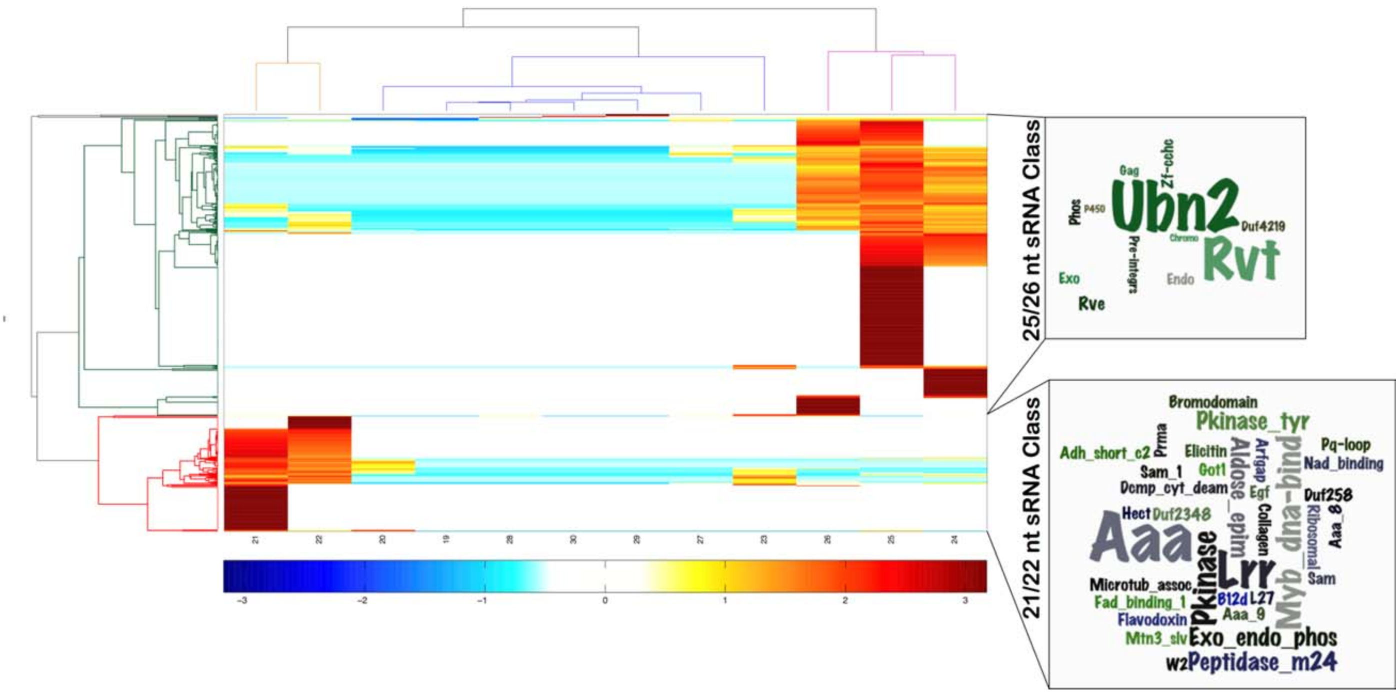

Supplementary Figure S31. General functions of genes generating sRNAs of different lengths in *P. viticola*.

Two well-defined groups of sequences are defined: one (highlighted in green) containing genes mainly associated with sRNAs of 24 to 26 nucleotides and another (in red) containing genes mainly associated with 21-22 nt long sRNAs. Pfam domains associated to the proteins in the clusters were retrieved and the list obtained was used to generate the word cloud using genes2WordCloud (http://www.maayanlab.net/). The green group is strongly enriched in Ubn2 (gag-polypeptide of LTR copia-type) and Rvt (Reverse transcriptase (RNA-dependent DNA polymerase)), while the red one is more heterogeneous and contains several different domains not related to mobile elements.

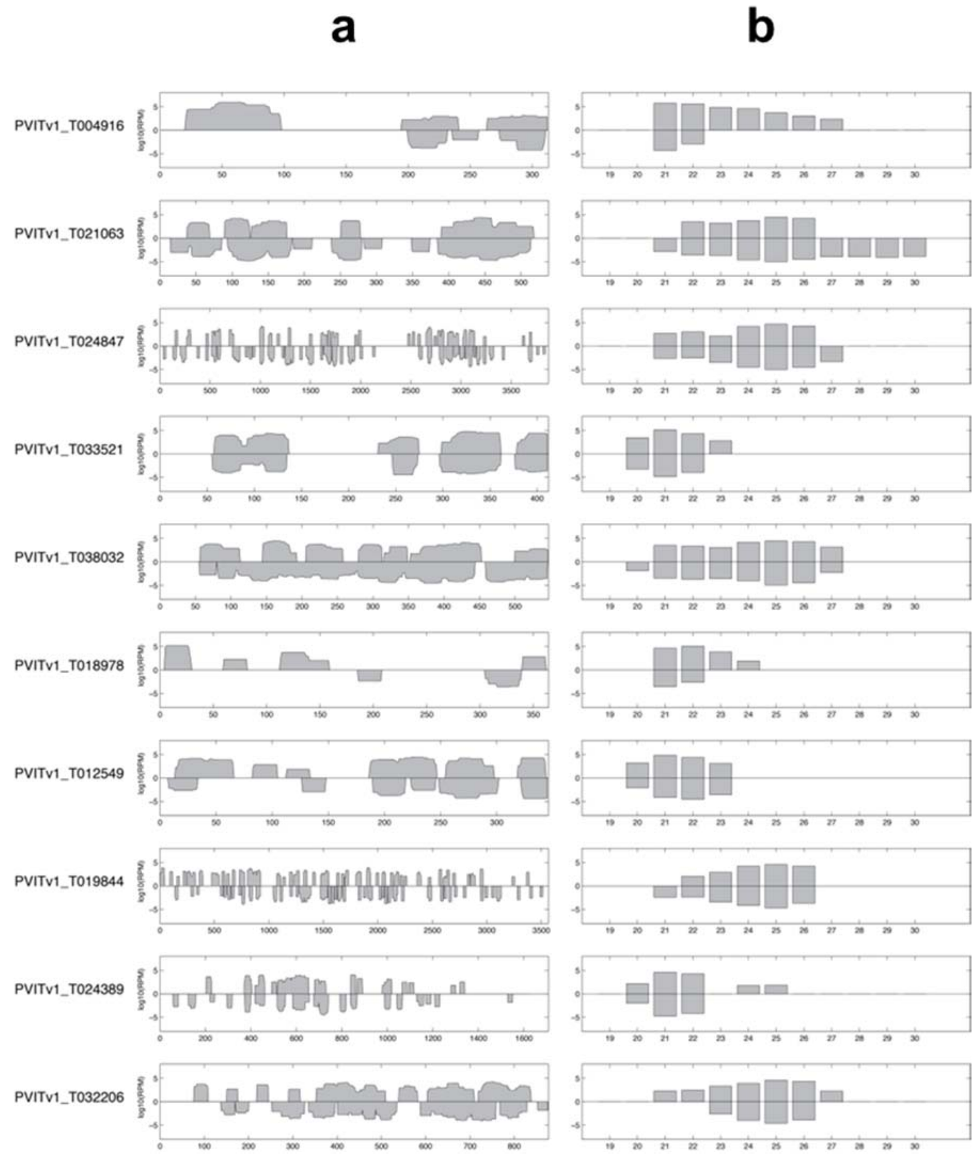

Supplementary Figure S32*:* Secondary sRNA coverage and length distribution plot of ten *P. viticola* genes producing sRNAs*.*

Plots in (**a**) correspond to the secondary sRNA coverage, expressed as log10 of the RPMs for a certain nucleotide position, after summing the abundances of sRNAs of all lengths. Counts from different libraries were normalized to RPM values and then all RPMs were summed to give the plotted values. The RPMs of reads mapping on the complementary strand of the transcript are plotted after reversing the sign of the abundances. Plots in (**b**) are the length distributions of the corresponding sRNAs. sRNAs mapping on the negative strand are placed below the x-axis. PVITv1_T004916, T024389 and T012549 have been previously identified as CRN effectors, which are confirmed as strong secondary sRNA producers, similar to what has been observed in *P. infestans*[41]. PVITv1_T019844 is a pol-like protein, therefore related to transposable elements. The presence of sRNAs originating from both strands strongly suggests that these transcripts are first processed by RNA-dependent RNA polymerases and then likely sliced by a Dicer-like mechanism.

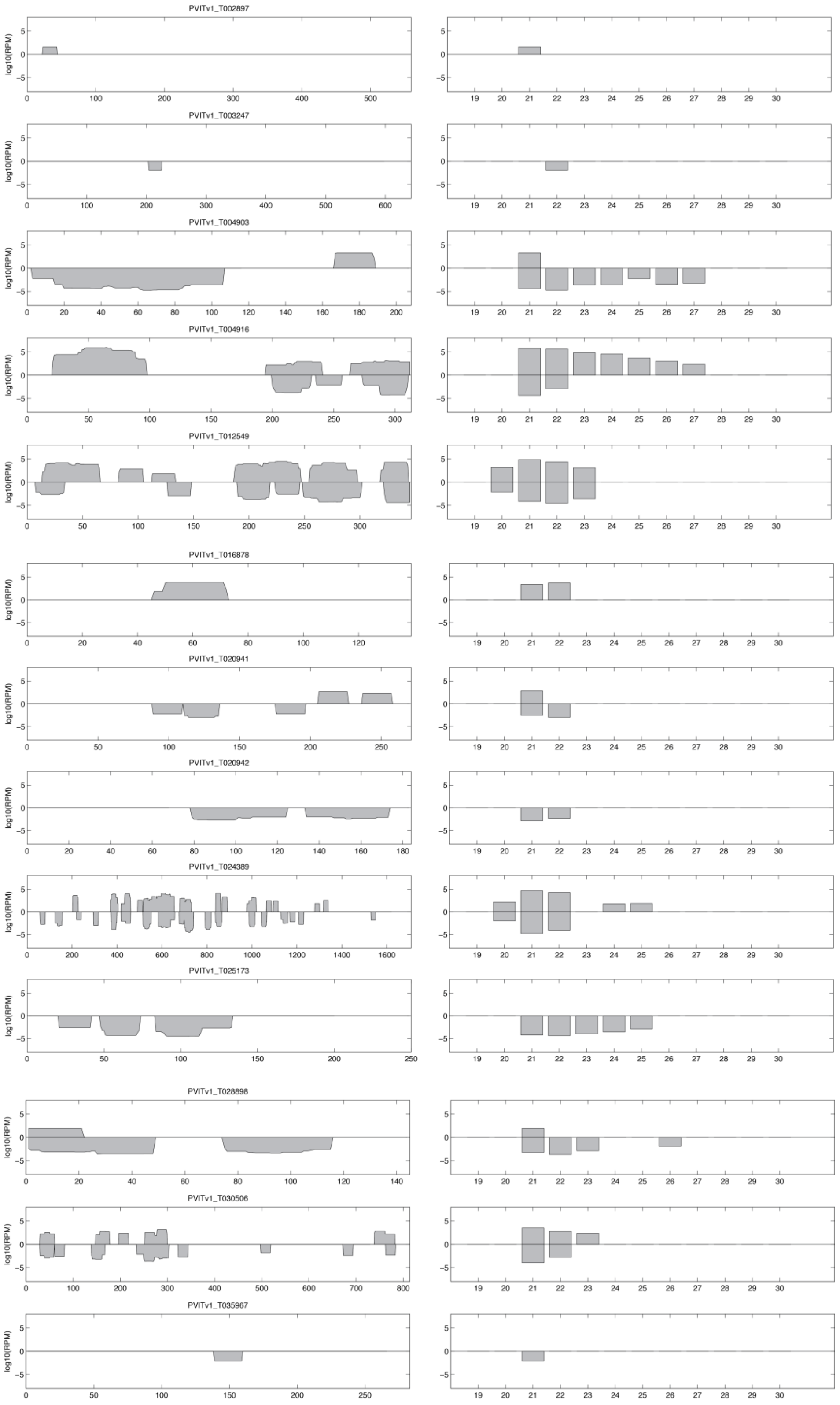

Supplementary Figure S33. Secondary sRNA coverage and length distribution of sRNAs produced by CRN genes.

The Crinkler genes are mostly associated with 21/22 nt long sRNAs. The secondary sRNA coverage is indicated on left panels, the length distributions on the right.

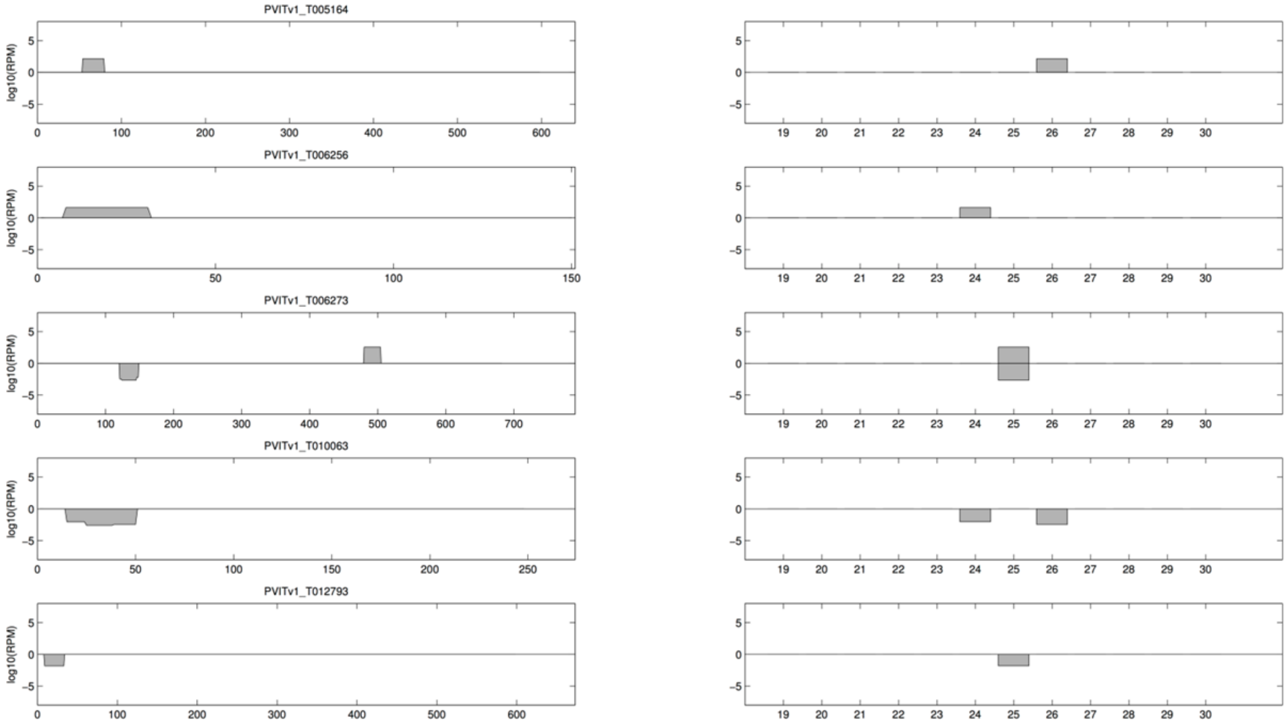

Supplementary Figure S34. Abundance and length distribution of secondary sRNAs produced by YxSLK effector genes*.*

The sRNA coverage is indicated on left panels, the length distributions on the right.

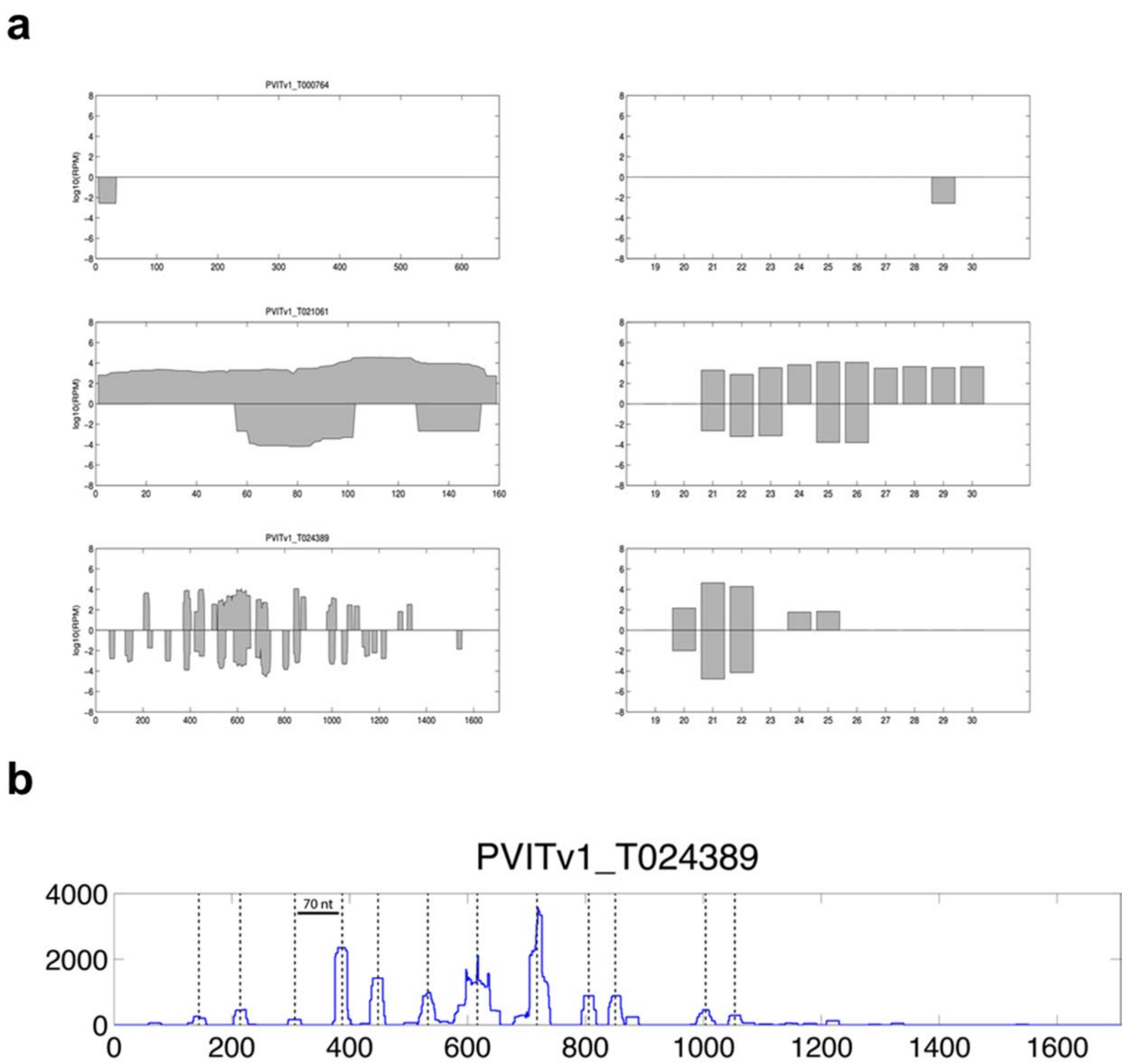

Supplementary Figure S35. sRNA coverage and length distribution of secondary sRNAs produced by RxLR effector genes.

The sRNA coverage is indicated on left panels, the length distributions on the right for the RxLR effector genes (**a**). PVITv1_T024389 is an RXLR-like and the same time also a CRN, and has a peculiar pattern of sRNAs, almost only of 21/22 nt, with a periodicity of 40-60 nucleotides between consecutive peaks (**b**).

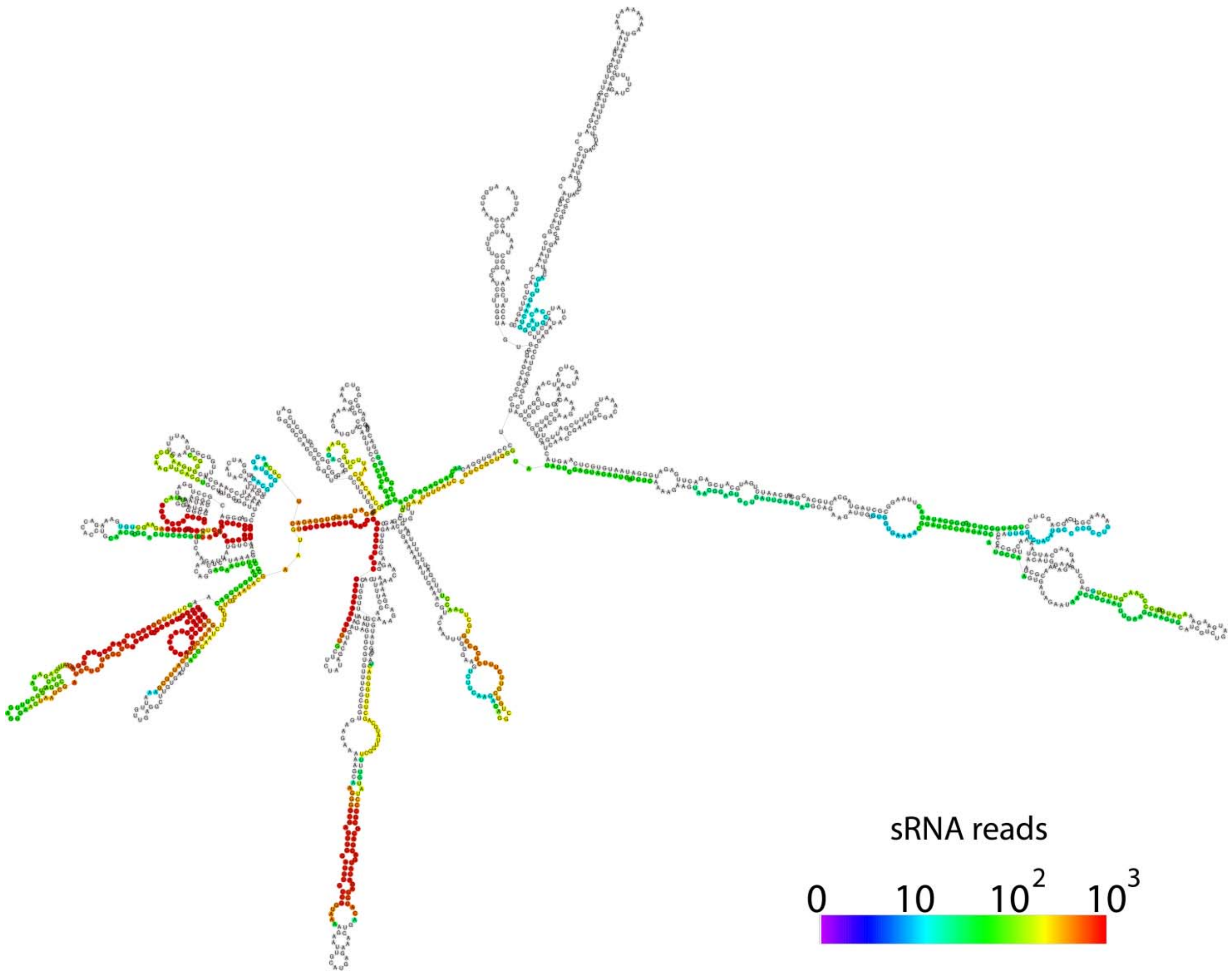

Supplementary Figure S36. RNA secondary structure of PVITv1_T024389.

The hairpins were colored to indicate the sRNA coverage expressed as raw number of reads in all libraries. The secondary structure shows similarities to polycistronic sRNA precursor.

Supplementary Tables

Supplementary Table S1. See attached Excel file "GO annotation of *P. viticola* proteome".

Supplementary Table S2. Evaluation of performances of the preliminary and final Augustus parameters.

Objective	Sensitivity	Specificity
**Preliminary**		
Nucleotide (multi)	0.97	0.745
Exon (multi)	0.87	0.68
Gene (multi)	0.755	0.465
Nucleotide (mono)	0.999	0.826
Exon (mono)	0.959	0.497
Gene (mono)	0.94	0.61
**Final**		
Nucleotide (multi)	0.981	0.835
Exon (multi)	0.838	0.713
Gene (multi)	0.604	0.43
Nucleotide (mono)	0.993	0.889
Exon (mono)	0.864	0.557
Gene (mono)	0.831	0.639

Supplementary Table S3. Counts of selected gene predictions.

	**Predictions**[a]	**N**
L>50	Supported	22,527
	Not supported	6,177
	**Total L>50**	28,704
L<50	Supported	6,393
	Not supported	51,939
	**Total (L>0)**	87,036
L>30	Supported	25,663
	Not supported	13,505
	Filtered (2nd round)[b]	-870
	**Total L>30**	38,298

[a]The prediction is supposed "Supported" when there are evidences supporting the gene model, for instance, a significant alignment with proteins from oomycetes and/or with available transcripts built using cufflinks with our own RNA-Seq data. We used the L>30 dataset for all analyses. RNA-Seq data here refer to the pooled library and not the time-course.

[b] After a second round of filtering to remove potential contaminations, the final number of genes is 38,298.

Supplementary Table S4. Result of the RNAmmer run on *Plasmopara viticola* scaffolds.

Gene	Scaffold	Start	End	Strand	First 2 Blast Hits (% identity)[a]
5S	Scaffold-25542	32	148	+	*Aphanomyces astaci (96%)*, *Albugo laibachii (96%)*
28S	Scaffold-7390	618	4268	-	*Phytophthora megasperma (96%), P. parasitica (96%)*

[a] The first hits are always from *P. viticola* when taking into account partial alignments. The first hits from different species were obtained because the blast e-value is much smaller for these long alignments than for the short perfect matches shared with available *P. viticola* sequences (see also **Supplementary Table S5**).

Supplementary Table S5. Available 28S ribosomal RNA gene sequences were retrieved using as a query the predicted *Plasmopara viticola* 28S rRNA gene.

Description	Max score	Total score	Query cover (scaffold-7390)	E value	Id	Accession
P. vit. isolate AR 160 LSU rib. RNA gene, partial	1749	1749	22%	0	100%	AY035524.2
P. vit. strain TxI 28S rib. RNA gene, partial	1736	1736	21%	0	100%	HM628762.1
P. vit. strain TxIV 28S rib. RNA gene, partial	1687	1687	21%	0	99%	HM628770.1
P. vit. strain MI 28S rib. RNA gene, partial	1674	1674	21%	0	99%	HM628772.1
P. vit. LSU rib. RNA gene, partial	1489	1489	18%	0	99%	AY273978.1
P. vit. strain UASWS SG1 28S rib. RNA gene, complete	1417	1417	17%	0	100%	EF426546.1
P. vit. haplotype 6 28S rib. RNA gene, partial	1297	1297	16%	0	100%	JF897850.1
P. vit. strain 318 28S rib. RNA gene, partial	1291	1291	16%	0	99%	KM279688.1
P. vit. strain 295 28S rib. RNA gene, partial	1291	1291	16%	0	99%	KM279686.1
P. vit. isolate ZH1TZ 28S rib. RNA gene, partial	1291	1291	16%	0	100%	KF160820.1
P. vit. haplotype 5 28S rib. RNA gene, partial	1291	1291	16%	0	99%	JF897849.1
P. vit. strain 307 28S rib. RNA gene, partial	1286	1286	16%	0	100%	KM279687.1
P. vit. isolate XT1CP 28S rib. RNA gene, partial	1286	1286	16%	0	99%	KF160829.1
P. vit. isolate ZS2TZ 28S rib. RNA gene, partial	1284	1284	16%	0	99%	KF160821.1
P. vit. isolate BX2FS 28S rib. RNA gene, partial	1280	1280	16%	0	100%	KF160831.1
P. vit. isolate SH4YQ 28S rib. RNA gene, partial	1275	1275	16%	0	99%	KF160846.1
P. vit. haplotype 2 28S rib. RNA gene, partial	1264	1264	16%	0	99%	JF897853.1
P. vit. isolate AR391 LSU rib. RNA gene, partial	1260	1260	15%	0	100%	AY250173.1
P. vit. haplotype 4 28S rib. RNA gene, partial	1253	1253	16%	0	99%	JF897855.1
P. vit. haplotype 8 28S rib. RNA gene, partial	1253	1253	16%	0	99%	JF897852.1
P. vit. haplotype 3 28S rib. RNA gene, partial	1247	1247	16%	0	99%	JF897854.1
P. vit. haplotype 7 28S rib. RNA gene, partial	1247	1247	16%	0	99%	JF897851.1
P. vit. isolate HV225 LSU rib. RNA gene, partial	1245	1245	15%	0	99%	AY250174.1
P. vit. isolate XH4CP 28S rib. RNA gene, partial	1236	1236	16%	0	99%	KF160848.1
P. vit. haplotype 1 28S rib. RNA gene, partial	1218	1218	16%	0	98%	JF897848.1
P. vit. isolate CY5YQ 28S rib. RNA gene, partial	1214	1214	16%	0	98%	KF160838.1
P. vit. isolate HM5YQ 28S rib. RNA gene, partial	1197	1197	16%	0	98%	KF160847.1
P. vit. isolate CH4YQ 28S rib. RNA gene, partial	1181	1181	16%	0	97%	KF160837.1
P. vit. isolate YB4YQ 28S rib. RNA gene, partial	970	970	16%	0	92%	KF160842.1
P. vit. ITS 1, 5.8S rib. RNA gene, and ITS 2, complete	246	246	3%	1.00E-65	100%	DQ665668.1

Supplementary Table S6. *Plasmopara viticola* tRNA genes predicted by tRNAScan-SE.

Scaffold#	Start	End	Score	Strand	Name
**Alanine**					
3448	10676	10748	47.94	+	Ala_76_tRNA
7207	35	106	58.66	-	Ala_118_tRNA
8544	296	368	66.08	-	Ala_125_tRNA
9115	1	72	42.76	+	Ala_131_tRNA
12323	436	508	32.32	+	Ala_156_tRNA
13109	89	161	39.98	+	Ala_159_tRNA
15606	437	509	27.75	+	Ala_177_tRNA
19475	394	465	58.16	-	Ala_203_tRNA
24294	30	125	56.96	-	Ala_234_tRNA
32332	31	102	50.2	+	Ala_279_tRNA
33956	30	101	66.36	-	Ala_288_tRNA
40072	71	142	53.15	-	Ala_318_tRNA
42292	246	318	60.71	-	Ala_324_tRNA
43939	136	207	63.15	-	Ala_333_tRNA
45463	133	204	63.65	+	Ala_336_tRNA
47648	7	79	57.61	-	Ala_345_tRNA
50705	26	97	63.46	-	Ala_355_tRNA
51237	324	396	49.1	-	Ala_360_tRNA
58083	168	240	53.71	-	Ala_383_tRNA
**Arginine**					
310	18855	18926	30.32	-	Arg_14_tRNA
16194	469	540	60.13	-	Arg_181_tRNA
17303	1	73	67.11	-	Arg_194_tRNA
20362	18	90	71.29	-	Arg_211_tRNA
20973	30	119	62.5	-	Arg_216_tRNA
21176	2	74	62.15	+	Arg_217_tRNA
21505	35	107	50.81	-	Arg_219_tRNA
21587	1	65	50.05	+	Arg_220_tRNA
21962	369	441	68.33	+	Arg_224_tRNA
26077	1	73	70.63	+	Arg_240_tRNA
31008	429	501	62.15	-	Arg_271_tRNA
33311	535	602	50.57	-	Arg_286_tRNA
34855	33	104	52.88	-	Arg_291_tRNA
1821	147	218	58.86	+	Arg_32_tRNA
42326	1	73	68.33	-	Arg_325_tRNA
1821	10368	10439	42.37	-	Arg_34_tRNA
47436	922	989	50.57	-	Arg_344_tRNA
48243	471	543	70.63	+	Arg_346_tRNA
48752	50	122	53.59	-	Arg_347_tRNA
58435	172	245	59.49	+	Arg_384_tRNA
61106	510	579	46.55	+	Arg_391_tRNA
2234	2996	3068	52.17	-	Arg_51_tRNA
2675	2958	3030	71.29	+	Arg_62_tRNA
3146	1075	1147	75.82	+	Arg_72_tRNA
3146	1858	1930	75.82	+	Arg_73_tRNA
4549	1	73	70.63	+	Arg_87_tRNA
**Asparagine**					
2306	20985	21052	31.14	+	Asn_58_tRNA
7207	959	1029	63.94	-	Asn_117_tRNA
11163	1700	1772	60.7	-	Asn_144_tRNA
16759	1	64	51.1	-	Asn_186_tRNA
19481	558	630	78.23	-	Asn_204_tRNA
24235	837	909	70.86	+	Asn_232_tRNA
**Aspartic acid**					
2207	1750	1822	59.25	-	Asp_48_tRNA
19557	367	438	66.24	-	Asp_205_tRNA
**Cysteine**					
2890	421	492	45.29	+	Cys_71_tRNA
5548	3205	3276	67.58	+	Cys_105_tRNA
8439	676	747	68.73	+	Cys_123_tRNA
14797	226	296	50.23	-	Cys_171_tRNA
16297	8	79	69.92	-	Cys_182_tRNA
23457	3	74	73.15	-	Cys_229_tRNA
31320	61	132	49.86	-	Cys_273_tRNA
31602	62	134	66.47	-	Cys_276_tRNA
39540	2	73	68.96	-	Cys_311_tRNA
51690	1282	1353	47.84	-	Cys_362_tRNA
51690	995	1066	57.27	-	Cys_363_tRNA
**Glutamic acid**					
2151	117	189	40.51	-	Glu_44_tRNA
2207	2057	2129	54.27	-	Glu_47_tRNA
2207	860	932	44.61	-	Glu_49_tRNA
2207	129	201	52.24	-	Glu_50_tRNA
5343	884	954	26.35	-	Glu_100_tRNA
5343	129	201	58.3	-	Glu_101_tRNA
5343	2775	2846	52.18	-	Glu_97_tRNA
5343	2414	2486	28.74	-	Glu_98_tRNA
6287	364	436	50.79	-	Glu_113_tRNA
11147	385	456	38.53	-	Glu_140_tRNA
11147	10	82	67.83	-	Glu_141_tRNA
11385	277	349	47.6	+	Glu_145_tRNA
11694	375	447	57.56	-	Glu_151_tRNA
11694	1	73	67.82	-	Glu_152_tRNA
12198	325	396	45.05	+	Glu_154_tRNA
15598	202	270	32.76	-	Glu_176_tRNA
17072	356	428	52.4	-	Glu_192_tRNA
21668	607	679	40.65	+	Glu_221_tRNA
22433	340	411	58.11	+	Glu_226_tRNA
22881	402	470	36.3	-	Glu_228_tRNA
26305	665	737	58.81	-	Glu_242_tRNA
26305	358	430	46.55	-	Glu_243_tRNA
26305	50	122	47.73	-	Glu_244_tRNA
26716	1760	1831	37.95	+	Glu_247_tRNA
26716	2139	2206	53.66	+	Glu_248_tRNA
27798	380	452	50.49	-	Glu_253_tRNA
27798	73	145	50.93	-	Glu_254_tRNA
28119	80	152	61.51	-	Glu_257_tRNA
29909	6	78	50.98	+	Glu_264_tRNA
30523	237	309	53.13	-	Glu_268_tRNA
32925	336	408	50.9	-	Glu_282_tRNA
33239	276	348	68.38	-	Glu_283_tRNA
37879	322	388	37.09	-	Glu_305_tRNA
39604	405	477	44.71	-	Glu_313_tRNA
39604	24	96	58.47	-	Glu_314_tRNA
47415	10	82	41.81	+	Glu_342_tRNA
52979	19932	20005	32.19	+	Glu_368_tRNA
54055	10	82	53.96	+	Glu_373_tRNA
55385	572	644	64.07	-	Glu_377_tRNA
55385	139	210	63.55	-	Glu_378_tRNA
56546	219	290	44.98	+	Glu_380_tRNA
56686	290	358	45.92	-	Glu_381_tRNA
61488	124	195	56.43	-	Glu_392_tRNA
**Glutamine**					
2020	2565	2636	67.98	-	Gln_43_tRNA
2250	2156	2227	27.02	+	Gln_52_tRNA
16924	385	456	59.83	-	Gln_188_tRNA
16924	217	288	44.66	-	Gln_189_tRNA
**Glycine**					
294	19704	19775	54.18	+	Gly_13_tRNA
1103	2930	3001	56.08	+	Gly_24_tRNA
1103	3821	3893	52.17	+	Gly_25_tRNA
1103	4177	4248	64.44	+	Gly_26_tRNA
1821	10693	10764	55.03	-	Gly_33_tRNA
4721	3425	3505	30.5	-	Gly_92_tRNA
5873	23	93	49.44	+	Gly_106_tRNA
6287	1134	1206	54.59	-	Gly_111_tRNA
11158	233	304	42.97	+	Gly_142_tRNA
20437	116	186	56.99	-	Gly_212_tRNA
21909	125	196	62.38	+	Gly_223_tRNA
32410	187	258	56.47	+	Gly_280_tRNA
41282	221	292	60.7	-	Gly_322_tRNA
50086	12	90	20.04	+	Gly_353_tRNA
53847	220	290	46.28	+	Gly_372_tRNA
60152	1	68	42.08	+	Gly_390_tRNA
**Histidine**					
324	22	93	51.84	+	His_15_tRNA
10592	9	80	29.42	+	His_137_tRNA
**Isoleucine**					
357	29390	29463	75.2	+	Ile_16_tRNA
357	31907	31980	69.47	-	Ile_17_tRNA
2485	359	444	34.02	+	Ile_59_tRNA
8679	2506	2578	69.26	+	Ile_128_tRNA
16045	245	317	51.69	+	Ile_179_tRNA
16045	737	809	52.6	+	Ile_180_tRNA
16970	211	283	64.48	+	Ile_190_tRNA
22494	120	192	69.03	+	Ile_227_tRNA
27809	190	262	58.97	-	Ile_255_tRNA

Table S6. continued

Scaffold#	Start	End	Score	Strand	Name
38367	62	134	61.1	+	Ile_309_tRNA
42609	201	273	56.25	-	Ile_326_tRNA
53251	1	64	45.17	-	Ile_369_tRNA
59892	252	323	54.19	-	Ile_389_tRNA
**Leucine**					
60	15630	15710	34.7	-	Leu_4_tRNA
87	523	603	53.24	-	Leu_7_tRNA
433	3756	3836	35.79	+	Leu_18_tRNA
3309	2317	2402	36.03	+	Leu_74_tRNA
4067	3497	3578	42.34	+	Leu_81_tRNA
4380	1646	1727	47.31	-	Leu_86_tRNA
8270	426	510	45.76	-	Leu_122_tRNA
13562	505	586	51.82	+	Leu_161_tRNA
17280	554	635	44.94	+	Leu_193_tRNA
17506	168	247	50.08	-	Leu_195_tRNA
28962	256	341	29.85	+	Leu_260_tRNA
31987	196	277	53.8	-	Leu_277_tRNA
33531	168	249	39.83	-	Leu_287_tRNA
35213	242	327	34.54	-	Leu_293_tRNA
36959	171	250	38.55	-	Leu_297_tRNA
37234	520	601	50.55	-	Leu_299_tRNA
37469	3	88	34.54	-	Leu_303_tRNA
39789	278	357	55.11	-	Leu_315_tRNA
40231	446	531	34.54	-	Leu_319_tRNA
40984	164	243	44.08	-	Leu_321_tRNA
43846	61	135	40.98	-	Leu_332_tRNA
44929	220	301	53.96	-	Leu_335_tRNA
46622	321	398	44.12	-	Leu_340_tRNA
48793	53	132	42.91	+	Leu_348_tRNA
53298	237	316	47.98	-	Leu_371_tRNA
55112	219	298	43.6	+	Leu_376_tRNA
59025	270	346	41.87	+	Leu_387_tRNA
**Lysine**					
582	5674	5746	67.51	+	Lys_20_tRNA
1366	5386	5458	55.4	-	Lys_27_tRNA
3461	4591	4662	64.25	+	Lys_77_tRNA
6287	749	821	50.98	-	Lys_112_tRNA
6802	292	363	73.45	-	Lys_116_tRNA
8563	516	587	59.75	+	Lys_127_tRNA
13918	1708	1779	50.08	-	Lys_163_tRNA
24755	103	175	65.22	+	Lys_236_tRNA
27000	405	476	57.37	-	Lys_249_tRNA
31567	210	282	64.23	+	Lys_274_tRNA
34691	252	324	79.06	+	Lys_290_tRNA
37018	33	105	77.58	+	Lys_298_tRNA
39938	236	308	58.75	-	Lys_317_tRNA
59621	262	323	43.74	+	Lys_388_tRNA
**Methionine**					
2771	2573	2645	49.84	-	Met_67_tRNA
2771	2371	2443	66.96	-	Met_68_tRNA
6393	601	673	74.9	-	Met_114_tRNA
9650	1911	1982	51.86	-	Met_135_tRNA
15598	276	344	34.22	-	Met_175_tRNA
18721	206	278	69.66	-	Met_201_tRNA
18721	4	75	66.52	-	Met_202_tRNA
25289	154	226	59.36	+	Met_237_tRNA
25289	358	430	76.52	+	Met_238_tRNA
25779	138	209	50.7	-	Met_239_tRNA
27505	164	236	63.72	+	Met_251_tRNA
29047	284	355	70.47	+	Met_261_tRNA
29047	837	908	70.47	+	Met_262_tRNA
35621	12	84	72.51	+	Met_294_tRNA
37277	144	215	54.62	-	Met_300_tRNA
38821	238	309	58.54	+	Met_310_tRNA
**Phenylalanine**					
150	6164	6236	56.34	+	Phe_11_tRNA
4601	6710	6782	57.76	+	Phe_88_tRNA
16350	74	146	64.5	-	Phe_183_tRNA
27135	343	445	61.2	+	Phe_250_tRNA
30575	233	310	31.35	-	Phe_269_tRNA
31602	243	315	73.67	-	Phe_275_tRNA
32202	87	173	35.69	-	Phe_278_tRNA
36868	161	232	64.2	-	Phe_296_tRNA
43843	607	676	57.19	+	Phe_331_tRNA
57502	84	156	69.73	+	Phe_382_tRNA
**Proline**					
1949	1419	1490	44.38	+	Pro_36_tRNA
1949	2367	2438	44.38	+	Pro_37_tRNA
1949	3414	3484	49.67	+	Pro_38_tRNA
1949	398	469	41.87	-	Pro_40_tRNA
2579	3405	3476	53.47	-	Pro_61_tRNA
4254	487	557	40.77	+	Pro_83_tRNA
4254	1171	1241	40.77	+	Pro_84_tRNA
4254	1482	1552	24.84	+	Pro_85_tRNA
5873	456	530	63.26	-	Pro_107_tRNA
5879	9589	9667	25.52	+	Pro_108_tRNA
14125	1	65	55.06	-	Pro_168_tRNA
18211	293	364	60.01	+	Pro_197_tRNA
24249	400	471	70.89	-	Pro_233_tRNA
24749	1	64	46.06	+	Pro_235_tRNA
27682	511	582	59.67	+	Pro_252_tRNA
28916	234	305	61.63	-	Pro_259_tRNA
32849	101	172	63.25	-	Pro_281_tRNA
33254	69	140	66.74	-	Pro_285_tRNA
39917	76	147	61.11	-	Pro_316_tRNA
50064	3	71	31.01	-	Pro_352_tRNA
51037	229	300	69.1	-	Pro_358_tRNA

Scaffold#	Start	End	Score	Strand	Name
52413	4	75	66.7	-	Pro_364_tRNA
**Selenocysteine**					
48832	93	164	64.15	+	SeC_349_tRNA
**Serine**					
60	10866	10945	35.08	+	Ser_1_tRNA
60	13215	13295	47.47	+	Ser_2_tRNA
60	14228	14308	43.34	+	Ser_3_tRNA
69	6392	6472	56.73	+	Ser_6_tRNA
103	13192	13272	30.63	-	Ser_9_tRNA
262	10002	10078	25.59	+	Ser_12_tRNA
2890	136	208	33.49	+	Ser_70_tRNA
4721	20836	20916	53.78	-	Ser_90_tRNA
4730	4623	4703	48.02	-	Ser_94_tRNA
5171	365	444	47.18	-	Ser_96_tRNA
5912	1836	1915	46.48	+	Ser_109_tRNA
7581	1054	1134	69.12	+	Ser_119_tRNA
7799	218	303	60.46	+	Ser_120_tRNA
8925	696	776	69.9	+	Ser_129_tRNA
11507	281	360	63.63	-	Ser_147_tRNA
13575	215	295	58.58	+	Ser_162_tRNA
14036	2	82	62.04	+	Ser_164_tRNA
14036	389	469	71.48	+	Ser_165_tRNA
20529	1334	1407	39.02	-	Ser_213_tRNA
20529	776	849	39.02	-	Ser_214_tRNA
20529	218	291	39.02	-	Ser_215_tRNA
23843	399	479	75.57	-	Ser_230_tRNA
26230	166	246	54.47	-	Ser_241_tRNA
28062	72	152	67.66	-	Ser_256_tRNA
29386	388	460	33.22	-	Ser_263_tRNA
33254	413	484	63.23	-	Ser_284_tRNA
38002	56	135	64.53	-	Ser_306_tRNA
41364	137	217	55.09	-	Ser_323_tRNA
43076	110	190	76.42	+	Ser_327_tRNA
49083	58	129	62.87	-	Ser_350_tRNA
49196	1	78	54.56	-	Ser_351_tRNA
50852	158	229	74.14	-	Ser_357_tRNA
**Stop**					
20123	267	339	58.91	-	Sup_209_tRNA
54787	90	176	37.34	-	Sup_375_tRNA
**Threonine**					
5456	4023	4094	67.03	-	Thr_103_tRNA
5456	3777	3847	59.51	-	Thr_104_tRNA
6802	608	679	47.42	-	Thr_115_tRNA
9072	406	477	56.38	-	Thr_130_tRNA
9548	628	699	76.55	-	Thr_132_tRNA
9548	314	385	69.39	-	Thr_133_tRNA
9548	1	67	56.27	-	Thr_134_tRNA
10734	199	270	61.38	-	Thr_138_tRNA
11674	1	61	36.54	-	Thr_150_tRNA
13411	337	408	61.33	-	Thr_160_tRNA
14827	1	68	53.61	+	Thr_172_tRNA
15564	486	557	53.44	+	Thr_174_tRNA
15688	448	514	64.24	+	Thr_178_tRNA
16794	39	110	74.6	-	Thr_187_tRNA
22125	1	65	46.3	-	Thr_225_tRNA
30251	508	580	68.15	-	Thr_266_tRNA
30251	213	284	69.3	-	Thr_267_tRNA
36122	68	138	57.19	-	Thr_295_tRNA
37281	247	318	68.28	+	Thr_301_tRNA
37322	162	233	65.64	-	Thr_302_tRNA
37734	129	200	72.76	-	Thr_304_tRNA
43298	129	200	69.83	+	Thr_328_tRNA
43663	393	464	72.49	-	Thr_329_tRNA
43663	81	152	65.02	-	Thr_330_tRNA
45639	677	741	46.3	+	Thr_337_tRNA
45786	236	307	70.4	+	Thr_338_tRNA
47185	1	62	50.86	-	Thr_341_tRNA
53268	1	67	56.24	-	Thr_370_tRNA
**Tryptophan**					
11158	589	659	48.75	+	Trp_143_tRNA
11670	567	638	59.26	-	Trp_148_tRNA
16637	163	234	64.55	+	Trp_185_tRNA
17047	399	469	54.98	-	Trp_191_tRNA
30046	323	394	74.55	-	Trp_265_tRNA
**Tyrosine**					
982	3893	3995	74.35	+	Tyr_22_tRNA
19825	209	311	48.27	-	Tyr_206_tRNA
21869	77	157	58.67	-	Tyr_222_tRNA
24097	50	152	42.39	-	Tyr_231_tRNA
28817	273	332	38.8	+	Tyr_258_tRNA
30955	6	108	73.08	+	Tyr_270_tRNA
31189	77	136	31.22	+	Tyr_272_tRNA
34553	77	136	37.3	+	Tyr_289_tRNA
**Valine**					
103	13599	13671	52.42	-	Val_8_tRNA
103	12737	12809	58.74	-	Val_10_tRNA
1454	11010	11082	63.72	+	Val_28_tRNA
1593	1155	1227	42.98	+	Val_29_tRNA
1593	1497	1568	56.06	+	Val_30_tRNA
1593	2089	2160	59.08	+	Val_31_tRNA
3466	134	204	51.51	+	Val_78_tRNA
10787	4381	4450	30.56	-	Val_139_tRNA
11434	445	516	34.68	+	Val_146_tRNA
12269	836	906	51.51	-	Val_155_tRNA
14065	147	219	65.8	-	Val_167_tRNA
16361	215	287	54.44	-	Val_184_tRNA
56115	515	584	30.56	+	Val_379_tRNA

Supplementary Table S7. List of species names and abbreviations for the organisms composing the "oomycetes dataset" used in the comparative analyses.

Full Name	Abbre	Taxonomy ID	Full Taxonomy
Plasmopara viticola	PVIT	143451	Stramenopiles; Oomycetes; Peronosporales; Peronosporaceae;
Plasmopara halstedii	PHAL	4781	Stramenopiles; Oomycetes; Peronosporales; Peronosporaceae;
Hyaloperonospora arabidopsidis	HARA	27295	Stramenopiles; Oomycetes; Peronosporales; Peronosporaceae;
Pythium aphanidermatum	PAG1	65070	Stramenopiles; Oomycetes; Pythiales; Pythiaceae
Pythium arrhenomanes	PAR	82932	Stramenopiles; Oomycetes; Pythiales; Pythiaceae
Pythium irregulare	PIR	36331	Stramenopiles; Oomycetes; Pythiales; Pythiaceae
Pythium iwayamai	PIW	115417	Stramenopiles; Oomycetes; Pythiales; Pythiaceae
Pythium ultimum	PUG3	65071	Stramenopiles; Oomycetes; Pythiales; Pythiaceae
Pythium vexans	PVE	42099	Stramenopiles; Oomycetes; Pythiales; Pythiaceae
Phytophthora sojae	PHYS	67593	Stramenopiles; Oomycetes; Peronosporales
Phytophthora ramorum	PHYR	164328	Stramenopiles; Oomycetes; Peronosporales
Phytophthora capsici	PHYC	4784	Stramenopiles; Oomycetes; Peronosporales
Phytophthora infestans	PHYIN	4787	Stramenopiles; Oomycetes; Peronosporales
Phytophthora cinnamomi	PHYCI	4785	Stramenopiles; Oomycetes; Peronosporales
Albugo laibachii	ALA	653948	Stramenopiles; Oomycetes; Albuginales; Albuginaceae

Supplementary Table S8. The 10 most represented KEGG categories in the core genome of the oomycetes.

KEGG category	Fraction[a]	N
01110 Biosynthesis of secondary metabolites	0.063	43
03010 Ribosome	0.044	30
01120 Microbial metabolism in diverse environments	0.032	22
03040 Spliceosome	0.026	18
01200 Carbon metabolism	0.025	17
00230 Purine metabolism	0.022	15
01230 Biosynthesis of amino acids	0.022	15
00190 Oxidative phosphorylation	0.021	14
03013 RNA transport	0.019	13
00240 Pyrimidine metabolism	0.018	12

[a]One *P. viticola* protein per cluster was taken, the KEGG categories recorded and then counted for all of them. The fraction is calculated over the proteins with KEGG annotations (N=680 after excluding those mapped to very broad categories, e.g. Metabolism).

Supplementary Table S9. BUSCO analysis

SPECIES	Complete	Single copy	Duplicated	Fragmented	Complete + Fragmented	Missing	Number of eukaryote proteins
Phytophthora infestans	**93.00%**	**86.10%**	**6.90%**	**1.30%**	**94.30%**	**5.70%**	**303[a]**
Plasmopara halstedii	93.40%	90.80%	2.60%	3.00%	96.40%	3.60%	**303[a]**
Plasmopara viticola **(Yin *et al.* 2017)**	84%	38%**	46%	6%	90%	9.50%	429[b]
Plasmopara viticola **(this work)**	73.00%	70.00%	3.00%	14.20%	**87.20%**	12.80%	**303[a]**

*** estimated, as Complete=Single Copy + Duplicated*
[a] BUSCO version 3
[b] BUSCO version 1

Supplementary Table S10. Summary of the output of the search of occurrences of the two regular expressions R[A-Z]LR and [DE][DE][RK], for the RxLR and the often associated EER.

Species[a]	R[b]	R+E[c]	R+E[c] (150)	S+R[d]	P Enrichment S in R	S+R (60)	S+R+E[e]	S+R+E[e] (60,150)
PVIT	493	73	33	31	1.37E-03	24	15	12
ALA	134	21	9	17	8.51E-05	8	6	1
HARA	186	56	23	38	2.41E-10	23	19	9
PHYCA	356	142	40	135	0.00E+00	70	66	54
PHYCI	441	164	54	149	0.00E+00	94	77	60
PHYIN	430	206	37	266	0.00E+00	200	158	140
PHYRA	271	117	31	140	0.00E+00	58	67	42
PHYSO	508	195	43	246	0.00E+00	137	108	94
PAG1	131	37	9	45	0.00E+00	21	10	1
PIR	174	44	15	50	1.78E-15	24	8	0
PIW	197	49	14	54	0.00E+00	32	11	7
PUG3	207	55	14	30	8.77E-07	16	8	3
PVE	115	23	10	32	2.42E-10	7	7	0
PAR	146	33	15	39	1.32E-13	16	6	2
PHAL	148	44	16	29	1.01E-07	14	16	7

[a]PVIT: *Plasmopara viticola,* ALA: *Albugo laibachii*, HARA: *Hyaloperonospora arabidopsidis,* PHYCA: *Phytophthora capsici*, PHYCI: *Phytophthora cinnamomi*, PHYIN: *Phytophthora infestans*, PHYRA: *Phytophthora ramorum,* PHYSO: *Phytophthora sojae*, PAG1: *Pythium aphanidermatum,* PIR: *Pythium irregulare,* PIW: *Pythium iwayamai,* PUG3*: Pythium ultimum,* PVE: *Pythium vexans,* PAR: *Pythium arrhenomanes,* PHAL: *Plasmopara halstedii*.
[b]R indicates the number of RxLR occurrences
[c]E the number of EER occurrences (considered only when associated to the presence of an RxLR, indicated by R+E).
[d]S indicates the presence of a signal peptide for secretion as predicted by SignalP. Since the RxLR is often positionally constrained.
[e]We also counted how many times the occurrences are within certain ranges (indicated in parenthesis). For instance, column S+R+E (60,150) corresponds to the counts of proteins having the signal (S), the RxLR within 60 aa from the predicted cleavage site and and EER within 150 aa from the end of the RxLR. This is the most stringent definition and it is often adopted in *Phytophthora* genomic studies. All counts refer to RxLR occurrences with a p-value<0.05 calculated using shuffling of the protein sequences.

Supplementary Table S11. Focus on sequences with a single occurrence of the RxLR motif or one of its variants.

Species	RxLR	RxLK	KxLR	KxLK	QxLR	QxLK	TOT	ALT/TOT[a]
A. laibachii	1	0	0	0	1	0	**2**	**0.50**
P. aphanidermatum	3	0	2	1	0	0	**6**	**0.50**
P. arrhenomanes	1	0	2	0	3	0	**6**	**0.83**
P. irregulare	3	1	0	1	4	0	**9**	**0.67**
P. iwayamai	9	0	0	1	2	0	**12**	**0.25**
P. ultimum	4	0	0	1	0	0	**5**	**0.20**
P. vexans	0	0	1	0	1	0	**2**	**1.00**
P. capsicii	71	8	0	4	2	0	**85**	**0.16**
P. cinammomi	80	14	5	2	3	0	**104**	**0.23**
P. infestans	189	14	4	3	8	0	**218**	**0.13**
P. ramorum	47	15	0	3	4	0	**69**	**0.32**
P. sojae	121	5	8	8	0	0	**142**	**0.15**
P. halstedii	2	5	1	2	0	0	**10**	**0.80**
P. viticola FEM	16	0	1	0	4	1	**22**	**0.27**
H. arabidopsidis	6	0	0	2	1	3	**12**	**0.50**
TOT	**553**	**62**	**24**	**28**	**33**	**4**	**704**	**0.21**

[a] Fraction of non-canonical sites with respect to the total in a certain organism.

Supplementary Table S12. Counts of oomycetes sequences containing single or multiple RxLR occurrences within the RxLR-like set in addition to the signal peptide and the EER occurrence within 150 residues.

	RxLR	RxLK	KxLR	KxLK	QxLR	QxLK	TOT	ALT/TOT[a]
RxLR-like motif has unique occurrence	553	62	24	28	33	4	**704**	**0.21**
Most N-terminal when there are more variants	315	35	31	26	16	13	**436**	**0.28**

[a] Fraction of non-canonical sites with respect to the total in a certain organism.

Supplementary Table S13. Output of the SVM classification: number of CRN proteins per genome and number of CRN proteins also predicted to be secreted.

Species[a]	# CRN[b]	# CRN+S[c]	P[d]
PVIT	40	5	3.06E-03
ALA	2	0	-
HARA	23	8	1.01E-05
PHYCA	64	14	8.01E-05
PHYCI	13	2	8.15E-02
PHYIN	260	48	7.62E-05
PHYRA	21	5	2.97E-02
PHYSO	68	10	4.61E-02
PAG1	5	1	6.50E-02
PAR	4	0	-
PIR	2	1	7.18E-03
PIW	2	1	6.36E-03
PUG3	4	0	-
PVE	3	0	-

[a]PVIT: *Plasmopara viticola,* ALA: *Albugo laibachii*, HARA: *Hyaloperonospora arabidopsidis,* PHYCA: *Phytophthora capsici*, PHYCI: *Phytophthora cinnamomi*, PHYIN: *Phytophthora infestans*, PHYRA: *Phytophthora ramorum,* PHYSO: *Phytophthora sojae*, PAG1: *Pythium aphanidermatum,* PAR: *Pythium arrhenomanes,* PIR: *Pythium irregulare,* PIW: *Pythium iwayamai,* PUG3*: Pythium ultimum,* PVE: *Pythium vexans*.
[b]The CRN classification is performed without using the information about the presence of the signal peptide.
[c]S, proteins with signal-peptide predicted by SignalP.
[d]The **P** column is the probability of random sampling as many secreted sequences. The probability is calculated using the binomial cumulative distribution function with the number of trials equal to the number of proteins predicted to be CRN, number of successes the number of CRN proteins also having a signal peptide, and the probability of success in the null hypothesis given by the number of proteins predicted as being secreted over the total number of genes.

Supplementary Table S14. CRN proteins identified by scanning protein sequences with regular expression.

Species[a]	# L[b]	# L+V[c]	# S+L[d]	# S+L+V	P[e]
PVIT	45	7	5	1	5.56E-03
ALA	6	2	0	0	na
HARA	20	0	11	0	6.93E-10
PHYIN	155	76	41	20	1.27E-08
PHYCA	71	24	15	6	7.78E-05
PHYCI	11	4	2	1	5.30E-02
PHYRA	15	6	2	0	2.54E-01
PHYSO	84	43	13	7	2.12E-02
PAG1	8	0	1	0	1.53E-01
PAR	8	0	1	0	1.10E-01
PIR	2	0	0	0	na
PIW	5	0	0	0	na
PUG3	6	1	0	0	na
PVE	3	0	0	0	na

[a]PVIT: *Plasmopara viticola,* ALA: *Albugo laibachii*, HARA: *Hyaloperonospora arabidopsidis,* PHYIN: *Phytophthora infestans*, PHYCA: *Phytophthora capsici*, PHYCI: *Phytophthora cinnamomi*, PHYRA: *Phytophthora ramorum,* PHYSO: *Phytophthora sojae*, PAG1: *Pythium aphanidermatum,* PAR: *Pythium arrhenomanes,* PIR: *Pythium irregulare,* PIW: *Pythium iwayamai,* PUG3: *Pythium ultimum,* PVE: *Pythium vexans*.
[b]L=LFLAK motif
[c]V=VVP motif,
[d]S, proteins with signal-peptide predicted by SignalP.
[e]The P column contains the p-value of the enrichment in secreted proteins in the group of proteins with a LFLAK motif. All counts refer to LFLAK motifs with occurrence p-value≤0.05.

Supplementary Table S15. YxSLK were assigned by scanning protein sequences using the regular expression Y[A-Z][ST][LV][KR].

Species[a]	**# YxSLK occurrences[b]**	**# YxSLK Secreted [c]**	**Enrichment in Secreted[d]**
PVIT	308 (194)	25 (13)	**7.48E-05**
ALA	153 (86)	9 (4)	2.06E-01
HARA	164 (75)	14 (9)	1.45E-01
PHYCA	291 (153)	45 (30)	**2.65E-06**
PHYCI	315 (168)	36 (22)	**1.42E-02**
PHYSO	450 (261)	61 (33)	**1.02E-03**
PHYIN	297 (146)	43 (21)	**1.87E-02**
PHYRA	263 (131)	46 (35)	**2.24E-03**
PIR	216 (86)	39 (26)	**2.32E-06**
PUG3	180 (90)	18 (10)	**7.16E-03**
PIW	217 (89)	27 (18)	**8.14E-03**
PAG1	182 (75)	31 (13)	**1.34E-04**
PVE	180 (76)	25 (14)	**5.01E-03**
PAR	231 (108)	26 (15)	**9.73E-03**

[a]PVIT: *Plasmopara viticola,* ALA: *Albugo laibachii*, HARA: *Hyaloperonospora arabidopsidis,* PHYCA: *Phytophthora capsici*, PHYCI: *Phytophthora cinnamomi*, PHYSO: *Phytophthora sojae*, PHYIN: *Phytophthora infestans*, PHYRA: *Phytophthora ramorum,* PIR: *Pythium irregulare,* PUG3: *Pythium ultimum,* PIW: *Pythium iwayamai,* PAG1: *Pythium aphanidermatum,* PVE: *Pythium vexans,* PAR: *Pythium arrhenomanes.*

[b]The numbers in parenthesis indicate the number of YxSLK occurrences within 100 residues from the translation start site of the protein.

[c]The term Secreted corresponds to proteins for which SignalP [39] predicted a signal peptide.

[d]The p-value indicating if and in what degree the YxSLK group from an organism is enriched in secreted proteins. *P. viticola* has the largest number of members of this class among the biotrophs, comparable to the *Pythium* species, but much lower than *Phytophthora* species.

Supplementary Table S16. Pfam models used to define families of apoplastic effectors. For all models, for inclusion, we used a threshold of 1E-06 on the "e-value seq" field in the HMMER output.

Class	Pfam name	Pfam model ID
Cystatins	Cystatin	PF00031
	Elicitin	PF00964
Serine protease inhibitor	Kazal_1	PF00050
	Kazal_2	PF07648
Necrosis inducing proteins	NPP1	PF05630
CBEL	PAN_1	PF00024
	PAN_4	PF14295
	PcF	PF09461
	Transglut_C	PF00927
Transglutaminase	Transglut_core	PF01841
	Transglut_N	PF00868
Glucanase inhibitor	Trypsin	PF00089

Supplementary Table S17: Number of read pairs (in millions) obtained in the different libraries and mapping on *P. viticola* and *V. vinifera* genomes.

	Non-infected (hpi)						Infected (hpi)					Pooled libraries		
	0	24	48	72	96	168	24	48	72	96	168	Non-infected (C)	Infected (I)	Sporangia (S)
Total in RNAseq library	63.9	81.4	74.3	91.6	86.8	84.8	80.3	97.6	81.1	97.9	86.6	30.5	20.5	23.5
Mapping on *Plasmopara viticola* genome	-	-	-	-	-	-	-	1.2	2.0	6.8	8.9	-	0.7	11.6
Mapping on *Vitis vinifera* genome	48.4	62.7	30.6	68.5	65.4	64.3	62.3	63.1	59.7	67.8	56.9	14.6	12.9	0.1

Supplementary Table S18. See attached Excel file "*P. viticola* differentially expressed genes during infection".

Supplementary Table S19. *Plasmopara viticola* cellular processes enriched genes detected as having gene expression significantly different from 0 at 24 and 48 hpi.

GO term[a]	Term	#q	#ref	p	FDR
**At 24 hpi**					
0009058	bios. p.	71	2549	1.9E-08	2.9E-06
0016051	carbohydrate bios. p.	6	102	1.7E-03	4.2E-02
0019752	carboxylic acid metabolic p.	17	470	1.3E-04	5.6E-03
0006519	cell. amino acid and derivative metabolic p.	12	356	2.0E-03	4.7E-02
0044249	cell. bios. p.	70	2445	8.6E-09	2.4E-06
0034637	cell. carbohydrate bios. p.	6	91	9.7E-04	2.5E-02
0006073	cell. glucan metabolic p.	5	39	1.2E-04	5.6E-03
0042180	cell. ketone metabolic p.	17	481	1.7E-04	6.5E-03
0034645	cell. macromolecule bios. p.	55	1864	7.3E-08	8.6E-06
0033692	cell. polysaccharide bios. p.	5	49	3.5E-04	1.2E-02
0044264	cell. polysaccharide metabolic p.	5	52	4.6E-04	1.4E-02
0044267	cell. protein metabolic p.	63	2163	1.9E-08	2.9E-06
0006631	fatty acid metabolic p.	5	62	1.0E-03	2.6E-02
0010467	gene expression	50	1327	1.5E-10	6.2E-08
0009250	glucan bios. p.	5	39	1.2E-04	5.6E-03
0044042	glucan metabolic p.	6	59	9.2E-05	5.4E-03
0009059	macromolecule bios. p.	57	1883	2.1E-08	2.9E-06
0006082	organic acid metabolic p.	17	472	1.4E-04	5.6E-03
0043436	oxoacid metabolic p.	17	470	1.3E-04	5.6E-03
0000271	polysaccharide bios. p.	5	58	7.6E-04	2.0E-02
0010608	Posttranscr. Reg. of gene expression	6	79	4.6E-04	1.4E-02
0019538	protein metabolic p.	69	2709	5.3E-07	4.9E-05
0006164	purine nucleotide bios. p.	6	81	5.2E-04	1.5E-02
0009152	purine ribonucleotide bios. p.	6	78	4.3E-04	1.4E-02
0032268	Reg. of cell. protein metabolic p.	8	134	2.9E-04	1.1E-02
0051246	Reg. of protein metabolic p.	8	149	5.9E-04	1.7E-02
0006417	Reg. of translation	6	66	1.7E-04	6.5E-03
0046686	resp. to cadmium ion	6	25	5.2E-07	4.9E-05
0010035	resp. to inorganic substance	7	40	5.9E-07	4.9E-05
0010038	resp. to metal ion	6	30	1.7E-06	1.1E-04
0050896	resp. to stimulus	24	758	5.8E-05	3.7E-03
0006950	resp. to stress	19	552	1.1E-04	5.6E-03
0009260	ribonucleotide bios. p.	6	86	7.2E-04	2.0E-02
0006412	translation	45	418	3.7E-26	3.0E-23
0006414	translational elongation	9	82	9.4E-07	7.0E-05
**At 48 hpi**					
0065007	biological reg.	329	1592	3.7E-12	9.4E-11
0009058	biosynthetic proc.	527	2549	2.0E-17	1.5E-15
0005975	carbohydrate metabolic proc.	114	552	1.6E-05	1.6E-04
0009056	catabolic proc.	278	1225	2.9E-15	1.4E-13
0030154	cell differentiation	51	172	1.0E-07	1.3E-06
0006519	cell. amino acid and derivative metabolic proc.	113	356	2.8E-17	1.8E-15
0044249	cell. biosynthetic proc.	513	2445	3.2E-18	3.0E-16
0016043	cell. component organization	211	904	2.1E-13	7.8E-12
0019725	cell. homeostasis	35	100	1.2E-07	1.4E-06
0034645	cell. macromolecule biosynthetic proc.	358	1864	3.0E-09	4.2E-08
0044267	cell. protein metabolic proc.	472	2163	4.3E-20	5.4E-18
0051234	establishment of localization	258	1461	1.0E-04	9.3E-04
0045184	establishment of protein localization	102	362	3.2E-12	9.2E-11
0010467	gene expression	395	1327	6.9E-45	1.3E-42
0006091	generation of precursor metabolites and energy	38	107	2.2E-08	3.0E-07
0042592	homeostatic proc.	41	134	6.8E-07	7.4E-06
0051179	localization	270	1556	2.2E-04	1.9E-03
0009059	macromolecule biosynthetic proc.	362	1883	2.3E-09	3.3E-08
0033036	macromolecule localization	123	476	1.5E-11	3.4E-10
0006996	organelle organization	135	526	2.9E-12	9.0E-11
0008104	protein localization	111	407	3.8E-12	9.4E-11
0019538	protein metabolic proc.	553	2709	4.7E-17	2.5E-15
0015031	protein transport	102	361	2.7E-12	9.0E-11
0050789	reg. of biological proc.	297	1461	1.7E-10	3.1E-09
0065008	reg. of biological quality	82	279	3.6E-11	7.5E-10
0032535	reg. of cell. component size	18	59	8.6E-04	7.1E-03
0050794	reg. of cell. proc.	278	1384	1.8E-09	2.7E-08
0010468	reg. of gene expression	123	501	4.6E-10	7.5E-09
0040029	reg. of gene expression, epigenetic	8	17	1.1E-03	8.6E-03
0060255	reg. of macromolecule metabolic proc.	143	595	1.1E-10	2.0E-09
0019222	reg. of metabolic proc.	160	671	1.9E-11	4.2E-10
0009628	resp. to abiotic stimulus	53	176	3.4E-08	4.3E-07
0009719	resp. to endogenous stimulus	23	74	1.3E-04	1.2E-03
0009605	resp. to external stimulus	29	93	1.8E-05	1.7E-04
0050896	resp. to stimulus	171	758	4.0E-10	6.8E-09
0006950	resp. to stress	119	552	1.4E-06	1.4E-05
0006350	transcription	109	514	7.5E-06	7.6E-05
0006412	translation	195	418	1.1E-55	4.2E-53
0006810	transport	257	1456	1.1E-04	9.6E-04

[a]GO annotation is available for 173 of these genes and a total of 14,816 genes in the proteome. The latter was used as the reference annotation in the analysis. Abbreviations: cell.=cellular; p.=process; resp.=response; bios.=biosynthesis; Posttranscr.=posttranscriptional.

Supplementary Table S20. Gene Ontology (GO) annotation of RxLR-like genes expressed at 24 hpi.

GO term	Ontology	Description	#q	#ref	p	FDR
0030554	F	adenyl nucleotide binding	23	1909	5.2E-05	5.5E-04
0032559	F	adenyl ribonucleotide binding	21	1809	1.5E-04	1.2E-03
0005524	F	ATP binding	21	1787	1.3E-04	1.1E-03
0016798	F	hydrolase activity, acting on glycosyl bonds	6	215	4.0E-04	2.8E-03
0004553	F	hydrolase activity, hydrolyzing O-glycosyl compounds	6	208	3.4E-04	2.5E-03
0016301	F	kinase activity	19	831	3.1E-08	1.1E-06
0001882	F	nucleoside binding	23	1916	5.5E-05	5.5E-04
0000166	F	nucleotide binding	28	2422	2.4E-05	3.8E-04
0016773	F	phosphotransferase activity, alcohol group as acceptor	19	678	1.3E-09	7.2E-08
0004672	F	protein kinase activity	17	530	1.1E-09	7.2E-08
0004674	F	protein serine/threonine kinase activity	13	394	5.4E-08	1.5E-06
0001883	F	purine nucleoside binding	23	1912	5.3E-05	5.5E-04
0017076	F	purine nucleotide binding	27	2169	9.6E-06	2.1E-04
0032555	F	purine ribonucleotide binding	25	2069	2.8E-05	3.8E-04
0032553	F	ribonucleotide binding	25	2069	2.8E-05	3.8E-04
0016740	F	transferase activity	24	2530	9.3E-04	5.7E-03
0016746	F	transferase activity, transferring acyl groups	7	312	5.1E-04	3.3E-03
0016772	F	transferase activity, transferring phosphorus-containing groups	20	1630	1.1E-04	9.6E-04
0043412	P	macromolecule modification	21	1578	2.6E-05	6.7E-04
0006796	P	phosphate metabolic process	22	1402	1.6E-06	7.3E-05
0006793	P	phosphorus metabolic process	22	1402	1.6E-06	7.3E-05
0016310	P	phosphorylation	21	1125	1.9E-07	1.7E-05
0043687	P	post-translational protein modification	20	1295	5.1E-06	1.9E-04
0006468	P	protein amino acid phosphorylation	19	748	6.1E-09	1.1E-06
0006464	P	protein modification process	20	1434	2.0E-05	6.1E-04

Supplementary Table S21*:* Gene Ontology (GO) categories enrichment of differentially expressed genes (DEGs) in *Plasmopara viticola*

GO term	GO	Description	# in	# in	p-value	FDR
0016043	P	cellular component organization	95	886	5.7E-10	1.7E-07
0048869		cellular developmental process	40	269	6.8E-09	1.0E-06
0009653		anatomical structure morphogenesis	33	209	2.9E-08	2.9E-06
0032502		developmental process	59	510	4.6E-08	3.5E-06
0032501		multicellular organismal process	49	454	3.7E-06	2.1E-04
0009056		catabolic process	101	1171	4.6E-06	2.1E-04
0048856		anatomical structure development	45	408	4.9E-06	2.1E-04
0003774	F	motor activity	39	241	9.2E-10	1.8E-07
0016818		hydrolase activity, acting on acid anhydrides, in P-containing [illegible]	91	950	1.9E-07	9.4E-06
0016462		pyrophosphatase activity	91	949	1.8E-07	9.4E-06
0017111		nucleoside-triphosphatase activity	87	932	9.1E-07	3.6E-05
0005730	C	nucleolus	38	161	1.0E-14	2.2E-12
0043232		intracellular non-membrane-bounded organelle	123	1166	1.0E-11	7.3E-10
0043228		non-membrane-bounded organelle	123	1166	1.0E-11	7.3E-10
0031974		membrane-enclosed lumen	53	388	7.8E-10	4.2E-08
0043233		organelle lumen	52	382	1.2E-09	4.4E-08
0070013		intracellular organelle lumen	52	382	1.2E-09	4.4E-08
0031981		nuclear lumen	47	340	4.3E-09	1.3E-07
0015630		microtubule cytoskeleton	50	415	1.2E-07	3.1E-06
0044422		organelle part	149	1748	1.3E-07	3.1E-06
0044430		cytoskeletal part	57	512	2.6E-07	5.6E-06
0005856		cytoskeleton	63	597	4.4E-07	8.6E-06
0044446		intracellular organelle part	142	1730	1.6E-06	2.8E-05
0005929		cilium	15	67	1.9E-06	3.1E-05
0042995		cell projection	21	127	3.8E-06	5.8E-05
0030529		ribonucleoprotein complex	43	397	1.2E-05	1.7E-04
0032991		macromolecular complex	142	1868	5.2E-05	7.0E-04

Supplementary Table S22. FPKM values of *Plasmopara viticola* secreted genes differentially expressed during infection.

P. viticola gene ID	24 hpi	48 hpi	72 hpi	96 hpi	168 hpi	Effector family	Description
PVITv1021061	101,819	126,359	32,185	33,569	0.00	RxLR-R	
PVITv1001084	618.71	337.70	954.19	200.19	232.07	-	
PVITv1012062	536.78	262.06	60.64	155.48	51.70	-	
PVITv1035002	236.86	74.22	633.26	520.49	467.85	-	
PVITv1005727	130.02	223.86	91.14	133.14	48.51	-	elicitin-like INF6
PVITv1006535	47.92	8.65	16.25	3.10	4.68	-	
PVITv1030766	46.47	54.22	199.26	234.34	301.00	-	glycoside hydrolase, putative
PVITv1005799	41.00	88.18	601.70	261.49	285.12	-	glycoside hydrolase, putative
PVITv1003492	39.74	153.92	91.42	114.16	35.26	RxLR-R	
PVITv1032354	33.85	84.13	126.64	201.68	87.91	-	
PVITv1020539	32.32	46.15	201.11	165.66	229.49	-	cysteine protease family C01A, putative
PVITv1013162	23.69	63.32	219.20	212.53	214.63	-	
PVITv1006070	21.86	26.69	12.44	25.82	8.95	YXSLK	conserved hypothetical protein
PVITv1022382	21.02	70.73	29.57	54.19	26.26	-	conserved hypothetical protein
PVITv1016618	18.99	26.37	175.70	102.06	144.62	-	callose synthase, putative
PVITv1019732	18.85	1.45	1.51	116.40	101.73	-	glycoside hydrolase, putative
PVITv1020466	17.44	14.64	87.56	54.44	118.82	-	cysteine protease family C01A, putative
PVITv1030481	15.23	0.00	97.44	53.96	82.34	-	conserved hypothetical protein
PVITv1035979	14.34	0.00	31.62	25.71	29.61	-	protease inhibitor Epi7
PVITv1013878	14.24	44.56	313.59	181.00	366.07	-	conserved hypothetical protein
PVITv1004487	11.86	0.00	81.84	30.47	91.65	-	cutinase, putative
PVITv1022330	10.05	48.95	470.29	427.00	567.39	-	glucan 1,3-beta-glucosidase, putative
PVITv1020375	8.64	12.24	225.85	113.22	166.94	-	
PVITv1036266	7.55	7.16	6.42	12.72	4.35	RxLR-H	
**PVITv1008311**	**4.60**	**0.00**	**9.98**	**9.83**	**17.82**	**RxLR-H**	
PVITv1002158	3.69	35.09	15.26	27.33	13.20	-	60S ribosomal export protein NMD3, putative
PVITv1011616	2.50	0.61	1.74	2.75	12.66	-	phospholipid-transporting ATPase, putative
PVITv1018231	1.75	5.10	73.78	74.13	87.10	-	mucin-like protein
PVITv1033458	0.00	1.71	2.50	6.70	2.40	-	
PVITv1017335	0.00	0.00	3.37	1.27	12.90	-	
PVITv1015244	0.00	0.00	7.07	6.35	8.42	-	
PVITv1025503	0.00	0.00	7.83	5.72	8.43	-	mucin-like protein
PVITv1020385	0.00	5.10	3.51	10.72	4.09	-	
PVITv1000022	0.00	0.00	7.23	11.12	7.18	-	glycoside hydrolase, putative
PVITv1021406	0.00	0.00	17.34	2.12	8.35	-	conserved hypothetical protein
PVITv1026328	0.00	0.00	18.10	2.49	10.04	-	sporangia induced conserved hypothetical protein
PVITv1005240	0.00	1.58	22.18	0.87	7.06	-	conserved hypothetical protein
PVITv1015025	0.00	0.00	10.93	9.60	11.81	-	uridine kinase
PVITv1033929	0.00	0.00	10.52	11.66	10.62	-	conserved hypothetical protein
PVITv1016178	0.00	0.00	19.35	14.39	5.56	-	
PVITv1024386	0.00	0.00	15.03	11.85	12.77	-	conserved hypothetical protein
PVITv1015555	0.00	19.19	7.39	10.74	3.43	-	carbohydrate esterase, putative
PVITv1026360	0.00	1.49	11.18	5.01	23.48	-	endoglucanase, putative
PVITv1003147	0.00	3.91	16.45	19.31	8.56	-	
PVITv1002840	0.00	7.27	29.48	4.63	11.48	-	similar to sexually induced protein 3
PVITv1002288	0.00	0.00	0.00	29.95	32.95	-	glycoside hydrolase, putative
PVITv1007968	0.00	0.00	31.52	17.53	24.68	-	
PVITv1024576	0.00	0.00	37.19	7.00	42.04	-	conserved hypothetical protein
PVITv1019683	0.00	0.00	43.85	11.82	31.66	-	conserved hypothetical protein
PVITv1016506	0.00	0.00	34.36	23.75	30.23	-	hypothetical protein
PVITv1037885	0.00	45.59	13.42	23.53	5.96	-	glucanase inhibitor protein, putative
PVITv1003338	0.00	14.82	16.33	46.63	17.81	-	
PVITv1018548	0.00	0.00	25.67	38.69	37.17	-	glycoside hydrolase, putative
PVITv1025849	0.00	7.56	26.81	19.77	52.61	-	
PVITv1020618	0.00	13.65	9.57	31.23	57.79	-	glucan 1,3-beta-glucosidase, putative
PVITv1037902	0.00	0.00	61.82	12.63	40.07	-	conserved hypothetical protein
PVITv1023922	0.00	0.00	44.07	27.97	43.77	-	methylmalonate semialdehyde dehydrogenase
PVITv1029873	0.00	3.48	3.09	54.12	56.27	-	conserved hypothetical protein
PVITv1000935	0.00	10.41	89.37	13.46	4.53	-	
PVITv1007159	0.00	0.00	74.51	13.62	46.73	-	conserved hypothetical protein
PVITv1013252	0.00	0.00	67.76	38.79	54.69	-	
PVITv1001768	0.00	33.00	21.99	13.79	93.67	-	
PVITv1016009	0.00	0.46	142.71	3.30	38.66	-	conserved hypothetical protein
PVITv1029584	0.00	58.71	34.38	79.22	24.92	-	
PVITv1028723	0.00	67.57	44.48	71.74	32.80	-	conserved hypothetical protein
PVITv1010696	0.00	0.00	74.37	91.87	61.87	-	putative GPI-anchored serine rich elicitin
PVITv1016027	0.00	147.68	54.50	113.28	46.55	-	
PVITv1017517	0.00	15.88	182.99	84.50	126.75	-	conserved hypothetical protein
PVITv1030765	0.00	0.00	158.87	153.80	101.44	-	carbohydrate-binding protein, putative
PVITv1004978	0.00	0.00	267.52	67.21	174.76	-	conserved hypothetical protein
PVITv1027001	0.00	16.18	471.71	67.34	130.09	-	

Supplementary Table S23. See attached Excel file "FPKM values of *P. viticola* secreted and non-secreted genes expressed during infection".

Supplementary Table S24. Effectors tested by *Agrobacterium* infiltration assays.

Effector gene ID	Symptoms on *V. vinifera*	Symptoms on *V. riparia*	Effector type
PVITv1003209	None	None	elicitin-like protein
PVITv1005727	None	None	elicitin-like INF6
PVITv1018092	None	None	Elicitin
PVITv1020941	None	None	Crinkler
PVITv1016922	None	None	Crinkler
PVITv1021061	None	None	RxLR
PVITv1008294	None	None	RxLR
PVITv1008311	None	Hypersensitive response	RxLR

Supplementary Table S25. Similarity search of the RxLR effector PVITv1_T008311 in the two other *P. viticola* genomes.

Query	Subject	Identity	AlignLen	Qend	Sstart	E-value
**PVITv1_T008311 vs INRA-PV221 (Dussert et al., 2016)**						
PVITv1_T008311	gi|1047461013|gb|MBPM01000113.1|	99.46	1308	1308	126397	0
PVITv1_T008311	gi|1047461013|gb|MBPM01000113.1|	76.97	330	325	169749	5.00E-44
PVITv1_T008311	gi|1047461013|gb|MBPM01000113.1|	78.87	265	271	133158	2.00E-42
PVITv1_T008311	gi|1047461013|gb|MBPM01000113.1|	78.47	274	280	139834	9.00E-42
PVITv1_T008311	gi|1047458994|gb|MBPM01001806.1|	99.85	689	1308	1	0
**PVITv1_T008311 vs PvitFEM01 (this work)**						
PVITv1_T008311	scaffold-5149	100	1308	1308	8731	0
PVITv1_T008311	scaffold-5149	78.87	265	271	1643	3E-42
PVITv1_T008311	scaffold-73	76.97	330	325	8478	6E-44
**PVITv1_T008311 vs JL-7-2 (Yin *et al.*, 2017)**						
PVITv1_T008311	MTPI01000176.1	99.9	994	1308	38702	0
PVITv1_T008311	MTPI01000176.1	100	173	226	39020	4E-86
PVITv1_T008311	MTPI01000176.1	78.49	265	271	31922	2E-40
PVITv1_T008311	MTPI01000176.1	100	37	292	38848	2E-10
PVITv1_T008311	MTPI01000890.1	77.81	329	325	34110	7E-49

Supplementary Table S26. See attached Excel file "KEGG modules significantly enriched or lost in *P. viticola*".

Supplementary Table S27*.* *P. infestans* KEGG orthologs in *P. viticola* isolates

KEGG Module	Pathway description	*P. infestans* KEGG gene ID	Blastn hits to find	
			**Orthologs in *P. viticola* INRA-PV221**	**Orthologs in P. viticola JL-7-2**
**M00531,M00615**	Nitrogen metabolism	PITG_13013	None	None
**M00176,M00616**	Sulphur assimilation	PITG_19263,PITG_18187	None	None
**M00027**	GABA shunt	PITG_01909	None	None
**M00028 (M00016,M00031)**	Ornithine biosynthesis	PITG_12053	None	None
**M00036 (M00088)**	Leucine degradation	PITG_00747	gi|1047460988|gb|MBPM01000132.1|	MTPI01000061.1
**M00051**	Uridine monophosphate biosynthesis	PITG_09635,PITG_09576	None	None
**M00307**	Pyruvate oxidation	PITG_03277, PITG_19161, PITG_06108, PITG_11929, PITG_00458, PITG_15359,PITG_18935, PITG_19802	gi|1047460982|gb|MBPM01000136.1|, gi|1047461157|gb|MBPM01000003.1|, gi|1047461135|gb|MBPM01000019.1|	MTPI01001062.1 MTPI01000086.1 MTPI01000266.1

Supplementary Table S28. See attached Excel file "FPKM values and GO term enrichment analysis of *Vitis vinifera* genes differentially expressed during infection".

Supplementary Table S29. RNA silencing proteins encoded in *P. viticola* genome.

Gene name	Predicted protein	Protein domains	Number of genes	*P. viticola* Gene ID
**PvRDR**	RNA-dependent RNA polymerase	RdRP (pfam05183), Helicase_C (pfam00271), DEXDc (cd00046), SSL2 (COG1061)	1	PVITv1_T028224
**PvAGO**	Argonaute	Piwi_ago-like (cd04657)	2	PVITv1_T036365
		Piwi_ago-like (cd04657), ArgoN (pfam16486), PAZ_argonaute_like (cd02846), ArgoL1 (pfam08699), PLN03202 (PLN03202)		PVITv1_T027285
**PvDRB**	dsRNA-binding	DSRM (cd00048), WW (smart00456)	1	PVITv1_T024270
**PvHEL**	RNA helicase	DEADc (cd00268), HELICc (cd00079), DEXDc (smart00487)	7	PVITv1_T035436
		DEADc (cd00268), DEXDc (smart00487)		PVITv1_T015528
		P-loop_NTPase super family (cl21455), Helicase_C (pfam00271), SrmB (COG0513)		PVITv1_T001847
		DEADc (cd00268), Helicase_C (pfam00271), SrmB (COG0513)		PVITv1_T002627
		Helicase_C (pfam00271), SrmB (COG0513)		PVITv1_T022767
		DEADc (cd00268), HELICc (cd00079), PTZ00424 (PTZ00424)		PVITv1_T018212
		P-loop_NTPase super family (cl21455), HELICc (cd00079), cas3_core (TIGR01587)		PVITv1_T033892
**PvHDAC**	Histone deacetylase	Arginase_HDAC super family (cl17011), PTZ00063 (PTZ00063)	5	PVITv1_T026116
		HDAC_classII_2 (cd11599)		PVITv1_T019080
		Arginase_HDAC super family (cl17011)		PVITv1_T034379
		Arginase_HDAC super family (cl17011)		PVITv1_T028430
		Arginase_HDAC super family (cl17011), PTZ00063 (PTZ00063)		PVITv1_T007443
**PvBRD**	Bromodomain	Bromodomain (cd04369), PHD_SF super family (cl22851)	1	PVITv1_T006879
**PvHMET**	SET (Su(var)3-9, Enhancer-of-zeste, Trithorax)	SET (smart00317), FYRN (pfam05964), FYRC super family (cl02651)	7	PVITv1_T027418
		SET (smart00317), AWS (smart00570), PostSET (smart00508)		PVITv1_T002061
		SET (smart00317)		PVITv1_T031084
		SET (smart00317), AWS (smart00570), PKc_like super family (cl21453), AWS (smart00570), RRM_SF super family (cl17169)		PVITv1_T003751
		SET (smart00317), PHD2_NSD (cd15565), PHD3_NSD (cd15566), PHD_SF super family (cl22851)		PVITv1_T019885
		SET (smart00317)		PVITv1_T031084
		SET (smart00317), PHD_SF super family (cl22851), AWS (smart00570)		PVITv1_T014932
**PvCRD**	Chromodomain	CHROMO (cd00024)	4	PVITv1_T029424
		Chromo (pfam00385)		PVITv1_T008234
		DEXDc (cd00046), Chromo (pfam00385), CHROMO (smart00298), DEXDc (smart00487)		PVITv1_T037120
		Chromo (pfam00385)		PVITv1_T015441
**PvRNaseIII**	Ribonuclease III	RIBOc (cd00593)	1	PVITv1_T011751
**PvDCL**	Dicer-like	Twice RIBOc (cd00593), Dicer_dimer super family (cl04028)	2	PVITv1_T038441
		DEAD (pfam00270), HELICc (smart00490), Dicer_dimer super family (cl04028), MPH1 (COG1111)		PVITv1_T003331

Supplementary Table S30. Effector genes associated with sRNAs.

Effector category[a]	*N unique sRNAs*	*Total N reads*	*N genes*	*N genes with sRNAs*	*Genes*
RxLR	240	21,147	202	3	PVITv1_T021061, T000764, **T024389**
Crinkler	416	119,424	285	13	PVITv1_T028898, T016878, T035967, T025173, T003247, T030506, T020941, T020942, T012549, **T024389**, T002897, T004916, T004903
YxSLK	8	200	308	5	PVITv1_T006273, T006256, T005164, T012793, T010063

[a]26 genes containing sRNAs mapping perfectly and uniquely on effector. For the RxLR the following variants were considered: RxLR+EER, SignalPeptide+RxLR+EER, SignalPeptide +RxLR-like(homology). For the CRN-like the following variants were considered: LFLAK+VVP, SignalPeptide +LFLAK, SVM. In particular, PVITv1_T024389 is present in both the CRN (it contains the LFLAK) and the RxLR (by homology) lists, and appeared to be a strong sRNA producer.

Supplementary Table S31. See attached Excel file “Degradome analysis of *V. vinifera* genes targeted by *V. vinifera* sRNAs”.

Supplementary Table S32. See attached Excel file “Degradome analysis of *P. viticola* genes targeted by *P. viticola* sRNAs”.

Supplementary Table S33. See attached Excel file “Degradome analysis of *V. vinifera* genes targeted by *P. viticola* sRNAs”.

Supplementary Table S34. See attached Excel file “Degradome analysis of *P. viticola* genes targeted by *V. vinifera* sRNAs”.

Supplementary Table S35. Oligonucleotide sequences.

Name	Sequence (5’-3’)	Purpose
F_PVITv1T003209_+sp_CACC	CACCATGGCAAAACTTTTCGTTGT	Agroinfiltration Elicitin
F_PVITv1T003209_Dp_CACC	CACCATGCACGACGGAGACGATGAC	“
R_PVITv1T003209	TTACGCTAGGATAGCGGC	“
F_PVITv1T005727_+sp_CACC	CACCATGAATCTATGCTTGACCATCG	Agroinfiltration Elicitin
F_PVITv1T005727_Dp_CACC	CACCATGAACGATTGTTCAGCAATTCAG	“
R_PVITv1T005727	TTAATCAGCACCACGAAAATTG	“
F_PVITv1T018092_+sp_CACC	CACCATGAACATCTTCTACGCTGTC	Agroinfiltration Elicitin
F_PVITv1T018092_Dp_CACC	CACCATGGAACCTTGCCCTCAAGATG	“
R_PVITv1T018092	TCAAAAGCTACGAAAAGAGTATG	“
F_PVITv1T020941	CACCATGAAGGACGCGATTGC	Agroinfiltration Crinkler
R_PVITv1T020941	TCAAGTGACGGTTGAC	“
F_PVITv1T016922	CACCATGATAGTAATGTGTGGTGAAGAAAAAG	Agroinfiltration Crinkler
R_PVITv1T016922	TCAGAGTGATTGCGTAAGCG	“
F_PVITv1T021061_+sp_CACC	CACCATGCAGCGCAAATGGC	Agroinfiltration RxLR
F_PVITv1T021061_Dp_CACC	CACCATGCTGTGCTGTGGTG	“
R_PVITv1T021061	CTACTCGTCCACCAAGATATAAC	“
F_PVITv1T008294_+sp_CACC	CACCATGCGCGGAAGTACG	Agroinfiltration RxLR
F_PVITv1T008294_Dp_CACC	CACCATGACTGCAATCGGAAAATCTCG	“
R_PVITv1T008294	CTAATTGCCGCCGCTC	“
F_RxLR_PVITv1T008311_+sp_CACC	CACCATGCGTGGTGCGTATTAC	Agroinfiltration RxLR
F_RxLR_PVITv1T008311_Dp_CACC	CACCATGTCTGACCGTCAGCTCC	“
R_RxLR_PVITv1T008311	TTACAAAGCTTTGTCAGTCC	“
F_pSKMCS_CACC	CACCTAATACGACTCACTATAGGGC	Agroinfiltration Empty vector
R_pSKMCS	TGACCATGATTACGCCAAGC	“
>FqRT_PVITv1T008311	CGCCTCCAAAATTGAAGGTCG	qRT-PCR RxLR_PVITv1T008311
>RqRT_ PVITv1T008311	GTTGGAAGACTGATTGTGCCG	“”
F_qRTGFPpK7WG2D	GACCACTACCAGCAGAACACC	qRT-PCR GFP
R_qRTGFPpK7WG2D	AGCTCGTCCTTCTTGTACAGC	
F_PveIF1b_ PVITv1_T004162	ACAACGGTGCAAGGCTTAGC	qRT-PCR house-keeping gene eiF1b
R_ PveIF1b_ PVITv1_T004162	ACTCGCGAATGTTAGTCCGC	“

SUPPLEMENTARY REFERENCES

1. Li, H. *et al.* The sequence alignment/map format and SAMtools. *Bioinformatics* **25**, 2078-2079 (2009).

2. Simpson, J.T. *et al.* ABySS: A parallel assembler for short read sequence data. *Genome Research* **19**, 1117-1123 (2009).

3. Stanke, M., Schöffmann, O., Morgenstern, B. & Waack, S. Gene prediction in eukaryotes with a generalized hidden Markov model that uses hints from external sources. *BMC Bioinformatics* **7**, 62 (2006).

4. Boisvert, S., Laviolette, F. & Corbeil, J. Ray: simultaneous assembly of reads from a mix of high-throughput sequencing technologies. *Journal of Computational Biology* **17**, 1519-1533 (2010).

5. Haas, B.J. *et al.* Genome sequence and analysis of the Irish potato famine pathogen *Phytophthora infestans*. *Nature* **461**, 393-398 (2009).

6. Keller, O., Odronitz, F., Stanke, M., Kollmar, M. & Waack, S. Scipio: Using protein sequences to determine the precise exon/intron structures of genes and their orthologs in closely related species. *BMC Bioinformatics* **9**, 278 (2008).

7. Slater, G.S.C. & Birney, E. Automated generation of heuristics for biological sequence comparison. *BMC Bioinformatics* **6**, 31 (2005).

8. Langmead, B. & Salzberg, S.L. Fast gapped-read alignment with Bowtie 2. *Nat Meth* **9**, 357-359 (2012).

9. Majoros, W.H., Pertea, M. & Salzberg, S.L. TigrScan and GlimmerHMM: two open source ab initio eukaryotic gene-finders. *Bioinformatics* **20**, 2878-2879 (2004).

10. Parra, G., Blanco, E. & Guigó, R. GeneID in Drosophila. *Genome Research* **10**, 511-515 (2000).

11. Moriya, Y., Itoh, M., Okuda, S., Yoshizawa, A.C. & Kanehisa, M. KAAS: an automatic genome annotation and pathway reconstruction server. *Nucleic Acids Research* **35**, W182-W185 (2007).

12. Falda, M. *et al.* Argot2: a large scale function prediction tool relying on semantic similarity of weighted Gene Ontology terms. *BMC Bioinformatics* **13**, S14 (2012).

13. Fontana, P., Cestaro, A., Velasco, R., Formentin, E. & Toppo, S. Rapid annotation of anonymous sequences from genome projects using semantic similarities and a weighting scheme in gene ontology. *PLoS ONE* **4**, e4619 (2009).

14. Remm, M., Storm, C.E.V. & Sonnhammer, E.L.L. Automatic clustering of orthologs and in-paralogs from pairwise species comparisons. *Journal of Molecular Biology* **314**, 1041-1052 (2001).

15. Trapnell, C. *et al.* Differential gene and transcript expression analysis of RNA-seq experiments with TopHat and Cufflinks. *Nat. Protocols* **7**, 562-578 (2012).

16. Lagesen, K. *et al.* RNAmmer: consistent and rapid annotation of ribosomal RNA genes. *Nucleic Acids Research* **35**, 3100-3108 (2007).

17. Lowe, T.M. & Eddy, S.R. tRNAscan-SE: A program for improved detection of transfer RNA genes in genomic sequence. *Nucleic Acids Research* **25**, 955-964 (1997).

18. Voglmayr, H. & Greilhuber, J. Genome size determination in Peronosporales (Oomycota) by Feulgen image analysis. *Fungal Genetics and Biology* **25**, 181-195 (1998).

19. Simão, F.A., Waterhouse, R.M., Ioannidis, P., Kriventseva, E.V. & Zdobnov, E.M. BUSCO: assessing genome assembly and annotation completeness with single-copy orthologs. *Bioinformatics* **31**, 3210-3212 (2015).

20. Alexeyenko, A., Tamas, I., Liu, G. & Sonnhammer, E.L.L. Automatic clustering of orthologs and inparalogs shared by multiple proteomes. *Bioinformatics* **22**, e9-15 (2006).

21. Katoh, K. & Standley, D.M. MAFFT multiple sequence alignment software version 7: improvements in performance and usability. *Molecular Biology and Evolution* **30**, 772-780 (2013).

22. Talavera, G. & Castresana, J. Improvement of phylogenies after removing divergent and ambiguously aligned blocks from protein sequence alignments. *Systematic Biology* **56**, 564-577 (2007).

23. Castresana, J. Selection of conserved blocks from multiple alignments for their use in phylogenetic analysis. *Molecular Biology and Evolution* **17**, 540-552 (2000).

24. Guindon, S. *et al.* New algorithms and methods to estimate maximum-likelihood phylogenies: assessing the performance of PhyML 3.0. *Systematic Biology* **59**, 307-321 (2010).

25. Stamatakis, A. RAxML version 8: a tool for phylogenetic analysis and post-analysis of large phylogenies. *Bioinformatics* **30**, 1312-1313 (2014).

26. Sharma, R. *et al.* Genome analyses of the sunflower pathogen *Plasmopara halstedii* provide insights into effector evolution in downy mildews and *Phytophthora*. *BMC Genomics* **16**, 741 (2015).

27. McCarthy, C.G.P. & Fitzpatrick, D.A. Phylogenomic reconstruction of the oomycete phylogeny derived from 37 genomes. *mSphere* **2**, e00095-17 (2017).

28. Tian, M. *et al.* 454 genome sequencing of *Pseudoperonospora cubensis* reveals effector proteins with a QXLR translocation motif. *Molecular Plant-Microbe Interactions* **24**, 543-553 (2011).

29. Lévesque, C.A. *et al.* Genome sequence of the necrotrophic plant pathogen *Pythium ultimum* reveals original pathogenicity mechanisms and effector repertoire. *Genome Biology* **11**, R73 (2010).

30. Bailey, T.L. & Elkan, C. Fitting a mixture model by expectation maximization to discover motifs in biopolymers. in *Proc Int Conf Intell Syst Mol Biol* Vol. 2 28-36 (1994).

31. Finn, R.D. *et al.* Pfam: the protein families database. *Nucleic Acids Research* **42**, D222-D230 (2014).

32. Eddy, S.R. Profile hidden Markov models. *Bioinformatics* **14**, 755-763 (1998).

33. Trapnell, C. *et al.* Transcript assembly and quantification by RNA-Seq reveals unannotated transcripts and isoform switching during cell differentiation. *Nat. Biotechnol.* **28**, 511-515 (2010).

34. Zottini, M. *et al.* Agroinfiltration of grapevine leaves for fast transient assays of gene expression and for long-term production of stable transformed cells. *Plant Cell Reports* **27**, 845-853 (2008).

35. Roetschi, A., Si-Ammour, A., Belbahri, L., Mauch, F. & Mauch-Mani, B. Characterization of an Arabidopsis-*Phytophthora* pathosystem: resistance requires a functional *PAD2* gene and is independent of salicylic acid, ethylene and jasmonic acid signalling. *The Plant Journal* **28**, 293-305 (2001).

36. Pfaffl, M.W. A new mathematical model for relative quantification in real-time RT–PCR. *Nucleic Acids Research* **29**, e45 (2001).

37. Takami, H. *et al.* An automated system for evaluation of the potential functionome: MAPLE version 2.1.0. *DNA Research* **23**, 467-475 (2016).

38. Zheng, Y., Li, Y.-F., Sunkar, R. & Zhang, W. SeqTar: an effective method for identifying microRNA guided cleavage sites from degradome of polyadenylated transcripts in plants. *Nucleic Acids Research* **40**, e28 (2011).

39. Petersen, T.N., Brunak, S., von Heijne, G. & Nielsen, H. SignalP 4.0: discriminating signal peptides from transmembrane regions. *Nat Meth* **8**, 785-786 (2011).

40. Boutemy, L.S. *et al.* Structures of *Phytophthora* RXLR effector proteins: a conserved but adaptable fold unperpins functional diversity. *Journal of Biological Chemistry* **286**, 35834-35842 (2011).

41. Fahlgren, N. *et al.* *Phytophthora* have distinct endogenous small RNA populations that include short interfering and microRNAs. *PLoS ONE* **8**, e77181 (2013).
